# Genome of *Acanthamoeba castellanii *highlights extensive lateral gene transfer and early evolution of tyrosine kinase signaling

**DOI:** 10.1186/gb-2013-14-2-r11

**Published:** 2013-02-01

**Authors:** Michael Clarke, Amanda J Lohan, Bernard Liu, Ilias Lagkouvardos, Scott Roy, Nikhat Zafar, Claire Bertelli, Christina Schilde, Arash Kianianmomeni, Thomas R Bürglin, Christian Frech, Bernard Turcotte, Klaus O Kopec, John M Synnott, Caleb Choo, Ivan Paponov, Aliza Finkler, Chris Soon Heng Tan, Andrew P Hutchins, Thomas Weinmeier, Thomas Rattei, Jeffery SC Chu, Gregory Gimenez, Manuel Irimia, Daniel J Rigden, David A Fitzpatrick, Jacob Lorenzo-Morales, Alex Bateman, Cheng-Hsun Chiu, Petrus Tang, Peter Hegemann, Hillel Fromm, Didier Raoult, Gilbert Greub, Diego Miranda-Saavedra, Nansheng Chen, Piers Nash, Michael L Ginger, Matthias Horn, Pauline Schaap, Lis Caler, Brendan J Loftus

**Affiliations:** 1 Conway Institute, University College Dublin, Belfield, Dublin 4, Ireland; 2 Samuel Lunenfeld Research Institute, Mount Sinai Hospital, 600 University Ave, Room 1081 Toronto, Ontario M5G 1X5, Canada; 3 Department für Mikrobielle Ökologie, Universität Wien, Althanstr. 14, A-1090 Wien, Austria; 4 Department of Biology, San Francisco State University, 1600 Holloway Ave, San Francisco, CA 94132, USA; 5 Bioinformatics Department, J Craig Venter Institute, Inc., 9704 Medical Center DriveRockville, MD 20850, USA; 6 Center for Research on Intracellular Bacteria, Institute of Microbiology, Institute of Microbiology, Rue du Bugnon 48, 1011 Lausanne, Switzerland; 7 College of Life Sciences, University of Dundee, Dow Street, Dundee DD1 5EH, UK; 8 Institute of Biology, Experimental Biophysics, Humboldt-Universität zu Berlin, Invalidenstrasse 42, D-10115 Berlin, Germany; 9 Department of Biosciences and Nutrition and Center for Biosciences, Karolinska Institutet, Hälsovägen 7, Novum, SE 141 83 Huddinge, Sweden; 10 Department of Molecular Biology and Biochemistry, Simon Fraser University, 8888 University Drive, Burnaby, BC V5A 1S6, Canada; 11 Department of Medicine, McGill University, McIntyre Medical Building, 3655 Sir William Osler, Montreal, Quebec H3G 1Y6, Canada; 12 Max Planck Institute for Developmental Biology, Spemannstr. 35 - 39, 72076 Tübingen, Germany; 13 Institut für Biologie II/Molecular Plant Physiology, Faculty of Biology, Albert-Ludwigs University of Freiburg, Freiburg, Germany; 14 Department of Plant Sciences, Britannia 04, Tel-Aviv University, Tel-Aviv 69978, Israel; 15 CeMM-Research Center for Molecular Medicine of the Austrian Academy of Sciences, Lazarettgasse 14, AKH BT 25.3, A-1090 Vienna, Austria; 16 World Premier International (WPI) Immunology Frontier Research Center (IFReC), Osaka University, 3-1 Yamadaoka, Suita, 565-0871 Osaka, Japan; 17 Department für Computational Systems Biology, Universität Wien, Althanstraße 14, 1090 Wien, Austria; 18 Department of Medical Genetics, Medical Genetics, C201 - 4500 Oak Street, Vancouver, BC, V6H 3N1, Canada; 19 Unité des rickettsies, IFR 48, CNRS-IRD UMR 6236, Faculté de médecine, Université de la Méditerranée, Marseille, France; 20 Banting and Best Department of Medical Research, Donnelly Centre, University of Toronto, 160 College Street, Room 230,Toronto, Ontario M5S 3E1, Canada; 21 Institute of Integrative Biology, Biosciences Building, University of Liverpool, Crown Street, Liverpool L69 7ZB, UK; 22 Department of Biology, NUI Maynooth, Co Kildare, Ireland; 23 University Institute of Tropical Diseases and Public Health of the Canary Islands, University of La Laguna, Avda. Astrofísico Fco. Sánchez, S/N 38203 La Laguna, Tenerife, Canary Islands, Spain; 24 Wellcome Trust Sanger Institute, Wellcome Trust Genome Campus, Hinxton, Cambridge CB10 1SA, UK; 25 Divisions of Pediatric Infectious Diseases, Department of Pediatrics, Chang Gung Children's Hospital and Chang Gung Memorial Hospital, Taoyuan, Taiwan; 26 Department of Parasitology, Chang Gung University, Taoyuan, Taiwan; 27 Ben May Department for Cancer Research and Committee on Cancer Biology, The University of Chicago, Chicago, IL 60637, USA; 28 Faculty of Health and Medicine, Division of Biomedical and Life Sciences, Lancaster University, Lancaster, LA1 4YQ, UK

## Abstract

**Background:**

The Amoebozoa constitute one of the primary divisions of eukaryotes, encompassing taxa of both biomedical and evolutionary importance, yet its genomic diversity remains largely unsampled. Here we present an analysis of a whole genome assembly of *Acanthamoeba castellanii *(*Ac*) the first representative from a solitary free-living amoebozoan.

**Results:**

*Ac *encodes 15,455 compact intron-rich genes, a significant number of which are predicted to have arisen through inter-kingdom lateral gene transfer (LGT). A majority of the LGT candidates have undergone a substantial degree of intronization and *Ac *appears to have incorporated them into established transcriptional programs. *Ac *manifests a complex signaling and cell communication repertoire, including a complete tyrosine kinase signaling toolkit and a comparable diversity of predicted extracellular receptors to that found in the facultatively multicellular dictyostelids. An important environmental host of a diverse range of bacteria and viruses, *Ac *utilizes a diverse repertoire of predicted pattern recognition receptors, many with predicted orthologous functions in the innate immune systems of higher organisms.

**Conclusions:**

Our analysis highlights the important role of LGT in the biology of *Ac *and in the diversification of microbial eukaryotes. The early evolution of a key signaling facility implicated in the evolution of metazoan multicellularity strongly argues for its emergence early in the Unikont lineage. Overall, the availability of an *Ac *genome should aid in deciphering the biology of the Amoebozoa and facilitate functional genomic studies in this important model organism and environmental host.

## Background

*Acanthamoeba castellanii *(*Ac*) is one of the predominant soil organisms in terms of population size and distribution, where it acts both as a predator and an environmental reservoir for a number of bacterial, fungal and viral species [1]. Selective grazing by *Ac *in the rhizosphere alters microbial community structure and is an important contributor to the development of root architecture and nutrient uptake by plants [2]. *Ac *can also be isolated from almost any body of water and manifests in a wide variety of man-made water systems, including potable water sources, swimming pools, hot tubs, showers and hospital air conditioning units [3,4]. Acanthamoebae are frequently associated with a diverse range of bacterial symbionts [5,6]. A subset of the microbes that serve as prey for *Ac *have evolved virulence stratagems to use *Ac *as both a replicative niche and as a vector for dispersal and are important human intracellular pathogens [7,8]. These pathogens utilize analogous strategies to infect and persist within mammalian macrophages, illustrating the role of environmental hosts such as *Ac *in the evolution and maintenance of virulence [9,10]. Commonalities at the level of host response between amoebae and macrophages to such pathogens have led to the use of both *Dictyostelium discoideum *(*Dd*) and *Ac *as model systems to study pathogenesis [11,12].

Published Amoebozoa genomes from both the obligate parasite *Entamoeba histolytica *(*Eh*) and the facultatively multicellular *Dd *have both highlighted unexpected complexities at the level of cell motility and signaling [13,14]. As the only solitary free-living representative, the genome of *Ac *establishes a unique reference point for comparisons for the interpretation of other amoebozoan genomes. Experimentally, *Ac *has been a more thoroughly studied organism than most other free living amoebae, acting as a model organism for studies on the cytoskeleton, cell movement, and aspects of gene regulation, with a large body of literature supporting its molecular interactions [15-18].

## Results and discussion

### Lateral gene transfer

**Figure 1:**
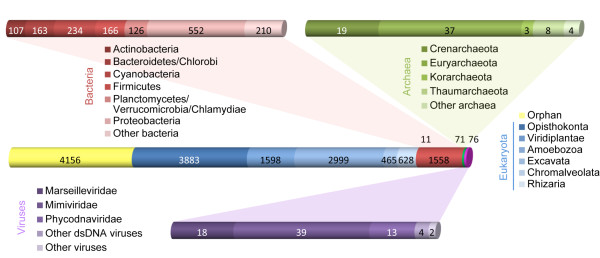
**Measures of the composition of the *Ac *genome based on sequence similarity**. For each protein, the best BLASTP hit to the non-redundant database, that is, the match with the lowest e-value, was recovered and the classification of the corresponding organism was extracted according to NCBI taxonomy. The central bar represents the full complement of annotated *Ac *genes exhibiting a best BLASTP hit respectively against the four kingdoms - Eukaryota (blue), Bacteria (red), Archaea (green) and viruses (purple) - with orphan genes depicted in yellow. Results for Eukaryota are subdivided according to the major taxonomic phyla in varying shades of blue. Subdivisions of phyla within the Bacteria (red shading), Archaea (green shading) and viruses (purple shading) are depicted in the expanded upper and lower sidebars. dsDNA, double-stranded DNA.

Lateral gene transfer (LGT) is considered a key process of genome evolution and several studies have indicated that phagotrophs manifest an increased rate of LGT compared to non-phagotrophic organisms [19]. As a geographically dispersed bacteriovorous amoebae with a penchant for harboring endosymbionts, *Ac *encounters a rich and diverse supply of foreign DNA, providing ample opportunity for LGT. Homology-based searches of the proteome illustrate the potential for diverse contributions to the genome (Figure 1).

**Figure 2:**
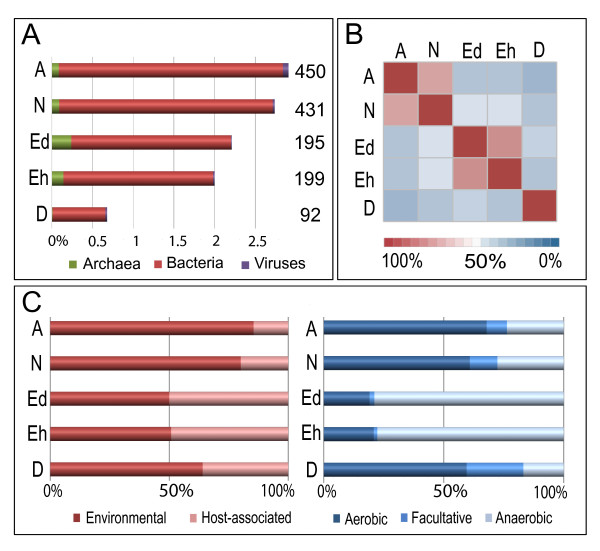
**Predicted LGT-derived genes from Bacteria, Archaea and viruses encoded in the genomes of free-living and parasitic amoebae**. LGT-derived genes were predicted using a phylogenomics approach consisting of an initial similarity-based screening using SIMAP [111], several filtering steps to extract amoebal proteins with prokaryotic best hits, followed by automatic calculation and manual inspection of phylogenetic trees using PhyloGenie and PHAT [112]. **(a) **Percentage of lineage-specific LGT candidates in each genome; the absolute number of LGT candidates per genome is indicated next to each bar. **(b) **Heat map illustrating the Bray-Curtis similarity of the taxonomic affiliation (at the level of classes within the domain Bacteria) of putative LGT donors. **(c) **Ecological classification of putative LGT donors with respect to their oxygen requirement and association with a host. The ecology of putative donors was extrapolated from the lifestyles of the respective closest extant relatives.

We therefore undertook a phylogenomic analysis to determine cases of predicted inter-domain LGT in the *Ac *genome (Section 2 of Additional file 1). Our analysis identified 450 genes, or 2.9% of the proteome, predicted to have arisen through LGT (Figure 2; Section 2 of Additional file 1). To determine the fate and ultimate utility of the LGT candidates within the *Ac *genome, we examined their expression levels across a number of experimental conditions using RNA.seq (Table S1.6.1 in Additional file 1). Our results show that most of the LGT candidates are expressed in at least some of the conditions tested (Additional file 2).

Genetic exchange is also thought to occur between phylogenetically disparate organisms that reside within the same amoebal host cell [20,21]. *Ac *contains three copies of a miniature transposable element (ISSoc2) of the IS607 family of insertion sequences related to those present in genomes of thermophilic cyanobacteria [22] and several giant nucleocytoplasmic large DNA viruses (NCLDVs). In the Mimivirus genome the IS elements are found within islands of genes of bacterial origin, some of which appear to have been contributed by a cyanobacterial donor. This data underscores the complex intermediary role that *Ac*, as host to both NCLDVs and cyanobacteria [17] may play in facilitating genetic transfer between sympatric species.

### Comparison of predicted LGT across amoeboid genomes

In order to compare the impact and scale of LGT across *Ac *and other amoeba, we applied the same phylogenomic approach used to identify LGT in the *Ac *genome to published genomes of other amoeboid protists, including *Dd*, *Eh*, *Entamoeba dispar *(*Ed*) and *Naegleria gruberi *(*Ng*). Our findings predict that *Ac *and the excavate *Ng *encode a notably higher number of laterally acquired bacterial genes than either of the more closely related parasitic *Entamoeba *or the social *Dd *amoebozoans (Figure 2a). The taxonomic distribution of putative LGT donors is broadly similar for both *Entamoeba *species, but surprisingly also between *Ac *and *Ng *(Figure 2b,c; Section 2 of Additional file 1). The genomes of both *Eh *and *Ed *are predicted to have experienced a proportionately higher influx from anaerobic and host-associated microbes than their free-living counterparts *Ac *and *Ng *(Figure 2c; Additional file 2), likely reflecting the composition of microbes within their habitats. Many of the LGT candidates across all of the amoebae have predicted metabolic functions, suggesting that LGT in amoebae is reflective of trophic strategy and driven by the selective pressure of new ecological niches. Our data illustrating LGT as a contributing factor in shaping the biology of a diversity of amoeboid genomes provide further evidence supporting an underappreciated role for LGT in the diversification of microbial eukaryotes [23].

### Introns

Intron-exon structures exhibit complex phylogenetic patterns with orders-of-magnitude differences across eukaryotic lineages, which imply frequent transformations during eukaryotic evolution [24]. Some researchers have argued that intron gain is episodic with long periods of stasis [25] punctuated by periods of rapid gain while others argue for generally higher rates [26]. Strikingly, *Ac *genes have an average of 6.2 introns per gene, among the highest known in eukaryotes [27]. Genes predicted to have arisen through LGT have slightly lower but broadly comparable intron densities, offering an opportunity to study the evidence for proposed mechanisms underpinning post-LGT intron gain [28]. An analysis of LGT introns, however, did not provide support for any of the proposed mechanisms of intron gain (Section 2 of Additional file 1). Thus, while the preponderance of introns in LGTs clearly indicates substantial intron gain at some point, it appears that, for *Ac*, these events have been very rare in recent times, consistent with a punctate model of intron gain.

### Cell signaling

As a unicellular sister grouping to the multicellular Dictyostelids, *Ac *provides a unique point of comparison to gain insight into the molecular underpinnings of multicellular development in Amoebozoa. Cell-cell communication is a hallmark of multicellularity and we looked at putative receptors for extracellular signals and their downstream targets. G-protein-coupled receptors (GPCRs) represent one of the largest families of sensors for extracellular stimuli. Overall, *Ac *encodes 35 GPCRs (compared to 61 in *Dd*), representing 4 out of the 6 major families of GPCRs [29] while lacking metabotropic glutamate-like GPCRs or fungal pheromone receptors. We identified three predicted fungal-associated glucose-sensing Git3 GPCRs [30] and an expansion in the number of frizzled/smoothened receptors [31] (Figure S3.1.1 in Additional file 1). We identified seven G-protein alpha subunits and a single putative target, phospholipase C, for GPCR-mediated signaling. The number and diversity of receptors in *Ac *raises the question of what they are likely to be sensing. Nematodes employ many of their GPCRs in detecting molecules secreted by their bacterial food sources [32], and given the diversity of *Ac*'s feeding environments, many of the *Ac *GPCRs may fulfill a similar role.

### Environmental sensing

**Figure 3:**
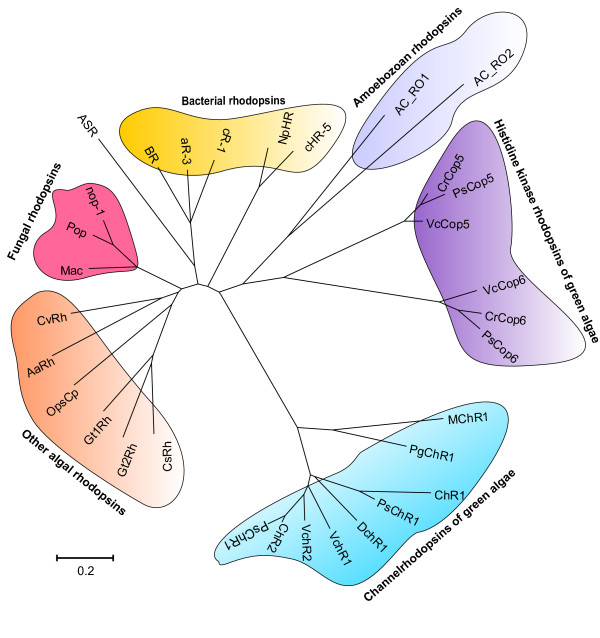
**Phylogenetic tree of rhodopsins from Amoebozoa, algae, bacteria and fungi**. The tree was constructed by the neighbor-joining method based on the amino acid sequence of the rhodopsin domain using MEGA version 5 [113]. The scale bar indicates the number of substitutions per site. Detailed rhodopsin information is listed in Table S3.6.1 in Additional file 1.

We identified 48 sensor histidine kinases (SHKs), of which 17 harbor transmembrane domains and may function as receptors (Figure S3.2.1 in Additional file 1). Remarkably, there are also 67 nucleotidyl cyclases consisting of an extracellular receptor domain separated by a single transmembrane helix from an intracellular cyclase domain flanked by two serine/threonine kinase domains. This domain configuration is present in a number of the amoeba-infecting giant viruses but thus far appears unique for a cellular organism (Figure S3.3.1 in Additional file 1). *Ac *is able to survive under microaerophilic conditions such as those found in the deeper layers of underwater sediments or within the rhizosphere. The genome encodes a number of prolyl 4-hydroxylases that likely mediate oxygen response; however, *Ac *also contains a number of heme-nitric oxide/oxygen binding (H-NOX) proteins that, unlike those in other eukaryotes, are not found in conjunction with guanylyl cyclases [33]. The *Ac *H-NOX proteins lack a critical tyrosine residue in the non-polar distal heme pocket, making it likely that they are for nitric oxide (NO) rather than oxygen signaling [34]. Both *Dd *and *Ac *are responsive to light, although the photoreceptor that mediates phototaxis in *Dictyostelium *has yet to be identified [35]. We identified two rhodopsins both with carboxy-terminal histidine kinase and response regulator domains with homology to the sensory rhodopsins of the green algae that represent candidates for light sensors in *Ac *(Figure 3).

### Cellular response

**Figure 4:**
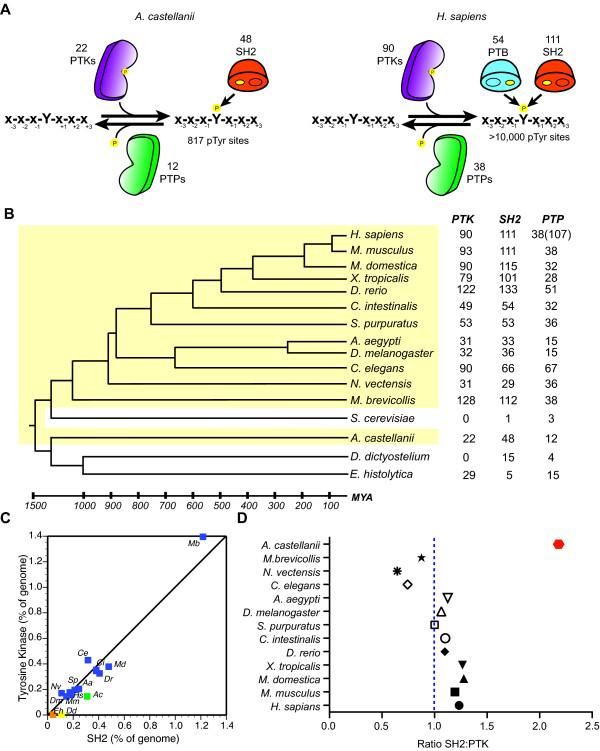
**The phosphotyrosine signaling circuitry of *Ac***. **(a) **Phosphotyrosine signaling is modulated by the writers (PTKs), erasers (PTPs) and readers (Src homology 2 (SH2); phosphotyrosine binding (PTB)). **(b) **The total number of PTKs, classical PTPs (total PTPs) and SH2 encoded genes across multiple eukaryote genomes. Highlighted in yellow are branches that compose a complete phosphotyrosine signaling circuit. The branched divergence times and lengths in millions of years (mya) are indicated. **(c) **The percentage of the genome devoted to encoding PTKs and SH2 domains. **(d) ***Ac *displays the greatest ratio of SH2:PTKs compared to other eukaryotes.

Modulation of cellular response to environmental cues is enacted by a diversity of protein kinases and *Ac *is predicted to encode 377, the largest number predicted to date for any amoebozoan (Section 4 of Additional file 1). In *Ac*, the mitogen-activated protein kinase (MAPK) kinase pathway has been shown to be involved in encystment [36] and its genome encodes homologues of both of *Dd*'s two MAPK proteins, ErkA and ErkB [37]. Phosphotyrosine (pTyr) signaling mediated through tyrosine kinases was until recently thought to be generally absent from the amoebozoan lineage [38]. This signaling capacity has been associated with intercellular communication, the evolutionary step towards multicellularity and the expansion of organismal complexity in metazoans [39]. pTyr is thought to depend upon a triad of signaling molecules; tyrosine kinase 'writers' (PTKs), tyrosine phosphatase 'erasers' (PTPs) and Src homology 2 (SH2) 'reader' domains that connect the phosphorylated ligand-containing domains to specify downstream signaling events [39]. Remarkably, the genome of *Ac *encodes 22 PTKs, 12 PTPs, and 48 SH2 domain-containing proteins (Figure 4a), revealing a primordial yet elaborate pTyr signaling system in the amoebozoan lineage (Figure 4b).

The *Ac *PTK domains are highly conserved in key catalytic residues, resembling dedicated PTKs found in metazoans (Figure S4.2.1 in Additional file 1), and are distinct from *Dd *and *Eh *PTKs that are more tyrosine kinase like (TKL) (Figure S4.2.2 in Additional file 1). *Ac *PTK homologues are present in the apusomonad *Thecamonas trahens *and have also recently been described in two filasterean species, *Capsaspora owczarzaki *and *Ministeria vibrans *[38]. One unusual feature of the pTyr machinery in *Ac *is the 2:1 ratio of SH2 to PTK domains as comparisons across opisthokonts show a strong correlation and co-expansion of these two domains with a ratio close to 1:1 (Figure 4c,d) [40]. This increased ratio in *Ac *indicates either an expansion to handle the cellular requirements of pTyr signaling or that aspects of PTK function are accomplished by TKL or dual specificity kinases as appears to be the case in *Dd *[41]. We also found that *Ac *has fewer tyrosine residues in its proteome in comparison to *Dd*, which lacks PTKs (Figure S4.3.1 in Additional file 1). This result is in line with recent analysis of metazoan genomes, suggesting increased pressure for selection against disadvantageous phosphorylation of tyrosine residues in genomes with extensive pTyr signaling [42].

Domain organization and composition of pTyr components reveal the selective pressures for adapting pTyr signaling into various pathways. Seven PTKs have predicted transmembrane domains and may function as receptor tyrosine kinases hinting at their potential for intercellular communication. The majority of PTKs in *Ac*, however, show unique domain combinations; six PTKs contain a sterile alpha motif (SAM) domain, which is found in members of the ephrin receptor family (Figure S4.4.3 in Additional file 1). The *Ac *SH2 proteins are conserved within the pTyr binding pocket and resemble SH2 domains from the SOCS, RIN, CBL and RASA families (Figure S4.4.2 in Additional file 1); however, the domain composition within these proteins differs between those of *Monosiga brevicollis *and metazoans (Figure S4.4.3A in Additional file 1). Approximately half of the *Ac *SH2 proteins share domain architectures with *Dd*, including the STAT family of transcription factors (Figure S4.4.3B in Additional file 1). The presence of homologous SH2 proteins in *Dd *coupled with the complete facility in *Ac *predicts an emergence of the complete machinery for pTyr early in the Unikont lineage. This finding is in contrast with models that posit a complete pTyr signaling machinery emerging late in the Unikont lineage [39] and has important implications for understanding the relationship between pTyr signaling and the evolution of multicellularity. The lack of clear metazoan orthologues makes it difficult to trace the evolutionary paths of pTyr signaling networks [43] or to accurately predict the cellular functions and adaptations of pTyr in *Ac*. However, with phosphoproteomics and sequence analysis, insights into ancient pTyr signaling circuits may be revealed through future studies in *Ac *(Figure S4.5.1 in Additional file 1).

### Cell adhesion

*Ac *is not known to participate in social activity yet must adhere to a diversity of surfaces within the soil and practice discrimination between self and prey during phagocytosis [44]. *Ac *shares some adhesion proteins with *Dd *(Table S5.1.1 in Additional file 1) but homologues of the calcium-dependent, integrin-like Sib cell-adhesion proteins are absent. Surprisingly, *Ac *contains a number of bacterial-like integrin and hemagglutinin domain adhesion proteins that may improve its ability to attach to bacterial cells or biofilms [45]. *Ac *encodes two MAM domain-containing proteins, a domain found in functionally diverse receptors with roles in cell-cell adhesion [46]. *Ac *has a copy of the laminin-binding protein (AhLBP) first identified in *Acanthamoeba healyi*, which has been shown to act as a non-integrin laminin binding receptor [47]. Remarkably, *Ac *also encodes proteins containing cell adhesion immunoglobulin domains (Section 5 of Additional file 1). Both show affinity to the I-set subfamily [48] and contain weakly predicted transmembrane domains (Figure S5.1.1 in Additional file 1).

### Microbial recognition through pattern recognition receptors

**Figure 5:**
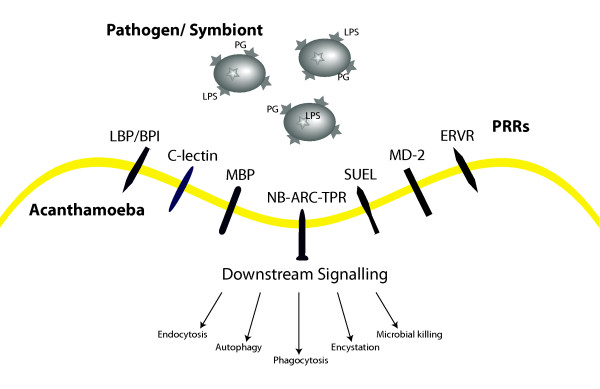
**Potential PRRs in *Ac*: LBP/BPI, lipopolysaccharide binding protein/ bactericidal permeability-increasing protein; C-lectin, C-type lectin: MBP, mannose binding protein; SUEL, D-galactoside/L-rhamnose binding SUEL lectin domain containing; NB-ARC-TPR, NB-ARC tetratricopeptide repeat containing protein; ERVR, endogenous virus receptor**.

*Ac *grazes on a variety of micro fauna, which requires the mobilization of a set of defense responses initiated upon microbial recognition. In vertebrates molecular signatures often termed microbe-associated molecular patterns (MAMPs) [49] are detected by pattern-recognition receptors (PRRs) that activate downstream transcriptional responses. As *Ac *practices selective feeding behavior we looked for the presence of predicted PRRs in the *Ac *genome (Figure 5). One of the best-studied MAMPs is lipopolysaccharide and discrimination mediated through lectin-mediated protein-carbohydrate interactions is an important innate immunity strategy in both vertebrates and invertebrates [50]. *Ac *contains six members of the bactericidal permeability-increasing protein (BPI)/lipopolysaccharide-binding protein (LBP) family and two peptidoglycan binding proteins (Figure 5; Section 6 of Additional file 1). *Ac *also encodes a membrane bound homologue of an MD-2-related protein that, in vertebrate immunity, has been implicated in opsonophagocytosis of Gram-negative bacteria through its interactions with lipopolysaccharide [51].

Receptor-mediated endocytosis of *Legionella pneumophila *in *Ac *is mediated by the c-type lectin mannose binding protein (MBP) [52]. MBP also represents the principal virulence factor in pathogenic Acanthamoebae [53]. In addition to MBP, the *Ac *genome encodes two paralogues of MBP with similarity to the amino-terminal region of the protein. Rhamnose-binding lectins serve a variety of functions in invertebrates, one of which is their role as germline-encoded PRRs in innate immunity [54]. They are absent from other Amoebozoa, although *Ac *encodes 11 D-galactoside/L-rhamnose binding (SUEL) lectin domain-containing proteins. Approximately half of the SUEL lectin domain proteins harbour epidermal growth factor domains, a combination reminiscent of the selectin family of adhesion proteins found exclusively in vertebrates [55]. An L-rhamnose synthesis pathway thought to contribute to biosynthesis of the lipopolysaccharide-like outer layer of the virus particle has recently been identified in Mimivirus that may facilitate its uptake by *Ac *[56,57]. *Ac *also encodes a protein where multiple copies of H-type lectin are joined with an inhibitor of apoptosis domain. The H-lectin domain is predicted to bind to N-acetylgalactosamine (GalNAc) and is found in *Dictyostelium *discoidin I & II [58] and other invertebrates where it plays a role in antibacterial defense [59]. In the brown algae *Ectocarpus *leucine-rich repeat (LRR) containing GTPases of the ROCO family and NB-ARC-TPR proteins have been proposed to represent PRRs that are involved in immune response [60]. *Ac *encodes a NB-ARC-TPR homologue with a disease resistance domain (IPR000767) and an LRR-ROCO GTPase.

### Antimicrobial defense

*Ac *encodes proteins with potential roles in antiviral defense including homologues of NCLDV major capsid proteins [61] as well as homologues of Dicer and Piwi, both of which have been implicated in RNA-mediated antiviral silencing [62]. Our data also illustrate early evolution of a number of interferon-inducible innate immunity proteins absent from other sequenced Amoebozoa. These include a homologue of the interferon-γ-inducible lysosomal thiol reductase enzyme (GILT), an important host factor targeted by *Listeria monocytogenes *during infection in macrophages [63]. In addition, *Ac *encodes two interferon-inducible GTPase homologues, which in vertebrates promote cell-autonomous immunity to vacuolar bacteria, including *Mycobacteria *and *Legionella *species [64]. *Ac *also contains a natural resistance-associated macrophage protein (NRAMP) homologue, which has been implicated in protection against *L. pneumophila *and *Mycobacterium avium *infection in both macrophages and *Dd *[65].

### Metabolism

*Ac *has traditionally been considered to be an obligate aerobe, although the recent identification of the oxygen-labile enzymes pyruvate:ferredoxin oxidoreductase and FeFe-hydrogenase perhaps pointed towards a cryptic capacity for anaerobic ATP production [66]. Predictions for nitrite and fumarate reduction, hydrogen fermentation, together with a likely mechanism for acetate synthesis, coupled to ATP production indicate a considerable capacity for anaerobic ATP generation. This clearly sets *Ac *apart from *Dd*, which hunts within the aerobic leaf litter, but provides parallels with *Ng*, the alga *Chlamydomonas reinhardtii *and other soil-dwelling protists that are likely to experience considerable variation in local oxygen tensions [67]. These protists achieve their flexible, facultative anaerobic metabolism, however, using different pathways (Figure S7.1 in Additional file 1). In addition, the classic anaerobic twists on glycolysis provided by pyrophosphate-dependent phosphofructokinase and pyruvate phosphate dikinase [68] are absent from *Ac*. This suggests that although multiple pathways are available for oxidation of NADH to NAD+ in the absence of oxygen, including a capacity for anaerobic respiration in the presence of nitrite (NO_2_^-^), a shift to a more ATP-sparing form of glycolysis is not necessary under low oxygen-tension. Given genome-led predictions of facultative anaerobic ATP metabolism, as well as extensive use of receptors and signaling pathways classically associated with animal biology, we also considered the possibility of a hypoxia-inducible factor (HIF)-dependent system for oxygen sensing, similar to that seen across the animal kingdom, including the simple animal *Trichoplax adhaerens *[69,70]. However, despite conservation of a Skp1/HIFα-related prolyl hydroxylase in *Ac*, we found no genes encoding proteins with the typical domain architecture of animal HIFα or HIFβ. Currently, therefore, HIF-dependent oxygen sensing remains restricted to metazoan lineages.

*Ac *also retains biosynthetic pathways involved in anabolic metabolism that are absent in *Dd *(for example, the shikimic acid pathway and a classic type I pathway for fatty acid biosynthesis; Table S7.1 in Additional file 1), although investment in extensive polyketide biosynthesis [71] is not evident. An autophagy pathway, as defined by genetic studies of yeast, *Dd *and other organisms [72], is present in *Ac *with little paralogue expansion or loss of known autophagy-related (ATG) genes evident (Figure 7.2 in Additional file 1) and likely contributes to both intracellular re-modeling in response to environmental cues and the interaction with phagocytosed microbes.

### Transcription factors

*Ac *shares a broadly comparable repertoire of transcription factors with *Dd *excepting a number of lineage-specific expansions (Table S8.1 in Additional file 1). *Ac *encodes 22 zinc cluster transcription factors compared to the 3 in *Dd *(Figure S8.2.1 in Additional file 1) [73]. It has almost double the number of predicted homeobox genes (25) compared to the 13 in *Dd *[74]. Two are of the MEIS and PBC class respectively, with an expansion in a homologue of Wariai, a regulator of anterior-posterior patterning in *Dictyostelium *[75] comprising most of the additional members (Figure S8.3.2 in Additional file 1). Strikingly, we also identified 22 Regulatory factor × (*RFX*) genes, the first identified in an Amoebozoan [76]. The *Ac RFX *repertoire is the earliest branching yet identified and forms an out-group to other known *RFX *genes (Section 8 of Additional file 1). *Ac *has been proposed to affect plant root branching in the rhizosphere via its effects on auxin balance in plants [77]. It encodes a number of genes involved in auxin biosynthesis as well as those involved in free auxin (indole-3-acetic acid (IAA)) de-activation via formation of IAA conjugates (Table S9.1 in Additional file 1). These data suggest that *Ac *plays a role in altering the level of IAA in the rhizosphere through a strategy of alternative biosynthesis and sequestration. *Ac *may also respond transcriptionally to auxin as it encodes a member of the calmodulin-binding transcription activator (CAMTA) family (Figure S8.4.1 in Additional file 1), which in plants co-ordinate stress responses via effects on auxin signaling [78,79].

## Conclusions

Comparative genomics of the Amoebozoa has until now been restricted to comparisons between the multicellular dictyostelids and the obligate parasite *Eh *[80,81]. *Ac*, while sharing many of their features, enriches the repertoire of amoebozoan genomes in a number of important areas, including signaling and pattern recognition. LGT has significantly contributed to both the genome and transcriptome of *Ac *whose accessory genome shares unexpected similarities with a phylogenetically distant amoeba. The presence of prokaryotic TEs in *Ac *illustrates its role in the evolution of some of the earth's most unusual organisms [82] as well a number of important human pathogens [7,8][83].

*Ac *has adopted bacterial-like adhesion proteins to facilitate adherence to biofilms and H-NOX based nitric oxide signaling which likely aids in their dispersal [84]. Overall the adaptive value conferred by LGT is highlighted by the expression of the large majority in *Ac *across multiple conditions, which points to their adoption into novel transcriptional networks. Given the feeding behavior of *Ac*, it seems plausible that eukaryote-to-eukaryote gene transfers may also have provided adaptive benefits [23]. Increased sampling will be necessary to establish the extent to which such gene transfers made their way into the *Ac *genome and whether 'you are what you eat' equally applies to a diet of eukaryotes [23].

*Ac *participates in a myriad of as yet unexplored interactions, as reflected in the diversity of genes devoted to sensory perception and signal transduction of extracellular stimuli. *Ac*'s survival in the rhizosphere is likely contingent on interactions not only with other microbes but also on a cross-talk with plant roots through manipulation of the levels of the plant hormone auxin. LGT may also have provided *Ac *with some of its recognition and environmental sensing components. An interesting parallel is the planktonic protozoan *Oxyrrhis marina*, which utilizes both MBP and LGT-derived sensory rhodopsins, to enable selective feeding behavior through prey detection and biorecognition [85]. We predict that host response of *Ac *to pathogens and symbionts is likely modulated via a diversity of predicted PRRs that act in an analogous manner to effectors of innate immunity in higher organisms. Given the close association of *Ac *with a number of important intracellular pathogens, it will be interesting to determine which host-pathogen interactions can trace their origins to encounters with primitive cells such as *Ac*.

*Ac *shares protein family expansions in signal transduction with other Amoebozoa while introducing new components based on novel domain architectures (nucleotidyl cyclases) [86]. The presence of the complete pTyr signaling toolkit especially when contrasted with its absence in the multicellular dictyostelids is a remarkable finding of the *Ac *genome analysis. However the role of tyrosine kinase signaling in both amoebozoan and mammalian phagocytosis [87-89] indicates that it likely represents an ancestral function. The most parsimonious interpretation predicts the supplanting of functions originally carried out by tyrosine kinases by other kinases in the Amoebozoa. This emphasizes the importance of representative sampling and in its absence the inherent difficulties in re-constructing ancestral signaling capacities.

Transcriptional response networks can be re-programmed either through expansion of transcription factors or their target genes [90]. *Ac *and *Dd *share a conserved core of transcription factors with any differences between them largely accounted for by lineage-specific amplifications. These may result in sub- or neo-functionalization contributing to the adaptive radiation of Acanthamoebae into new ecological niches.

Comparison of *Ac *with *Dd *highlights a broadly similar apparatus for environmental sensing and cell-cell communication and implies that the molecular elements underpinning the transition to a multicellular lifestyle may be widespread. Such transitions would likely have involved co-option of ancestral functions into multicellular programs and have occurred multiple times. Our analysis suggests that many signal processing and regulatory modules of higher animals and plants likely have deep origins and are balanced with subsequent losses in certain lineages including tyrosine kinases in fungi, plants and many protists.

The availability of an *Ac *genome offers the first opportunity to initiate functional genomics in this important constituent of a variety of ecosystems and should foster a better understanding of the amoebic lifestyle. Utilizing the genome as a basis for unraveling the molecular interactions between *Ac *and a variety of human pathogens will provide a platform for understanding the contributions of environmental hosts to the evolution of virulence.

## Materials and methods

### DNA isolation

*Ac *strain Neff (ATCC 30010) was grown at 30°C with moderate shaking to an OD_550 _of approximately 1.0. Total nucleic acid preparations were depleted of mitochondrial DNA contamination via differential centrifugation of cell extracts [91]. High molecular weight DNA was extracted from nuclear pellets either on Cesium chloride-Hoechst 33258 dye gradients as per [92] or by utilizing the Qiagen Genomic-tip 20/G kit (Qiagen, Hilden, Germany).

### Genomic DNA library preparation and sequencing

All genomic DNA libraries were generated according to the Illumina protocol Genomic DNA Sample Prep Guide - Oligo Only Kit (1003492 A); sonication was substituted for the recommended nebulization as the method for DNA fragmentation utilising a Biorupter™ (Diagenode, Liége, Belgium). The library preparation methodology of end repair to create blunt ended fragments, addition of a 3'-A overhang for efficient adapter ligation, ligation of the adapters, and size selection of adapter ligated material was carried out using enzymes indicated in the protocol. Adapters and amplification primers were purchased from Illumina (Illumina, San Diego, CA, USA); both Single Read Adapters (FC-102-1003) and Paired End Adapters (catalogue number PE-102-1003) were used in library construction. All enzymes for library generation were purchased from New England Biolabs (Ipswitch, MA, USA). A limited 14-cycle amplification of size-selected libraries was carried out. To eliminate adapter-dimers, libraries were further sized selected on 2.5% TAE agarose gels. Purified libraries were quantified using a Qubit™ fluorometer (Invitrogen, Carlsbad, CA, USA) and a Quant-iT™ double-stranded DNA High-Sensitivity Assay Kit (Invitrogen). Clustering and sequencing of the material was carried out as per the manufacturer's instructions on the Illumina GAII platform in the UCD Conway Institute (UCD, Dublin, Ireland).

### RNA extraction and RNA.seq library preparation and sequencing

For all tested conditions (Table S1.6.1 in Additional file 1) except the infection series, RNA was extracted from a minimum of 1 × 10^6 ^cells using TRIzol® (Invitrogen/Life Technologies, Paisley, UK). For infection material the detailed protocol is published in [93]. Strand-specific RNA.seq libraries were generated from total RNA using a modified version of [94] which is detailed in [93]. Briefly, total RNA was poly(A) selected, fragmented, reverse transcribed and second strand cDNA marked with the addition of dUTP. Standard Illumina methodology was followed - end-repair, A-addition, adapter ligation and library size selection - with the exception of the use of 'home-brew 6-nucleotide indexed' adapters as per Craig *et al*. [95]. Prior to limited amplification of the libraries, the dUTP marked second strand was removed via Uracil DNA-Glycosylase (Bioline, London, UK) digestion. Final libraries were quantified using the High Sensitivity DNA Quant-iT™ assay kit and Qubit™ Fluorometer (Invitrogen/Life Technologies). All sequencing was carried out in UCD Conway Institute on an Illumina GAII as per the manufacturer's instructions.

### Sequencing and assembly

Genome assembly was carried out using a two-step process. Firstly, the Illumina reads were assembled using the Velvet [96] short read assembler to generate a series of contigs. These assembled contigs were used to generate a set of pseudo-reads 400 bp in length. These pseudo reads were then assembled in conjunction with the 454 FLX and Sanger sequences using version 2.3 of the GS De Novo Assembler using default parameters (Table S1.1.1 in Additional file 1). The assembly contained 45.1 Mb of scaffold sequence, of which 3.4 Mb (7.5%) represents gaps and 75% of the genome is contained in less than 100 scaffolds. For assembly statistics see Table S1.2.1 in Additional file 1. In order to determine the coverage of the transcriptome, we aligned our genome assembly to a publicly available EST dataset from GenBank (using the entrez query acanthamoeba EST) AND 'Acanthamoeba castellanii' [porgn:txid5755]). Of the 13,784 EST sequences downloaded, 12,975 (94%) map over 50% of their length with an average percent identity of 99.2% and 12,423 (90%) map over 70% of their length with an average percent identity of 99.26%.

### Gene structure prediction

Gene finding was carried out on the largest 384 scaffolds of the *Ac *assembly using an iterative approach by firstly generating gene models directly from RNA.seq to train a gene-finding algorithm using a genome annotation pipeline followed by manual curation. Firstly, predicted transcripts were generated using RNA.seq data from a variety of conditions (Table S1.4.1 in Additional file 1) in conjunction with the G.Mo.R-Se algorithm (Gene Modelling using RNA.seq), an approach aimed at building gene models directly from RNA.seq data [97] running with default parameters. This algorithm generated 20,681 predicted transcripts. We then used these predicted transcripts to train the genefinder SNAP [98] using the MAKER genome annotation pipeline [99,100]. MAKER is used for the annotation of prokaryotic and eukaryotic genome projects. It identifies repeats, aligns ESTs (in this case the transcripts generated by the G.Mo.R-Se algorithm) and proteins from (nr) to a genome, produces *ab-initio *gene predictions and automatically synthesizes these data into gene annotations. The 17,013 gene predictions generated by MAKER were then manually annotated using the Apollo genome annotation curation tool [101,102]. Apollo allows the deletion of gene models, the creation of gene models from annotations and the editing of gene starts, stops, and 3' and 5' splice sites. Models were manually annotated examining a variety of evidence, including expressed sequence data and matches to protein databases (Section 1 of Additional file 1). Out of a total of 113,574 exons, 32,836 are exactly covered and 64,724 are partially covered by transcripts and 7,193 genes have at least 50% of their entire lengths covered by transcript data.

### Functional annotation assignments

Functional annotation assignments were carried out using a combination of automated annotation as described previously [103] followed by manual annotation. Briefly, gene level searches were performed against protein, domain and profile databases, including JCVI in-house non-redundant protein databases, Uniref [104], Pfam [105], TIGRfam HMMs [106], Prosite [107], and InterPro [108]. After the working gene set had been assigned an informative name and a function, each name was manually curated and changed where it was felt a more accurate name could be applied. Predicted genes were classified using Gene Ontology (GO) [109]. GO assignments were attributed automatically, based on other assignments from closely related organisms using Pfam2GO, a tool that allows automatic mapping of Pfam hits to GO assignments.

### Data access

This whole genome shotgun project has been deposited at DDBJ/EMBL/GenBank under the accession AHJI00000000. The version described in this paper is the first version, AHJI01000000. The RNA.seq data are available under accessions SRA061350 and SRA061370-SRA061379.

## Abbreviations

*Ac*: *Acanthamoeba castellanii*; bp: base pair; *Dd*: *Dictyostelium discoideum*; *Ed*: *Entamoeba dispar*; *Eh*: *Entamoeba histolytica*; EST: expressed sequence tag; GO: Gene Ontology; GPCR: G-protein-coupled receptor; HIF: hypoxia-inducible factor; H-NOX: heme-nitric oxide/oxygen binding; IAA: indole-3-acetic acid; LGT: lateral gene transfer; LRR: leucine-rich repeat; MAMP: microbe-associated molecular pattern; MAPK: mitogen-activated protein kinase; MBP: mannose binding protein; *Ng*: *Naegleria gruberi*; PRR: pattern-recognition receptor; PTK: tyrosine kinase 'writer'; PTPs: tyrosine phosphatase 'eraser'; pTyr: phosphotyrosine; RFX: Regulatory factor X; SH2: Src homology 2 'reader' domain; TKL: tyrosine kinase like.

## Competing interests

The authors declare that they have no competing interests.

## Authors' contributions

Experiments were conceived and designed by MC, AJL, and BL. Analyses were carried out by all authors. Cell cultures of *A. castellanii *were grown and DNA isolated by AJL. DNA sequencing libraries were made and sequencing carried out by AJL. The manuscript was drafted by BL, with contributions from all authors. All authors read and approved the final manuscript for publication.

## Supplementary Material

Additional file 1 **Supplementary online material**. Click here for file

Additional file 2 **Supplementary material supporting the LGT analysis**. Click here for file
