# Supplementary material for: Genome of Acanthamoeba castellanii highlights extensive lateral gene transfer and early evolution of tyrosine kinase signaling
Source: Genome Biol. 2013 Feb 1;14(2):R11. doi: 10.1186/gb-2013-14-2-r11 (PMC4053784; doi:10.1186/gb-2013-14-2-r11)
Supplement: Additional file 1 — Supplementary online material. [file gb-2013-14-2-r11-S1.PDF]

Additional File 1 – Supplemental Data and Discussion

Clarke & Lohan *et al.* **Genome of *Acanthamoeba castellanii* highlights extensive lateral gene transfer and early evolution of tyrosine kinase signalling**.

Table of Contents

1. **Sequencing Strategy and Statistics** **3**
   - *1.1. Sequencing and assembly* *3*
   - *1.2. Genome assembly* *3*
   - *1.3. Paired end reads* *4*
   - *1.4. Comparison of assembly with transcript data* *4*
   - *1.5. Gene finding* *4*
   - *1.6. Transcript coverage for the predicted gene set* *5*
   - *1.7. Functional annotation assignments* *6*
2. **Genome Elements** **6**
   - *2.1. Lateral gene transfer (LGT)* *6*
   - *2.2. Introns – evidence of mechanisms of intron gains in LGTs* *7*
3. **Cell Signalling** **7**
   - *3.1. G-protein coupled receptors* *7*
   - *3.2. Sensor histidine kinase* *11*
   - *3.3. Nucleotidyl cyclases* *13*
   - *3.4. Cyclic nucleotide binding domains* *15*
   - *3.5. Cyclic nucleotide phosphodiesterases* *16*
   - *3.6. Rhodopsins* *17*
4. **Cellular Response** **18**
   - *4.1. Kinome of A. castellanii* *18*
   - *4.2. A. castellanii tyrosine kinases and tyrosine phosphatases* *20*
   - *4.3. Tyrosine loss and analysis of pTyr motifs in A. castellanii* *25*
   - *4.4. A. castellanii SH2 domain containing proteins* *26*
   - *4.5. Phosphotyrosine circuits in A. castellanii* *29*
5. **Cell Adhesion** **31**
   - *5.1. Immunoglobulin domains* *32*
6. **Microbial Recognition** **34**
   - *6.1. Mannose binding protein* *34*
   - *6.2. Bactericidal/permeability-increasing protein (BPI) and Lipopolysaccharide-binding protein (LBP)* *34*
   - *6.3. D-galactoside/L-rhamnose binding SUEL lectins* *36*
7. **Metabolism** **36**
8. **Transcription Factors** **39**
   - *8.1. RFX transcription factors – identification and analysis* 39
   - *8.2. Zinc cluster proteins* 41
   - *8.3. Homeodomain proteins* 42
   - *8.4. Calmodulin-binding transcription activator (CAMTA)* 48
9. **Auxin Related Genes** **48**
10. **Additional Methods** **49**
    - *10.1. DNA isolation* *49*
    - *10.2 Library preparation and sequencing* *49*
11. **References** **50**

Additional File 1 Figures List

**Figure S1.6.1**	Screen shot from Apollo genome annotation curation tool
**Figure S3.1.1**	G-protein coupled receptors and Gα-subunits
**Figure S3.1.2**	Neighbor joining phylogenetic tree of Frizzled-like FZ-7TM domain proteins
**Figure S3.2.1**	Sensor histidine kinases and receivers/response regulators
**Figure S3.3.1**	Nucleotidyl cyclases
**Figure S3.4.1**	Cyclic nucleotide binding domains
**Figure S3.5.1**	Cyclic nucleotide phosphodiesterases
**Figure S4.1.1**	Kinome distributions
**Figure S4.2.1**	Sequence alignment of the PTK domain of *A. castellanii*
**Figure S4.2.2**	Phylogenetic tree of PTKs
**Figure S4.2.3**	Domain composition of *A. castellanii* PTKs and PTPs
**Figure S4.3.1**	Non-pTyr proteins in *A. castellanii* are more depleted in tyrosine than identified pTyr proteins
**Figure S4.4.1**	Domain organisation of *A. castellanii* SH2 proteins
**Figure S4.4.2**	Phylogenetic tree of *A. castellanii*, *Dictyostelium* and human SH2 domains
**Figure S4.4.3**	Evolution of domain architectures, conservation, expansion and divergence
**Figure S4.5.1**	Phosphotyrosine motifs in *A. castellanii*
**Figure S5.1.1**	Multiple sequence alignment: I-set immunoglobulin domains and amoeba immunoglobulin domains
**Figure S6.2.1**	Multiple sequence alignment of BPI (PDB 1ewf) and the *A. castellanii* homologues
**Figure S7.1**	Comparative analysis of metabolic networks for anaerobic/microaerophilic ATP production in *Acanthamoeba*
**Figure S7.2**	Conservation of the macro-autophagy pathway in *A. castellanii*
**Figure S8.1.1**	Putative *A. castellanii* RFX genes alignment
**Figure S8.1.2**	*A. castellanii* RFX genes form a diverse out-group to known RFX genes
**Figure S8.2.1**	Alignment of the zinc finger in putative zinc cluster proteins in amoeba and related species
**Figure S8.3.1**	Multiple sequence alignment of homeodomain sequences
**Figure S8.3.2**	Neighbor joining phylogenetic tree of homeodomain sequences
**Figure S8.3.3**	Multiple sequences alignment of selected MEIS class homeodomain proteins
**Figure S8.3.4**	Multiple sequence alignment of selected PBC class homeodomain proteins
**Figure S8.4.1**	Domain organisation of *A. castellanii* CAMTA relative to that found in other organisms

Additional File 1 Tables List

**Table S1.1.1**	Sequencing reads used in the assembly of the *A. castellanii* genome
**Table S1.2.1**	*A. castellanii* assembly statistics
**Table S1.3.1**	Assembly statistics – paired-end read summary
**Table S1.6.1**	Multiple RNAseq conditions used for gene modelling
**Table S3.6.1**	Rhodopsins used for phylogenetic tree
**Table S4.1.1**	The kinomes of *A. castellanii* and related amoeba species
**Table S4.3.1**	GC4 content of *A. castellanii* and two *Dictyostelium* species
**Table S5.1**	Cell adhesion proteins
**Table S6.1**	Predicted pattern-recognition receptors (PRRs) in *A. castellanii* genome
**Table S7.1**	Presence or absence of classic biosynthetic pathways in *A. castellanii* and other select soil-dwelling protists
**Table S8.1**	Summary of transcription factors in both *A. castellanii* and *D. discoideum*
**Table S9.1**	Predicted auxin related genes in *A. castellanii*

1 Sequencing Strategy and Statistics

1.1 Sequencing and assembly

The sequence generated for the *Acanthamoeba castellanii* (*Ac*) genome combined data from a number of platforms. Data from previously generated paired-end Sanger sequencing of 3-5 kb sheared genomic DNA inserts [1] was downloaded from Genbank. This data was supplemented with more cost-effective pyro-sequencing generated from the 454 Roche platform and sequencing by synthesis data from the Illumina platform. Library generation and sequencing for both the Illumina and the 454/Roche systems were carried out according to the manufacturers protocols.

Sequencing platform	Insert size	Number of reads (raw)	Number of bases (raw)	Number of reads (filtered)	Number of bases (filtered)
Illumina GAII	0.2-0.4 kb	109,943,066	4.947 Gb	105,796,410	4.232 Gb
454 FLX	5-9 kb	4,154,839	1.312 Gb	6,476,450	1.168 Gb
454 FLX	-	2,536,799	0.944 Gb	2,536,735	0.943 Gb
Sanger	3-5 kb	18,330	19.11 Mb	18,330	15.13 Mb

Table S1.1.1: Sequencing reads used in the assembly of the *Ac* genome

1.2 Genome assembly

Genome assembly was carried out using a two-step process. Firstly the Illumina reads were assembled using the Velvet short read assembler to generate a series of contigs. These assembled contigs were used to generate a set of pseudo-reads of 400 base pairs (bp) in length. These pseudo reads were then assembled in conjunction with the 454 FLX and Sanger sequences using version 2.3 of the GS De Novo Assembler using default parameters (http://454.com/products/analysis-software/index.asp). The assembly contained 45.1 Mb of scaffold sequence, of which 3.4 Mb (7.5%) was gaps and 75% of the genome is contained in less than 100 scaffolds. There were 2,064 scaffolds with a scaffold N/L50 of 31/298 kb, and a contig N/L50 of 252/40.8 kb. A full summary of the assembly statistics is given in table S1.2.1. The average read depth derived from the assembly was 37.88.

Assembly statistics	Scaffolds	Contigs
Number	2,064	5,491
Total bp (including gaps)	45.4 Mb	41.9 Mb
Total bp (excluding gaps)	41.9 Mb	41.9 Mb
N/L50	31/298 kb	252/40.8 kb
Top 5 sequence lengths	2 Mb; 1.5 Mb; 1.46 Mb; 1.45 Mb; 1.18 Mb	614.8 kb; 293 kb; 241.5 kb; 235.6 kb; 233 kb
Mean sequence size	23 kb	7.6 kb
Median sequence size	1.2 kb	1.35 kb
Number of gaps in scaffolds	4,105	-
Mean gap length	820	-
Median gap length	113	-

Table S1.2.1: *Acanthamoeba castellanii* assembly statistics

1.3 Paired end reads

Overall 1.43 million paired end (PE) reads were assembled. An overall idea of the correctness of an assembly can be determined by examining how paired-end reads were assembled [2]. The GS De Novo Assembler reports how each paired-end read was assembled (mapped). Both paired-end reads may be present in the assembly, or not, or only one of the paired-end reads may have been assembled (mapped). Both, or only one, of the paired-end reads may have been identified as a repeat read. The GS De Novo Assembler identifies a paired-end read as a "True Pair" if both reads were assembled in the correct orientation and are within the expected distance of each other in the assembly. A "False Pair" refers to a paired-end read that was assembled with either an incorrect orientation or the distance between the paired-end reads is outside the expected distance. In total 1.43 million paired end reads were present in the assembly. Of the 1.43-million paired-end reads in the assembly, approximately 1.1 million (77%) were assembled in the correct orientation and are within the expected distance of each other in the assembly.

Status	Number of PE reads
Both unmapped	297,637
One mapped	268,316
Multiply mapped	352,468
False pair	334,191
True pair	1,094,756

Table S1.3.1: Assembly statistics - paired-end read summary

1.4 Comparison of the assembly with transcript data

As RNA.seq reads are extremely shot and given the level of intronization within the genome, a number of unmapped RNA.seq reads would not be expected to provide an accurate representation of the genome coverage. Therefore in order to determine an independent measure of the coverage of the transcriptome achieved by the assembly we aligned our data to a publicly available EST dataset from Genbank (using the entrez query acanthamoeba EST) AND "Acanthamoeba castellanii"[porgn:_txid5755]). Of the 13,784 EST sequences downloaded, 12,975 (94%) map over 50% of their length with an average % identity of 99.2% and 12,423 (90%) map over 70% of their length with an average percent identity of 99.26%.

1.5 Gene finding

In order to provide an accurate dataset gene finding was carried out on the largest 384 scaffolds of the *Ac* assembly using an iterative approach. Firstly gene models were generated using RNA.seq data from a variety of conditions in conjunction with the G.Mo.R-Se algorithm [3] running with default parameters. This algorithm generated 20,681 predicted transcripts. We then used these predicted transcripts to train the genefinder SNAP using the MAKER genome annotation pipeline (http://www.yandell-lab.org/software/maker.html)[4]. MAKER is used for the annotation of prokaryotic and eukaryotic genome projects. MAKER identifies repeats, aligns ESTs (in this case the transcripts generated by the G.Mo.R-Se algorithm) and proteins from (nr) to a genome, produces *ab-initio* gene predictions and automatically synthesizes these data into gene annotations having evidence-based quality values. The 17,013 gene predictions generated by MAKER were then manually annotated

using the Apollo genome annotation curation tool (apollo.berkeleybop.org/) [5]. Apollo allows the deletion of gene models, creation of gene models from annotations and the editing of gene starts, stops, 3' and 5'-RNA.seq splice sites. Models were manually annotated looking at a variety of evidence including expressed sequence data matches to protein databases.

1.6 Transcript coverage for the predicted gene set

Out of a total of 113,574 predicted exons 32,836 exons (29%) are fully covered and 64,724 (57%) are partially covered by transcript data. 7,193 (46.3%) of the predicted gene set is covered by transcript data over at least 50% of the gene length.

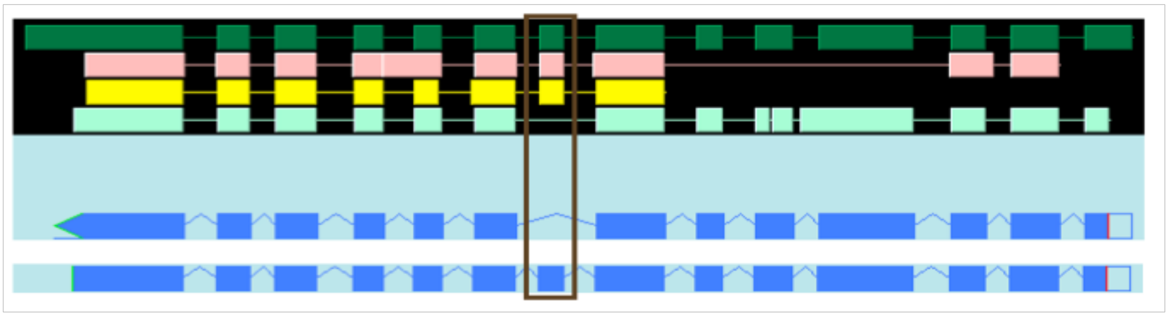

Figure S1.6.1: Screen shot from Apollo genome annotation curation tool [5]. Dark green represents aligned RNA.seq reads; Pink and yellow represent spliced protein alignments; Light blue represents SNAP genefinder predictions; Dark blue represents maker prediction prior to and following the addition of an exon to generate the final predicted gene during manual curation.

Culture Treatment	Medium	Temp	Culture: Condition & Phase
Standard Growth	Neff	ambient	Static: Mid-Log
Standard Growth	Neff	ambient	Static: Stationary
Standard Growth	Neff	30	Shaking (100 rpm): Mid-Log
Standard Growth	Neff	30	Shaking (100 rpm): Stationary
Standard Growth	PYG	30	Shaking (100 rpm): Mid-Log
Standard Growth	Neff	35	Static: Stationary
Standard Growth	Neff	37	Static: Mid-Log
Carbon Source Replacement: Sorbitol	PYS	30	Shaking (100 rpm): Mid-Log
Carbon Source Replacement: Mannitol	PYM	30	Shaking (100 rpm): Mid-Log
Carbon Source Replacement: Mannose	PYM	30	Shaking (100 rpm): Mid-Log
Mock Infection Beads 10:1: *Ac* & Beads	PYG	30	15 min post uptake
Mock Infection Beads 10:1: *Ac* & Beads	PYG	30	30 min post uptake
Mock Infection Beads 10:1: *Ac* & Beads	PYG	30	1 hr post uptake
Starvation - transfer to amoebal saline (AS)	Neff	30	0 min
Starvation - transfer to amoebal saline (AS)	AS	30	72 hr post AS transfer
Hypoxia: 1% O_2	PYG	30	Shaking (100 rpm): Mid-Log
Salt Addition: 20 mM $MgCl_2$	PYG	30	Shaking (100 rpm): Mid-Log
Salt Addition: 20 mM $CaCl_2$	PYG	30	Shaking (100 rpm): Mid-Log
Salt Addition: 20 mM $NaCl_2$	PYG	30	Shaking (100 rpm): Mid-Log
pH: pH 5.5	PYG	30	Shaking (100 rpm): Mid-Log
pH: pH 7.5	PYG	30	Shaking (100 rpm): Mid-Log
pH: pH 8	PYG	30	Shaking (100 rpm): Mid-Log
Peroxide: 0.1% H_20_2	Neff	30	Shaking (100 rpm): Mid-Log
Heat-shock: 30°C to 37°C	Neff	30	Static: Mid-Log
on *Klebsiella planticula*, 1/5 SM plates	Plates		Solid media plates

Table S1.6.1: Multiple RNA.seq conditions used for the generation of gene models. All libraries were sequenced on an Illumina GAII and strand specific libraries were generated using a modified version of [6] detailed in [7].

1.7 *Functional annotation assignments*

Functional annotation assignments were carried out using a combination of automated annotation as described previously [8] followed by manual annotation. Briefly gene level searches were performed against protein, domain and profile databases including JCVI in-house non-redundant protein databases, Uniref (http://www.ebi.ac.uk/uniref/), Pfam (http://pfam.sanger.ac.uk/), TIGRfam HMMs (http://www.jcvi.org/cgi-bin/tigrfams/index.cgi), Prosite (http://prosite.expasy.org/), and InterPro (www.ebi.ac.uk/interpro/). After the working gene set had been assigned an informative name and a function, each name was manually curated and changed where it was felt a more accurate name could be applied. Predicted genes were classified using Gene Ontology (GO) [9]. GO assignments were attributed automatically, based on other assignments from closely related organisms using Pfam2GO, a tool that allows automatic mapping of Pfam hits to GO assignments.

2 Genome Elements

2.1 *Lateral gene transfer (LGT)*

To identify cases of predicted LGT a phylogenomics approach was used consisting of an initial similarity-based screening, several filtering steps, automatic calculation and manual inspection of phylogenetic trees. The analysis was carried out using the proteomes of other amoebae, (*Naegleria gruberi* (*Ng*), *Entamoeba histolytica* (*Eh*), *Entamoeba dispar* (*Ed*), and *Dictyostelium discoideum* (*Dd*) [10-12]. The closest homologue for each protein (excluding hits to members of the same genus) from all five amoebal proteomes was extracted using SIMAP [13]. Those showing significant similarity to non-eukaryotes (E value < E^{-10}) were selected as seeds for phylogenetic tree reconstructions using PhyloGenie [14]. For paralogues, the amoebal protein showing highest similarity to non-eukaryotes was selected. The calculated maximum likelihood trees were filtered using PHAT (included in the PhyloGenie package) for nodes, which contain the amoebal protein together with bacterial, archaeal or viral proteins and no more than two proteins from other unicellular eukaryotic organisms, with bootstrap support above 75%. Phylogenetic trees were inspected manually to determine the final selection of LGT genes.

Putative LGT donors were determined using the calculated trees by identification of the closest non-eukaryotic protein to the amoebal (seed) protein based on the sum of edge distances. The taxonomic affiliation of the donors was used in order to calculate the contribution of each domain, bacterial phylum or class to the total number of LGT events. These data were then used to calculate Bray-Curtis [15] similarities and the similarity matrices were visualized as heat maps using JColorGrid [16]. Ecological information was collected for each donor using information available at the NCBI website (http://www.ncbi.nlm.nih.gov/genomes/lproks.cgi) and literature searches. Data was collected for oxygen requirements (aerobic, facultative and anaerobic) and predicted habitat (host associated or environmental). LGT candidates were assigned to Clusters of Orthologus Groups of proteins (COGs) using SIMAP and their functional description [17] was extracted from the eggNog database [18]. The majority of LGT candidates were poorly characterized, followed by the categories “energy production and conversion”, “carbohydrate transport and metabolism”, and “amino acid transport and metabolism”. Other functional categories such as “information storage and processing” and “cellular processes and signalling” were much less abundant. Notably, this pattern was highly similar across all amoeba genomes analyzed, implying that metabolism related genes are more easily integrated after LGT events than

genes involved in other biological roles, and suggesting that the amoebae irrespective of their ecology and life style acquired similar functions.

2.2 Introns - Evidence of mechanisms of intron gain in LGTs

We focused on mechanisms of intron gain in predicted laterally transferred genes as these would have been intronless at the time of transfer from prokaryotes. We sought evidence for eight proposed mechanisms of intron gain. Firstly whether the LGT introns arose by transposition of existing introns [19]. Secondly we attempted to test whether introns are transferred to new sites in a gene by non-allelic homologous recombination with a paralogous gene. Thirdly, we tested whether LGT introns arose from transposable elements within the genome [20]. Fourthly to test whether new introns evolve from the excess sequence created by short tandem intragenic repeats [21] we compared LGT introns against the DNA and mRNA sequences of their resident genes. Fifth, we found no evidence that new LGT introns arose from so-called type II self-splicing bacterial introns [22]. Sixthly, whether introns arise from insertion of sequences transferred to the nucleus [23] - we compared the sequence of introns against the *Ac* mitochondrial genome with a cutoff of e^{-10}; with no significant matches. Seventh, we sought to test the 'intronization' hypothesis, in which an exon acquires splicing boundaries within the interior of the exonic sequence, yielding a new intron [24, 25]. Finally, we tested the staggered double-strand break repair hypothesis, wherein new introns arise from quasi-random sequence added in the course of repairing double-strand breaks however we were not able to recover any clear evidence for the staggered double-strand break repair hypothesis.

3 Cell Signalling

3.1 G-Protein coupled receptors

G-protein coupled receptors (GPCRs) comprise a family of proteins with seven transmembrane helices that represent the most abundant sensors for extracellular stimuli in metazoa and are also present in other eukaryotes. The currently known GPCRs were subdivided into six families [26, 27]. Query of the *Ac* functional domain inventory genome with the often-overlapping PFAM definitions of the different classes of GPCRs yielded a total of 35 proteins. We identified seventeen *Ac* proteins belonging to family 6, the frizzled/smoothened type GPCRs, with fifteen proteins also harbouring the cysteine-rich extracellular fz domain that binds the ligand (Figure S3.1.1A). Comparison of the *Ac* proteins to Dictyostelid animal frizzled and smoothened proteins revealed that they represent a separate expansion within *Ac*, suggesting expansion from a single ancestor (Figure S3.1.2). We were not able to identify homologs of the wingless/wnt or hedgehog proteins in *Ac*. The genome also contained eight family 1 proteins, of which three conformed most to the Git3 (glucose receptor regulating gpa2) receptors, and bore greatest similarity to a fungal Git3. Two receptors conformed best to the rhodopsin domain, and were most similar to a Dictyostelid (Dicty) GPCR. As a group the three receptors were more related to human transmembrane protein 145 than to rhodopsin, and are therefore unlikely to sense light [28]. The third set of family_1 GPCRs contained 3 proteins with Lung_7TM_R domain; they were most similar to a Dicty and plant GPCR. Five *Ac* proteins represent the family 2 secretin-like GPCRs, but there are no representatives of the family 3 metabotropic glutamate-like GPCRs or the family 4 fungal pheromone receptors. Family 6, the receptors with Dicty cAMP-receptor domains are represented by six proteins. The number of GPCRs in *Ac* is only slightly

lower than in the Dictyostelids *Dictyostelium fasciculatum* (*Df*) with 38 and *Polysphondylium pallidum* (*Pp*) with 41 GPCRs. The only striking difference is the complete lack of family 3 GPCRs in *Ac*, which are well represented in all Dictyostelid genomes [28].

We detected 5 complete and 3 truncated genes for G-protein α-subunits in the *Ac* genome (Figure S3.1.1C). Two of the truncated fragments could represent the N- and C-termini of the same protein. This protein is most similar to *Dictyostelium purpureum* (*Dp*) Gα1, while two other proteins in the same clade are most similar to *Pp* Gα5. The other four Gα subunits are more similar to a choanoflagellate and a tunicate Gα subunit. The most common target proteins that are activated by heterotrimeric G-proteins are the twelve transmembrane adenylate cyclases and phospholipase C. We could not detect the former, but one phospholipase C gene is present in *Ac* (ACA1_374100).

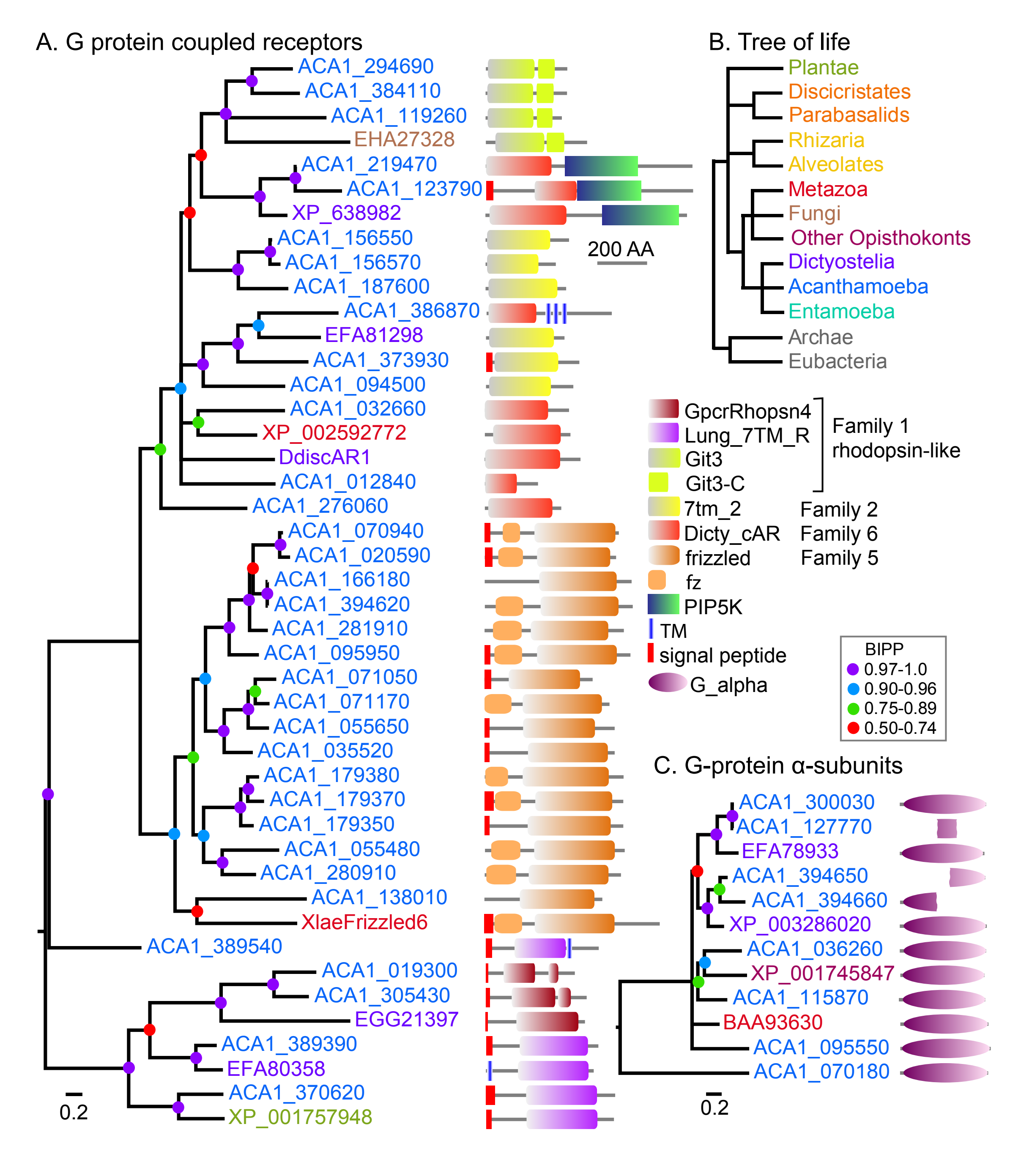

Figure S3.1.1: G-protein coupled receptors and Gα-subunits. (A) *GPCRs.* To identify G-protein coupled receptors in the *Ac* genome, the inventory of *Ac* proteins annotated with PFAM domains were queried with the domain identifiers for the different GPCR families which are PFAM domains PF10192 (GpcrRhopsn4), PF11710 (Git_3) and PF06814 (Lung7TMR) for family 1 GPCRs, PF00002 (7tm_2) for family 2, PF00003 (metabotropic glutamate) for family 3, PF02076/PF02116 (fungal pheromone receptors) for family 4, PF01534 (frizzled) for family 5 and PF05462 (Dicty_cAR) for family 6 [29]. The sequences were aligned using ClustalW [30] and a preliminary phylogeny was constructed by Bayesian inference [31]. Sequences closest to the root of each clade were used to query all non-redundant protein sequences in Genbank by BlastP. The closest hits were incorporated in the protein alignment and a final phylogeny was constructed by Bayesian inference, using a mixed amino-acid model with rate variation between sites estimated by a gamma distribution. Analyses were run for 1 million generations or until the standard deviation of the split frequencies had fallen below 0.01. Coloured dots mark the posterior probabilities of the interior nodes. Locus tags, Genbank Ids or Gene names at the tips of branches are colour coded to represent their host's position in the tree of life (panel B) and are decorated with the domain architecture of the proteins as determined with SMART [32]. The definitions of the domains for the different GPCR families overlap considerably and the domain with the lowest E-value was selected for

presentation. Genbank ids: DdiscAR1: XP_644603; XlaeFrizzled6: NP_001088182. (B): *Tree of life.* Schematic representation of the major domains of life. (C): *Gα-subunits*. The *Ac* protein inventory was queried with PFAM identifier PF00503 for the Gα- subunit of heterotrimeric G-proteins yielding 5 complete and 6 partial proteins, which were aligned and matched with their closest homologs in other organisms in a phylogenetic tree as described above.

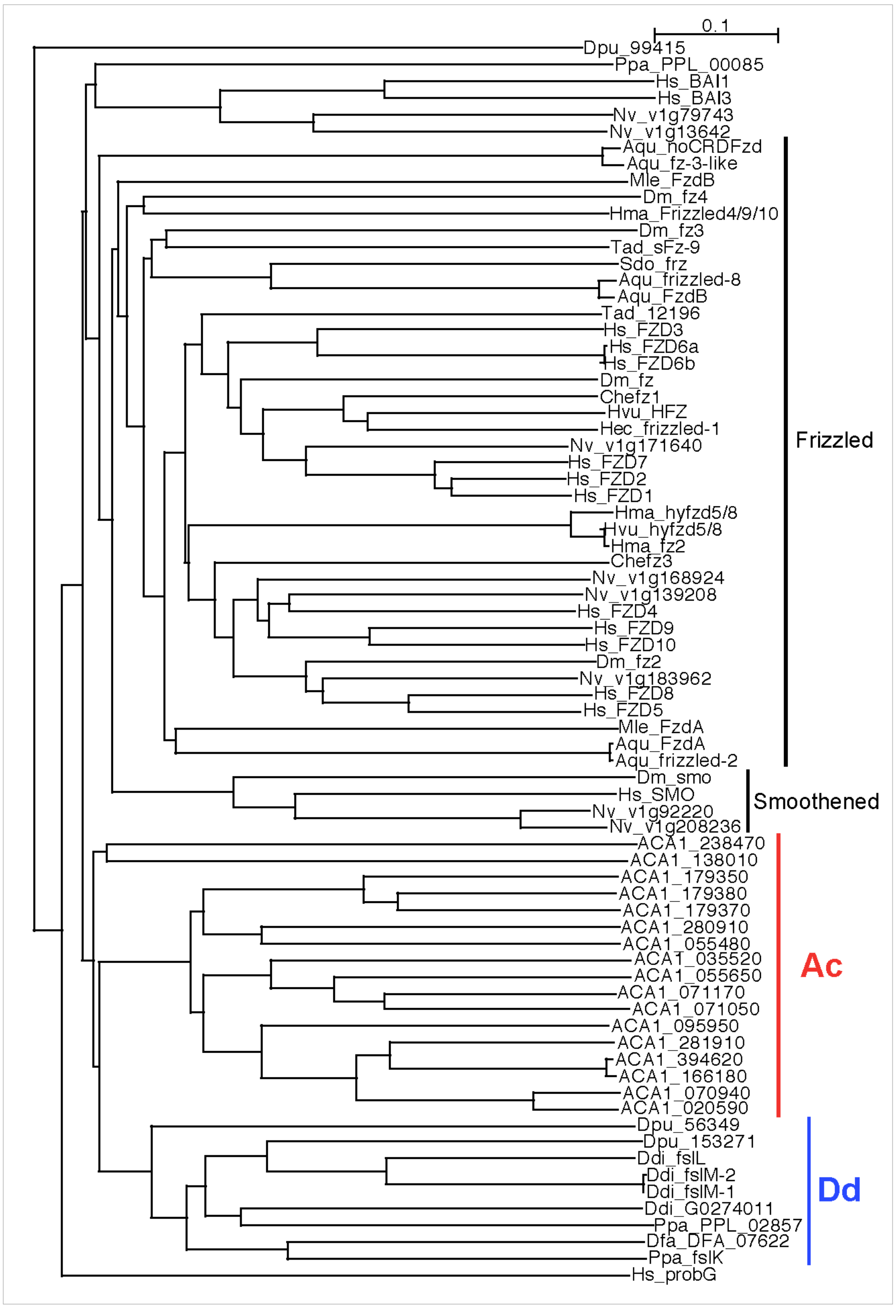

Figure S3.1.2: Neighbor joining phylogenetic tree of Frizzled-like FZ-7TM domain proteins. Frizzled and Smoothed clades as well as *Ac* and Dictyostelid clades are indicated. Sequences were analyzed as previously described [33-35]. MSAs were constructed using MUSCLE [36] and neighbor joining was carried out with ClustalX [37]. Species codes: Hs, human; Nv, *Nematostella vectensis*; Che, *Clytia hemisphaerica*; Hec, *Hydractinia echinata*; Tad, *Trichoplax adhaerens*; Hvu, *Hydra vulgaris*; Hma, *Hydra magnipapillata*; Aqu, *Amphimedon queenslandica*; Mle, *Mnemiopsis leidyi*; Sdo, *Suberites domuncula*; Ppa, *Polysphondylium pallidum*; Ddi, *Dictyostelium discoideum*; Dfa, *Dictyostelium fasciculatum*; Dpu, *Dictyostelium purpureum*; Dm, *Drosophila melanogaster*.

3.2 Sensor histidine kinases

The sensor histidine kinases (SHKs) represent another group of putative receptors for extracellular signals. They are very abundant in prokaryotes, where they can exist in a variety of functional domain configurations. Many eukaryote lineages, except the metazoa, also have sensor histidine kinases, which usually consist of at least a sensor domain, a histidine kinase/phosphatase (HATPase C) domain, an autophosphorylation (HisKA) domain and one or more receiver/response regulator domain [38]. A query of all *Ac* proteins with the PFAM identifiers of the H-ATPase C and HisKA yielded 48 putative SHKs of which 17 harboured transmembrane domains. This is a substantial number for eukaryotes. The related Dictyostelia have only sixteen sensor histidine kinases, each, while fungi and plants can contain up to 19 or 15 proteins per species [39]. However, the unrelated Vahlkampfid amoeba *Naegleria* also contains 32 sensor histidine kinases [40], indicating that this type of sensing is used extensively in protists. After construction of a pilot phylogeny of the *Ac* SHKs, the closest homologues to individual clades were identified by a BLASTP search. Surprisingly the hits were mostly prokaryotic histidine kinases with only two Dictyostelid genes and a plant gene. A complete phylogeny that also contained the outgroup sequences was constructed next (Figure S3.2.1). Apart from the HATPase-C, HisKA and receiver domain, many proteins have additional domains, such as the GAF, PAS and HAMP domains that are also found in prokaryote SHKs. The GAF and PAS domain with its associated PAC-fold are sensors for small molecules. Additionally there is one *Ac* SHK with a bacteriorhodopsin domain and three SHKs with a ser/thr or ser/thr/tyr protein kinase domain. The bacteriorhodopsin domain shares greatest similarity with a bacteriorhodopsin domain in the green algae *Chlamydomonas* that is also located at the N-terminus of an SHK [41]. One clade that contains mainly proteins with a duplicated set of SHK core domains shows greatest similarity *with Dd* DhkD. DhkD is similarly duplicated and, like the *Ac* proteins, has additional PAS/PAC domains. The duplicate SHK core domains form separate clades, indicating that the duplication occurred before *Ac* and Dictyostelids diverged.

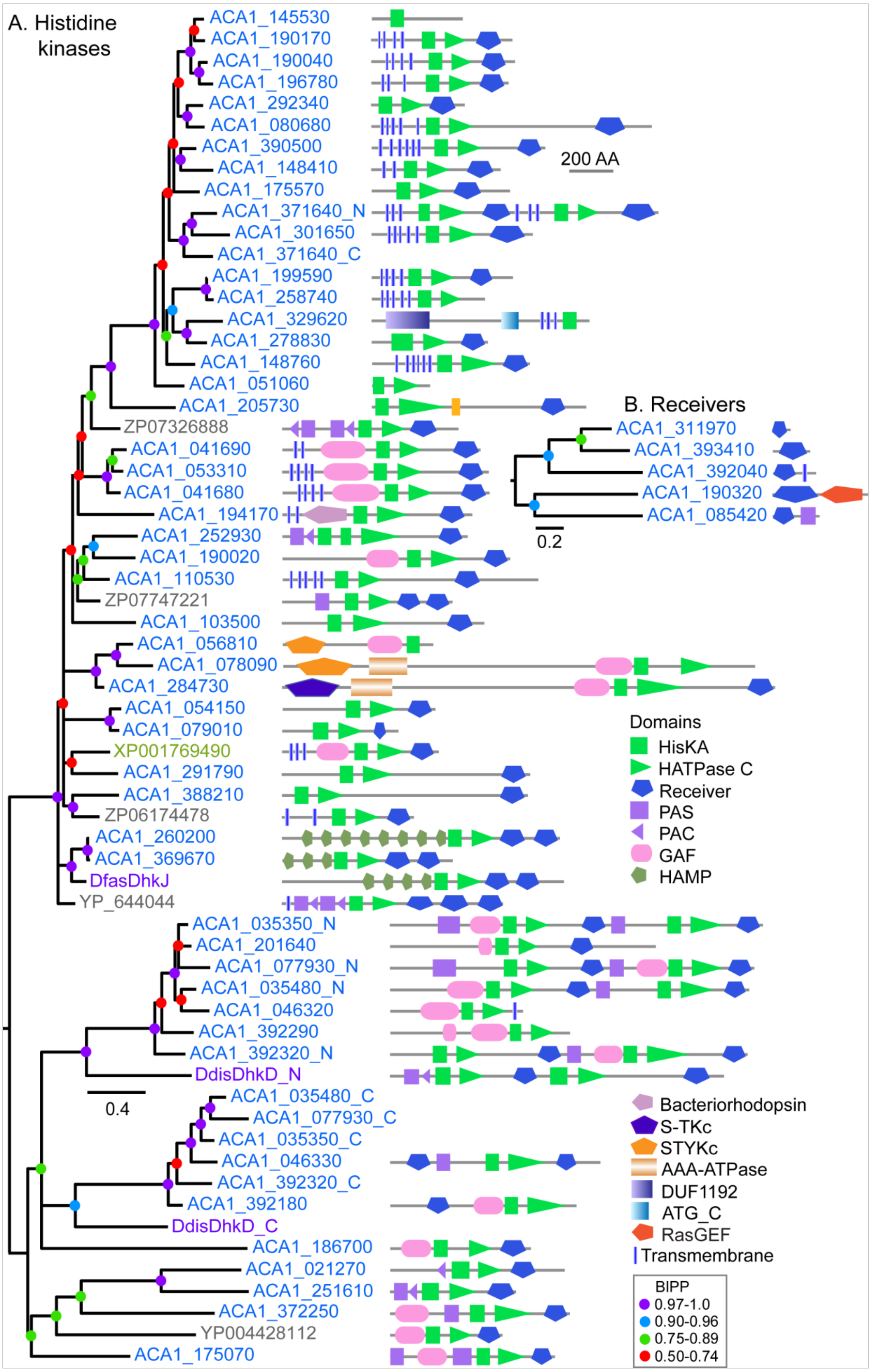

Figure S3.2.1: Sensor histidine kinases and receivers/response regulators. (A): *Sensor histidine kinases.* Sensor histidine kinases were identified by the combined presence of the PFAM PF00512 HisKA autophosphorylation domain and the PF02518 HATPase_C domain. The combined HisKA and HATPase_C sequences were used for phylogenetic inference as described in the legend to Figure S3.1.1. After construction of a guide three, the closest homologs of representative sequences for each of the major clades were identified by BLASTP search of

Genbank, and a final phylogeny was constructed including these sequences. All sequences are shown with their domain architectures. Sequence identifiers are colour coded to indicate source as shown in Figure. Proteins with two sets of HisKA/HATPase_C domains appear twice in the tree. Genbank IDs: DfasDhkJ: EGG21808; DdisDhkD: AAK50005. (B): *Receivers.* Putative targets for histidine kinase activated phosphorelay were identified as proteins that contain the receiver/response regulator domain, PF00072, but not the HisKA and/or HATPase_C domains. Only five such proteins were detected, which were individually most closely related to histidine kinases carrying the receiver domain (data not shown).

We could only identify 5 receiver proteins that were not intrinsic to SHKs (Figure S3.2.1B) and could serve to regulate the activity of the ultimate target of the sensory pathway. Three only consisted of the receiver domain, one harboured a PAS domain and one, most interestingly, a RasGEF domain. All the *Ac* receivers were more related to receivers that were intrinsic to SHKs, than to non-intrinsic receivers in Dictyostelids or other organisms (data not shown).

3.3 Nucleotidyl cyclases

Sensing of many external stimuli results in synthesis of intracellular cAMP or cGMP, which in turn activate intracellular target proteins. Eukaryotes synthesize the second messengers cAMP and cGMP using the class III nucleotidyl cyclases, which can be subdivided into four subtypes, a-d. Prokaryotes have five more unrelated catalysts for cAMP synthesis (I-VI) [42, 43]. The *Ac* genome contains a surprisingly large number of 68 adenylate or guanylate cyclases. 67 proteins are highly related to each other (Figure S3.3.1). The single outlier, ACA1_11792 shares both high sequence similarity and the same domain architecture with DdAcrA, which is essential for spore maturation [44]. Both have 6-7 transmembrane domains, a H-ATPase and two response regulator domains, N-terminal to the cyclase domain. None of the other Dictyostelid adenylate or guanylate cyclases, ACA, ACG, SGC and GCA are present in *Ac*. SGC and GCA contribute to one of four signal transduction pathways that mediate chemotaxis, while ACG regulates prespore differentiation and spore germination. ACA produces the cAMP oscillations that organize aggregation and fruiting body morphogenesis in Dictyostelids [45].

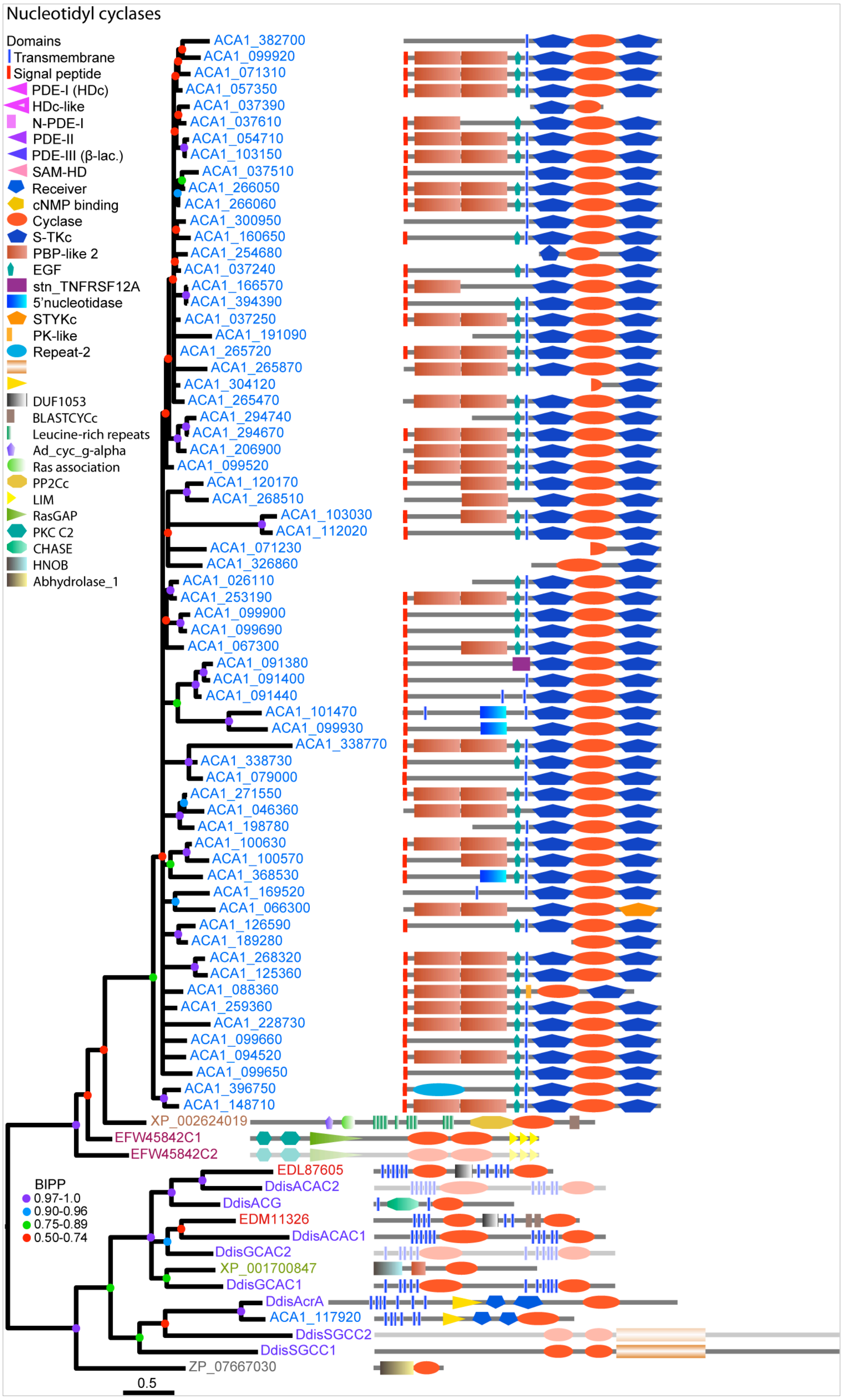

Figure S3.3.1: Nucleotidyl cyclases. Adenylate or guanylate cyclases were identified by query of the *Ac* protein library with the PFAM PF00211 identifier of the class III cyclase domain, yielding 68 proteins. After domain alignment and phylogenetic inference, 1 protein, ACA1_11792, appeared to be unique among the remaining 67 proteins, which all showed highly similar cyclase domains. The cyclase domains of the closest homolog of ACA1_11792, and of a representative protein of the other set (ACA1_14870) were used as bait in a BlastP search of Genbank to retrieve the closest homologs outside *Ac.* These proved to be the Dictyostelid AcrA enzyme for ACA1_11792 and adenylate cyclases from the fungus *Ajellomyces dermatitis* (XP_002624019) and the unicellular Opisthokont *Capsaspora owczarzaki* (EFW45842) for ACA1_14870. The *Ac* sequences and their

homologs were aligned with all *Dd* AC and GC domains and with the domains of structurally resolved ACs and GCs. The latter are *Rattus norvegicus* ACV_C1 (EDM11326), *Rattus norvegicus* ACII_C2 (EDL87605), *Chlamydomonas reinhardtii* GC (XP_001700847) and *Mycobacterium tuberculosis* AC (ZP_07667030). A phylogenetic tree was constructed by Bayesian inference and is shown with the domain architecture of the proteins and the approximate sizes (about 1600 AA for the longest proteins) the 67 related proteins and the correct relative sizes for the rest. Genbank IDs of *Dd* proteins: ACA: AAA33163; ACG: Q03101; AcrA: XP_647665; SGC: AAK92097; GCA: EAL69785.

The domain architecture of the 67 ACs is also remarkable. In almost all proteins, two serine/threonine protein kinase domains flank the cyclase domain. Those instances, where one is lacking, possibly signify faulty gene model predictions. Almost all proteins also harbour a signal peptide and a transmembrane domain that is interspersed by a large region that mostly contains one or two PBP-like-2 domains (periplasmic phosphate-binding domain) and an EGF (epidermal growth factor-like) domain. Three proteins harbour a 5′-nucleotidase domain. All three domains are usually found on the exterior face of the plasma membrane, which suggests that these *Ac* ACs are transmembrane proteins with the cyclase and kinase domains facing the cytoplasm and the other domains facing the cell’s exterior.

This orientation is similar to that of trypanosome ACs and *Dictyostelium* ACG. However, neither of those enzymes harbour protein kinase domains [42, 43]. Metazoan transmembrane guanylate cyclases do contain a kinase homology domain, but they are typical type IIIa cyclases, whereas the *Ac* enzymes are most similar to fungal type IIId cyclases. It therefore appears that the cyclases with two protein kinase domains and a large extracellular domain represent a thus far unique and extensively expanded gene family of *Ac*. It is likely that their extracellular domain acts as a receptor for external stimuli. Whether and how this receptor regulates either or both the activities of the protein kinase and AC domains remains at present a matter of conjecture.

3.4 Cyclic nucleotide binding domains

Intracellular cAMP and cGMP can be detected by the highly conserved cyclic nucleotide binding (cNMP_B) domains that are found in eukaryotes in PKG, the PKA regulatory subunit (PKAR), cyclic nucleotide regulated ion channels and in the EPAC proteins [46]. In prokaryotes, the catabolite repressor, a cAMP regulated transcription factor, harbours a cNMP_B domain. cNMP_B domains were also found in the Dictyostelid PDEs, PdeD and PdeE, and in two cGMP-regulated multidomain proteins that are involved in chemotaxis [47, 48]. Alternatively, cAMP and cGMP are detected by GAF domains [32]. However, because GAF domains can bind many other small molecules, they are not considered here.

Ac has two putative PKAR subunits, each with two cNMP_B domains (B1 and B2). The B1 and B2 domains are each individually most similar to the B1 or B2 domain of the other PKAR protein and next to the *Pp* (PpaI) PKAR B1 and B2 domains (Figure S3.4.1). Two cNMP_B domains are located C-terminally of a PDEIII domain in ACA1_134830, which is described in more detail below. Outside of *Ac*, these cNMP_B domains are most similar to their counterparts in *Df* PdeE. Within *Ac*, they are identical to the cNMP_B sequences in two otherwise featureless proteins. A weakly recognized cNMP_B domain turned out to be most similar to the conserved barrel structure of Cupin-2. In addition, there is one *Ac* cNMP_B containing protein with an additional NAD binding domain (PF02826, 2-Hacid_dh_C) and another without additional structural features. Apart from PKAR and PdeE neither of the other *Ac* cNMP_B proteins are present in the Dictyostelids, while *Ac* lacks three Dictyostelid cGMP binding cNMP_B proteins: PdeD, GbpA and GbpB. Similar to the Dictyostelia *Ac*

lacks a canonical PKG. In combination with its lack of a putative guanylate cyclase, this suggests that *Ac* does not use cGMP as a signal molecule.

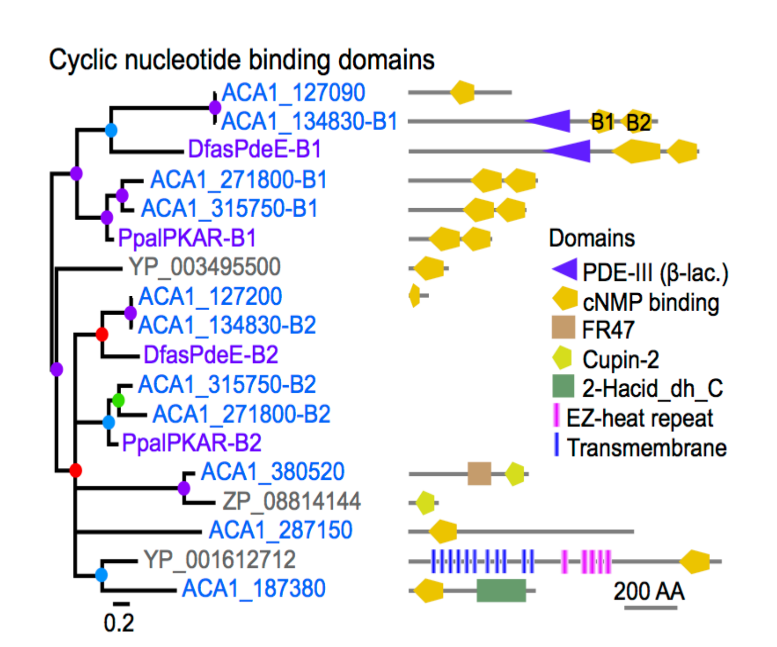

Figure S3.4.1: Cyclic nucleotide binding domains. *Ac* proteins with cyclic nucleotide binding domains (cNMP_Bs) were identified using the PFAM identifier PF00027 for this domain. The closest orthologue for each of eight detected proteins was identified by BLASTP search. A phylogenetic tree of all sequences was constructed and annotated with domain architectures as described in the legend of Figure S3.1.1. Genbank IDs: DfasPdeE: EGG20846; PpalPKAR: EFA74930.

3.5 Cyclic nucleotide phosphodiesterases

Intracellular cAMP and cGMP concentrations are rigidly controlled through hydrolysis by cyclic nucleotide phosphodiesterases (PDEs). Metazoa use different subclasses of the PDE-I type PDEs, which carry an HDc motif in their catalytic domain. Other eukaryotes, such as fungi and Dictyostelids, additionally use the low affinity PDE-II type enzymes with HSHLDH motif [49], while two Dictyostelid PDE-III enzymes (PdeD and PdeE) carry a similar HCHADHDS motif, but are structurally more related to the β-lactamase_II protein family [47]. PdeD and PdeE additionally carry two cNMP_B domains, which are most similar to the cNMP_B domain of the prokaryote CAP transcriptional regulator. When occupied, these domains allosterically activate the PDE domain. PdeD is a cGMP-stimulated cGMP PDE and PdeE a cAMP stimulated cAMP PDE.

The *Ac* genome contains eight PDE-I proteins as well as one Pde-II and one PDE-III protein (Figure S3.5.1). One of the *Ac* PDE-I proteins also harbours a SAM-HD domain and is most similar to vertebrate SAMHD1, a nuclear factor involved in immune regulation. The other five PDE-I proteins have no additional domains and are most similar to prokaryote, Dictyostelid or vertebrate PDE-I type enzymes. The single *Ac* PDE type II is most similar to a prokaryote protein, while the single PDE type III is most similar to Dictyostelid PdeE and also contains its two cNMP_B domains. With a total number of 10 putative PDEs, *Ac* surpasses *Dd* (with 8 PDEs) in its potential to hydrolyse cAMP and/or cGMP, suggesting important roles for at least one of these second messengers in signal transduction

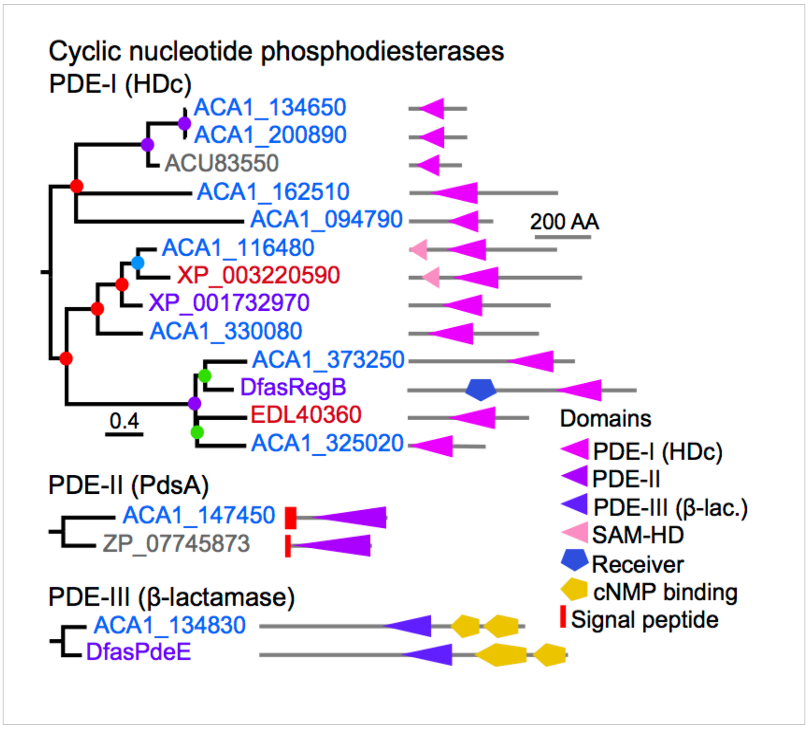

Figure S3.5.1: Cyclic nucleotide phosphodiesterases. Putative type I, II and III cyclic nucleotide phosphodiesterases were identified by query of *Ac* proteins with the PFAM identifiers PF00233, PF02112 and PF12706, respectively. The closest orthologues outside *Ac* of all retrieved proteins were identified by BLASTP search. Phylogenies of the three sets of proteins are presented as in Figure S3.1.1. Genbank IDs: DfasRegB: EGG17246; DfasPdeE: EGG20846.

3.6 Rhodopsins

Microbial rhodopsins are typically composed of seven transmembrane helices (TMH) that are able to absorb light energy for ion transport or photo-sensory functions [50]. They are involved in many cellular and physiological processes like ATP production, flagellar rotation, gene expression and a variety of signal transduction mechanisms [51]. In order to determine the relationship between the rhodopsins of *Ac* and those of other organism's rhodopsin domain sequences of 32 rhodopsins were aligned using ClustalW [52] (Table S3.6.1). The resulted alignment was subsequently manually curated with BioEdit version 7.0.9 [53] and used to construct an unrooted phylogenetic tree using the neighbor-joining method in the software MEGA version 5 [54]. Light-gated cation and anion channels were chosen from green algae (e.g. ChR2), bacteria (e.g. NpHR), cyanobacteria (ASR) and fungi (e.g. nop-1). Histidine kinase rhodopsins of green algae that are predicted to be involved in variety of signalling processes [55, 56] were also chosen. The rhodopsins of *Ac*, which belongs to the type I rhodopsin family [51], appear more closely related to the histidine kinase rhodopsins of green algae with conserved histidine kinase and response regulator domains, but lacking a C-terminal cyclase domain.

Abbreviation	Name or Type	Species	Accession Number	Reference
AC_RO1	rhodopsin-like	*Acanthamoeba castellanii*	ACA1_277930	
AC_RO2	rhodopsin-like	*Acanthamoeba castellanii*	ACA1_194170	
CrCop5	chlamyopsin-5	*Chlamydomonas reinhardtii*	AAQ16277	Kateriya et al. 2004
CrCop6	chlamyopsin-6	*Chlamydomonas reinhardtii*	XP_001698789	Kateriya et al. 2004
VcCop5	volvoxopsin-5	*Volvox carteri*	XP_002954798	Prochnik et al. 2010
VcCop6	volvoxopsin-6	*Volvox carteri*	XP_002957065	Prochnik et al. 2010
PsCop5	pleopsin-5	*Pleodorina starri*	JQ249905	Zhang et al. 2011
PsCop6	pleopsin-6	*Pleodorina starri*	JQ249906	Zhang et al. 2011
Gt2Rh	rhodopsin-2	*Guillardia theta*	ABA08438	Sineshchekov et al. 2005
ASR	Anabaena sensory rhodopsin	*Anabaena sp.*	1XIO_A	Vogeley et al. 2004
ChR1	channelrhodopsin-1	*Chlamydomonas reinhardtii*	AF385748	Nagel et al. 2002
ChR2	channelrhodopsin-2	*Chlamydomonas reinhardtii*	EF474017;AAM15777	Nagel et al. 2003
VchR1	channelrhodopsin-1	*Volvox carteri*	ABZ90900	Zhang et al. 2008
VchR2	channelrhodopsin-1	*Volvox carteri*	ABZ90903	Kianianmomeni et al. 2009
PsChR1	channelrhodopsin-1	*Pleodorina starri*	JQ249903	Zhang et al. 2011
PsChR2	channelrhodopsin-2	*Pleodorina starri*	JQ249903	Zhang et al. 2011
DchR1	channelrhodopsin-1	*Dunaliella salina*	JQ241364	Zhang et al. 2011
MChR1	channelrhodopsin-1	*Mesostigma viride*	JF922293	Govorunova et al. 2011
PgChR1	channelrhodopsin-1	*Pyramimonas gelidicola*	JQ241366	Zhang et al. 2011
AaRh	rhodopsin	*Acetabularia acetabulum*	AAY82897	Tsunoda et al. 2006
CvRh	rhodopsin	*Chlorella vulgaris*	JQ255360	Zhang et al. 2011
Gt1Rh	rhodopsin 1	*Guillardia theta*	ABA08437	Sineshchekov et al. 2005
Pop	bacteriorhodopsin-like	*Podospora anserina* S mat+	XP_001904282	Espagne et al. 2008
Mac	L. Maculans rhodopsin	*Leptosphaeria maculans*	AAG01180	Idnurm and Howlett 2001
Arch/aR-3	archaerhodopsin-3	*Halorubrum sodomense*	BAA09452	Ihara et al. 1999
cR-1	cruxrhodopsin-1	*Haloarcula argentinensis*	BAA06678	Tateno et al. 1994
BR	bacteriorhodopsin	*Halobacterium salinarum*	CAA23744	Dunn et al. 1981
NpHR	halorhodopsin	*Natronomonas pharaonis*	EF474018	Lanyi et al. 1990
cHR-5	halorhodopsin	*Haloarcula marismortui* ATCC 43049	AAV46572	Baliga et al. 2004
CsRh	rhodopsin	*Cryptomonas sp. S2*	ABA08439	Sineshchekov et al. 2005
OpsCp	rhodopsin	*Cyanophora paradoxa*	ACV05065	Frassanito et al. 2010
nop-1	rhodopsin-1	*Neurospora crassa* OR74A	XP_959421	Bieszke et al. 1999

Table S3.6.1: Rhodopsins used for phylogenetic tree – Figure 3 main text.

4 Cellular Response

4.1 Kinome of Ac

We determined the kinome of *Ac* with a sensitive and kinase group-specific HMM library [57]. *Ac* has a sophisticated kinome consisting of 377 protein kinases, the largest of any amoeba species thus far analyzed (Table S4.1.1; Figure S4.1.1).

	ePKs									aPKs				
**Species**	**AGC**	**CAMK**	**CK1**	**CMGC**	**RGC**	**STE**	**TK**	**TKL**	**Other**	**Alpha**	**PDHK**	**PIKK**	**RIO**	**TOTAL**
Ac	27	44	3	21	0	36	22	195	0	5	2	19	3	377
Ng	33	29	6	47	0	45	0	78	15	2	2	17	4	278
Eh	29	44	6	44	0	24	3	128	2	6	0	18	3	307
Dd	25	30	3	33	0	41	0	36	7	7	0	15	2	199
MIC	19	29	4	31	0	16	0	14	5	1	2	8	2	131
EctoS	35	74	6	37	0	21	5	58	8	5	7	16	3	275
Emi	34	91	9	37	0	22	1	160	4	4	5	19	2	388
Cr	18	81	5	51	0	14	4	151	4	4	4	9	2	347
LV	0	1	0	3	0	0	0	1	0	0	0	0	0	5
CLV	0	0	0	0	0	0	0	0	1	0	0	0	0	1
MarsV	0	1	0	2	0	0	0	1	0	0	0	0	0	4
Mimi	0	2	0	4	0	0	0	3	0	0	0	1	0	10
**TOTAL**	220	426	42	310	0	219	35	825	46	34	22	122	21	2322
Human	82	95	12	68	5	61	91	48	16	6	5	6	3	498
Fly	41	41	10	38	6	21	33	22	11	0	1	5	3	232
Worm	38	49	84	50	27	31	82	17	38	1	2	5	4	428
Yeast	20	36	4	25	0	14	0	0	18	0	2	5	2	126

Table S4.1.1: The kinomes of *Ac*, related amoeba species, their viruses, and those of model organisms split into the various protein kinase groups. Note: ePKs (conventional protein kinases, which comprise the majority of kinases and have a well-defined kinase catalytic domains); aPKs (atypical protein kinases, which do not possess a classic kinase catalytic domain but nevertheless display kinase catalytic activity).

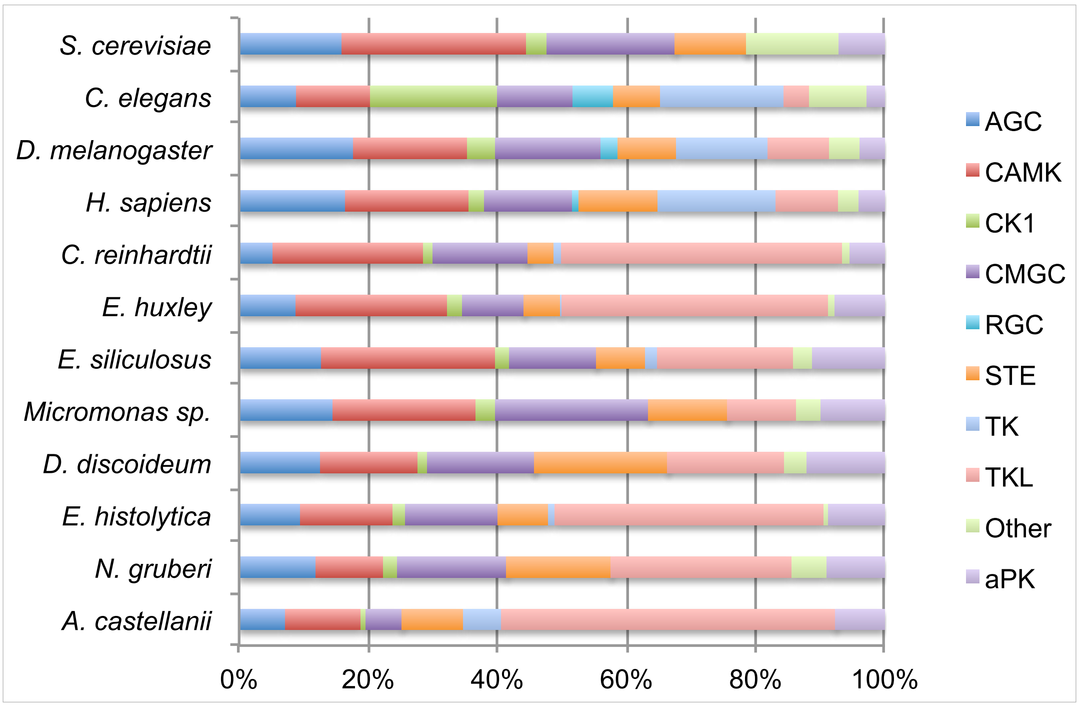

Figure S4.1.1: Kinome distributions. The distribution of the *Ac* kinome into the various protein kinase groups in comparison with related amoebas and a subset of model organisms.

The *Ac* kinome encodes kinases from all conventional (ePK) and atypical (aPK) groups. Also the *Ac* kinome includes homologs of general importance, such as cAMP-dependent protein kinase (PKA) and AMPK (and its upstream kinase LKB1, an important tumour suppressor), homologues of cell cycle control kinases (CDK2/CDK3/CDK5/CDC2), two Aurora kinases likely to be involved in the G2 to cytokinesis transition, Polo kinases likely to be required at several points during mitosis, the Rho-associated protein kinases ROCK1/2, and the multifunctional kinases CK2 and GSK3. *Ac* has an Erk7 (MAPK) homologue, a MAPKK homologue, and also MAPKKK enzymes. Lineage-specific expansions are shared by all

amoeboid species and of note is the expansion of the TKLs, with *Ac* encoding 195 TKL genes. The most remarkable feature of the *Ac* kinome, however, is the presence of 22 tyrosine kinases.

4.2 Ac tyrosine kinases and tyrosine phosphatases

Sequence alignment analysis of the tyrosine kinase domain from the 22 PTKs of *Ac* reveal a conserved "DFG" activation loop sequence, which blocks substrate binding, among all members (Figure S4.2.1). Also commonly found just downstream of the DFG sequence, within the activation loop, are tyrosine motifs that upon phosphorylation activate most metazoan tyrosine kinases such as Src, FGFR and InsR. These activation loop tyrosines are restricted to PTKs and are absent in TKLs and Ser/Thr kinases. Within the 22 PTKs in *Ac*, 9 contain tyrosine(s) within the activation loop, suggesting that these PTKs may require tyrosine phosphorylation for full activation of the kinase. Further analysis indicates the core catalytic loop motif "HRDLAARN", found in the αE to β7 loop, which is key for phosphate transfer on tyrosines is also fully conserved (Figure S4.2.1). Lastly, gatekeeper mutations within tyrosine kinases were previously identified by acquired resistance mutation to tyrosine kinase inhibitors such as imatinab [58, 59]. Mutations in the gatekeeper Thr to a hydrophobic residue results in an activated tyrosine kinase [60]. Among the PTKs in *Ac* 14 contain a Thr/Ser/Tyr at this gatekeeper position, while 5 possess a hydrophobic Val/Ala/Leu suggesting that these 5 kinases may be constitutively active, as 3 of these 5 lack Tyr motifs within the activation loop (Figure S4.2.1). Furthermore, alignment of the *Ac* tyrosine kinases with other protein kinases from *Dictyostelium* and Opisthokonts reveals three sub-classes, those that are *Dictyostelium*-tyrosine-like, another that are related to Opisthokont tyrosine kinases and a subset that bridge the boundary between tyrosine and tyrosine-like kinases, suggesting that tyrosine kinases likely evolved in a shared ancestor prior to the Amoebozoa-Opisthokont split (Figure S4.2.2).

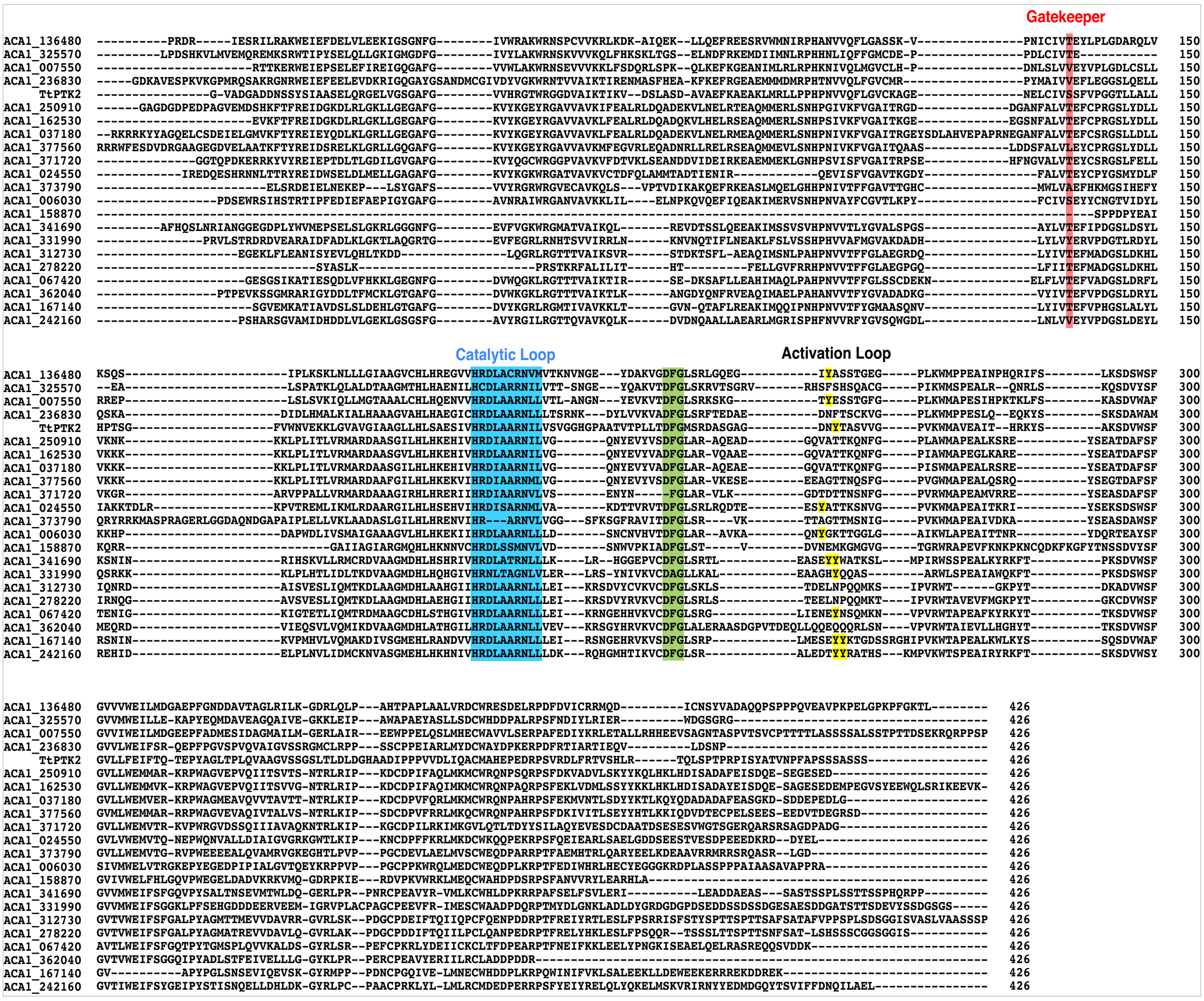

Figure S4.2.1: Sequence alignment of the PTK domain of *A. castellanii*. Clustal sequence alignment of the PTK domains of *A. castellanii* and *Thecamonas trahens* (Thecamonas). Highlighted regions are important for tyrosine kinase function. The core tyrosine kinase catalytic loop sequence "HLDLAARN" is found in both *A. castellanii* tyrosine kinases and the PTK2 kinase in *T. trahens* (blue box). The activation loop contains two critical segments, the conserved "DFG" loop found among all PTKs and the autophosphorylation loop which upon tyrosine phosphorylation activates PTKs (tyrosines are highlighted in yellow). The gatekeeper sequence is indicated in the red box.

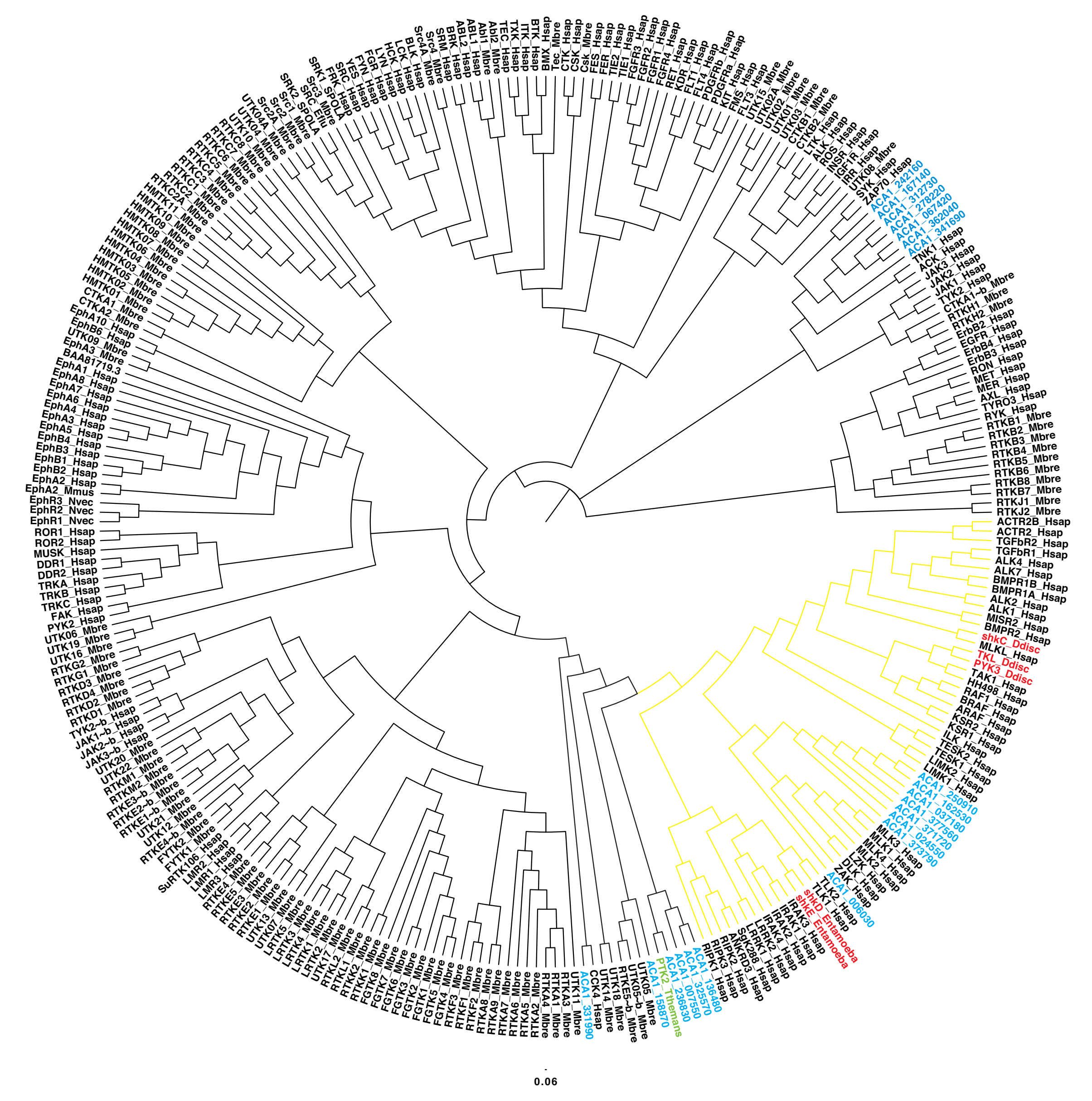

Figure S4.2.2: Phylogenetic tree of PTKs. A neighbor joining tree using the Clustal alignment of the tyrosine kinase domain or tyrosine kinase-like domains from *Ac* (ACA1_X), *T. trahens* (Thecamonas), *D. discoideum* (Ddisc), *Entamoeba histolytica* (Entamoeba), *M. brevicollis* (Mbre), *Ephydatia fluviatilis* (Eflu, freshwater sponge), *Spongilla lacustris* (SPOLA, freshwater sponge), *Nematostella vectensis* (Nvec, Nematostella), *M. musculus* (Mmus, mouse) *H. sapiens* (Hsap). Branches coloured in yellow represent kinases that are tyrosine-like kinases. *Ac* tyrosine kinases are labelled in blue text, *D. discoideum and Entamoeba histolytica* are labelled in red text*,* *T. trahens* are labelled in green text.

Examination of the domain organisation of the 22 PTKs reveal unique domain combinations distinct from those found in either *Dd* or Opisthokonts. However, several PTKs in *Ac* (contain a SAM and a tyrosine kinase domain with a similar orientation similar to *Monosiga* but distinct from metazoans (Figure S4.2.3A). Using the combination of sequence analysis and domain organisation, the PTKs of *Ac* do not represent any of the 29 known PTK families found in humans. There are two classes of PTKs, the receptor tyrosine kinases (RTKs) and cytoplasmic tyrosine kinases (CTKs). Among the PTKs of *Ac*, 7 of the 22 proteins possess a transmembrane domain (TM) indicating the use of pTyr for membrane bound signalling (Figure S4.2.3A). In comparison to metazoans, none of the *Ac* RTKs contain any extracellular domains such as the EGF, FU (furin-like) and Cys-rich domains commonly found in

metazoan RTKs suggesting the ligand induced pTyr signalling was established after the *Ac* and prior to the Opisthokont split.

PTPs are important regulators of pTyr signalling acting by catalyzing the removal of the phosphate moiety from tyrosine. Encoded within the human genome are 107 PTPs subdivided into four families [61]. The largest family are the Cys-based PTPs with 38 well-known "classical" PTPs (cPTPs) that are strictly tyrosine specific and 61 VH1-like, "dual-specific" protein phosphatases (DSPs). The domain organisation of PTPs within *Dictyostelium* and *Ac* show lack of combinations with other protein domains in all genes except one that contains a C2 domain (Figure S4.2.3B). This is distinct to *Monosiga* and other metazoans as PTPs are found linked to domains such as SH2, FERM, PDZ and others protein modules suggesting that Amoebozoa PTPs did not acquire specificity through the use of domain combinations [61-63].

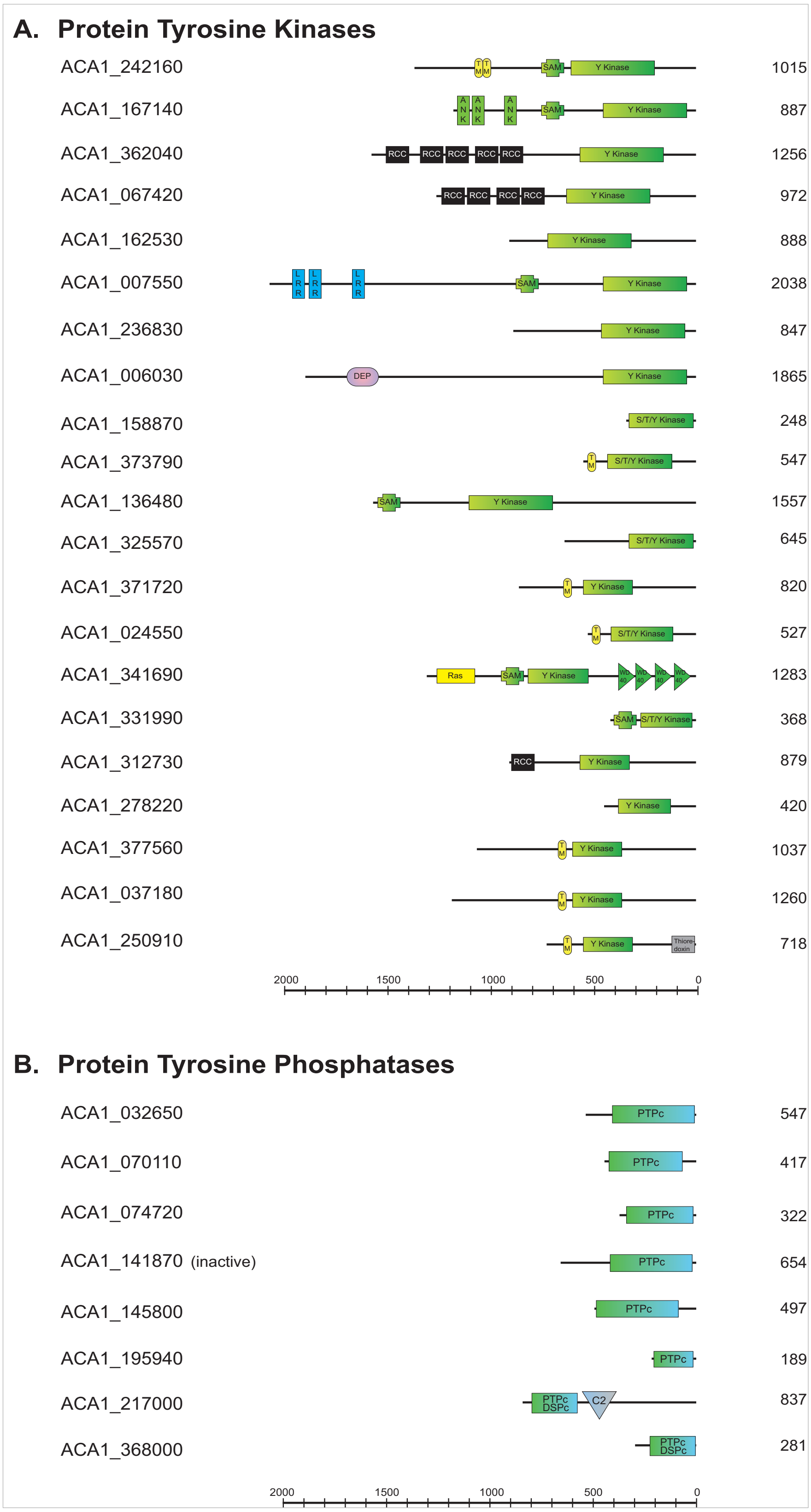

Figure S4.2.3: Domain composition of *Ac* PTKs (22) and PTPs (12). (A) Protein tyrosine kinase domain combinations. (B) Protein tyrosine phosphatase combinations. The domain architectures were predicted using the SMART (http://smart.embl-heidelberg.de/) and Pfam databases (http://pfam.sanger.ac.uk/). Abbreviations: ankyrin (ANK), transmembrane (TM), tyrosine kinase (Y kinase), Ser/Thr/Tyr kinase (S/T/Y kinase), Leucine rich repeat (LRR), sterile alpha motif (SAM), regulator of chromosome condensation (RCC), classical protein tyrosine phosphatase (PTPc), classical dual specificity phosphatases (DSPc), Dishevelled-Egl10-Pleckstrin (DEP). Amino acid lengths are indicated on the far right.

4.3 *Tyrosine loss and analysis of pTyr motifs in Ac*

Tyrosine frequency is observed to correlate negatively with the numbers of PTKs and *p*Tyr-binding protein domains (SH2 and PTB etc.) in metazoa [64]. One explanation proposed is tyrosine depletion serves to improve signalling fidelity and eliminate deleterious *p*Tyr sites. As tyrosine is encoded by two AT-rich codons, random increase in GC content in coding sequences could contribute to the observed global tyrosine depletion (GTD). To correct for this confounding effect and the evolutionary constraint imposed by the physiochemical properties of tyrosine, depletion of tyrosine is compared to depletion of phenylalanine as the two amino acids are each encoded by two AT-rich codons and are structurally identical except a phosphorylatable hydroxyl group on tyrosine. Here, we evaluate whether GTD also occurred in *Ac*, which has a more elaborate pTyr signalling than *Dp* and *Dd* (Figure S4.3.1). One-to-one orthologues of *Ac* proteins in *Dd* and *Dp* were inferred using the inParanoid algorithm with default settings [65] using all known and inferred proteins in the two *Dictyostelium* species as provided at dictyBase (www.dictybase.org/) database [66]. Similar to what was reported for human [64], we observed non-*p*Tyr proteins in *Ac* are statistically more depleted in tyrosine than *p*Tyr proteins in comparison to orthologous proteins in *Dd* and *Dp*. No statistical difference in phenylalanine depletion was observed between the two groups of proteins which is also similar to what was observed in human [67]. Similarly, coding sequences of proteins in *Dd* [68] and *Dp* [69] used to compute GC4 content were retrieved from the dictyBase database [66] (Table S4.3.1).

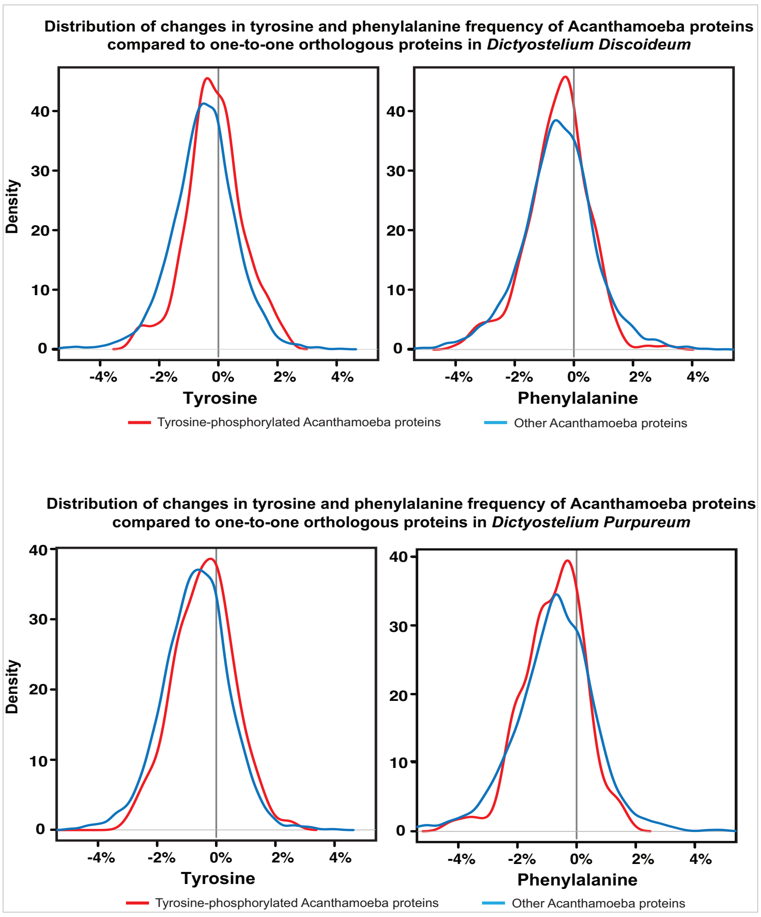

Figure S4.3.1: Non-*p*Tyr proteins in *Ac* are more depleted in tyrosine than identified *p*Tyr proteins. Overall, *Ac* proteins are significantly more depleted in tyrosine and phenylalanine compared to one-to-one orthologous proteins in *Dd* (*p*-value < 2.2e-16, Wilcoxon signed rank test, mu = 0, one-tailed) and *Dp* (*p*-value < 2.2e-16, Wilcoxon signed rank test, mu = 0, one-tailed). Tyrosine depletion, not phenylalanine depletion, is statistically more pronounced in non-*p*Tyr proteins than *p*Tyr proteins as compared to orthologous proteins in *Dd* (*p*-value =

1.8e-07, Wilcoxon rank sum test, one-tailed) and *Dp* (*p*-value = 2.6e-04, Wilcoxon rank sum test, one-tailed). As tyrosine and phenylalanine are both encoded AT-rich codons and are structurally identical except for a phosphorylatable hydroxyl group on tyrosine, the observed differentiated tyrosine depletion is likely phosphorylation linked.

**Nucleotides at 3rd codon position**	***Ac***	***Dd***	***Dp***
Adenine (A)	8.50%	50.10%	46.40%
Thymine (T)	9.40%	39.40%	37.60%
Guanine (G)	46.70%	7.90%	13.10%
Cytosine (C)	35.40%	2.60%	2.90%
GC4 Content	82.10%	10.50%	16.00%

Table S4.3.1: GC4 content of *Ac* and two *Dictyostelium* species (sequences retrieved from the dictyBase database [66]). GC4 content refers to the percentage of guanine (G) and cytosine (C) nucleotides observed at third codon position (GC3) of all four-fold degenerate codons which are GCN (alanine), CGN (arginine), GGN (glycine), CTN (leucine), CCN (proline), TCN (serine), ACN (threonine), and GTN (valine). Nucleotide variation at the third codon position of four-fold degenerate codons do not change the amino acid being encoded, hence is a readout of GC content on coding sequences that are minimized influenced by selection for amino acid changes.

4.4 Ac SH2 domain containing proteins

Several modular interaction domains have the capability to recognize a tyrosine phosphorylated ligand. These include most Src homology 2 (SH2) domains [70, 71], a subset of PTB domains [72], at least one C2 domain [73] and the Hakai pTyr-binding (HYB) domain [74]. In metazoans, the SH2 domain is the largest domain family dedicated to pTyr recognition with over 111 proteins containing at least one SH2 domain encoded in the human genome and an almost equally large number in the choanoflagellate *M. brevicollis* genome [62, 75]. The SH2 domain is the primary pTyr recognition domain of which 51 are present in *Ac* encoded within 48 proteins, 3 of which contain tandem SH2 domains (Figure S4.4.1). Alignment of the 51 SH2 domains and the 121 SH2 domains from human reveal similarity of the *Ac* SH2 domains with the Cbl and STAT families and many that resemble closely to those found in *Dictyostelium* SH2 domains (Figure S4.4.2). Sequence examination of the critical bB arginine in FLVR motif of the SH2 domain, which is critical for pTyr binding, reveal 48 of the 51 predicted SH2 domains to contain this essential motif. Domain organisation of the SH2 domains reveals unique domain combinations, with many similar to those found in *Dictyostelium*. For example *Dictyostelium* encodes 4 proteins with an SH2 domain linked to a kinase domain call Shk [76]. *Ac* has 28 copies of a Shk-like domain organisation indicating some shared and distinct functions within the repertoire of SH2 domain containing proteins between the two amoeba species (Figure S4.4.1). One interesting feature of *Ac* SH2 domain proteins are the large number of GTPase regulating domains such as RhoGAP, RhoGEF, RasGEF, and RapGAP domains which suggest small GTPase control was highly linked to pTyr signalling in *Ac*, revealing a distinct role between *Ac* and *Dictyostelium* (Figure S4.4.3B).

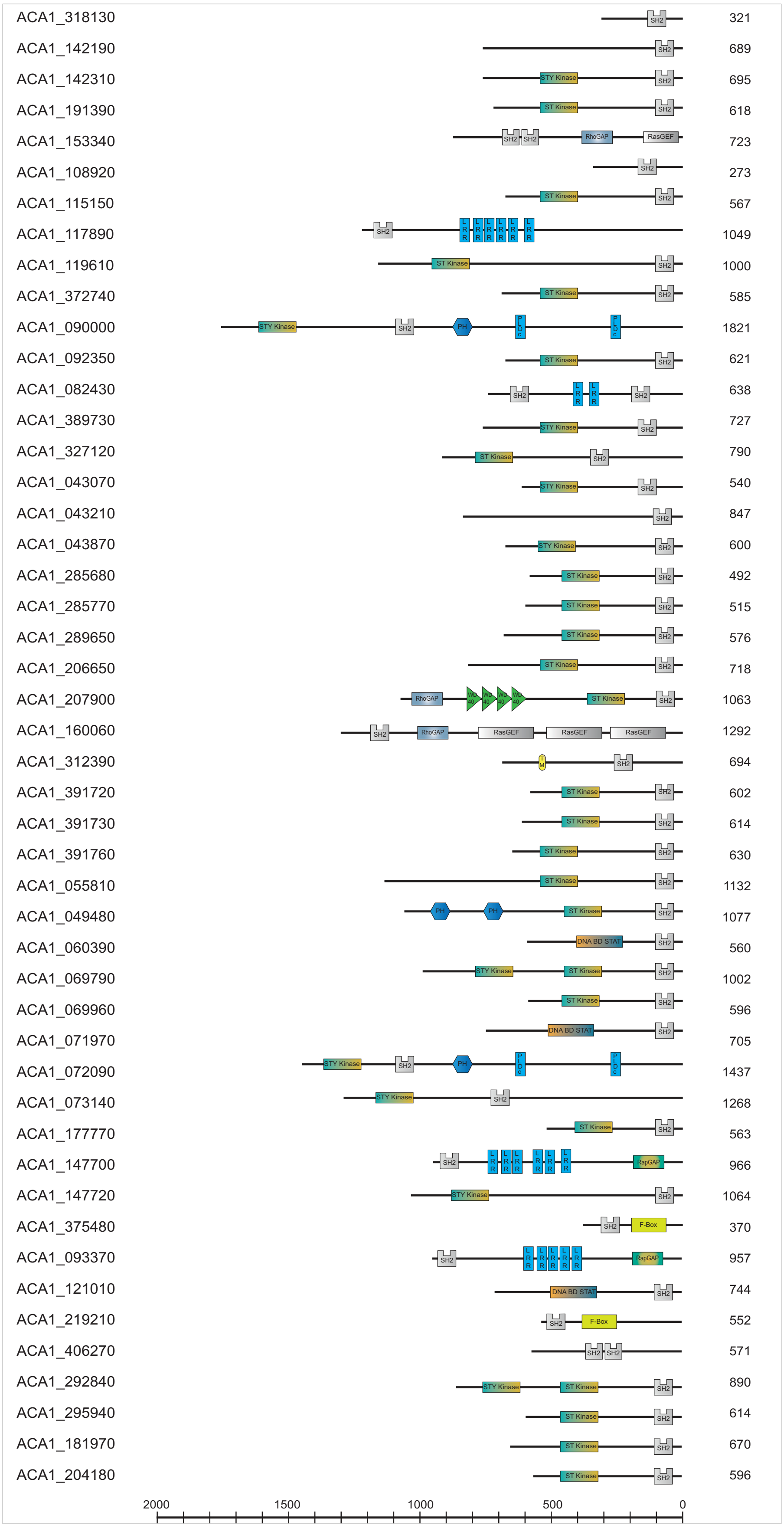

Figure S4.4.1: Domain organisation of *Ac* SH2 proteins. The domain architecture was predicted using SMART (http://smart.embl-heidelberg.de/) or Pfam (http://pfam.sanger.ac.uk/). For complete definitions of the abbreviated domains visit http://pawsonlab.mshri.on.ca.

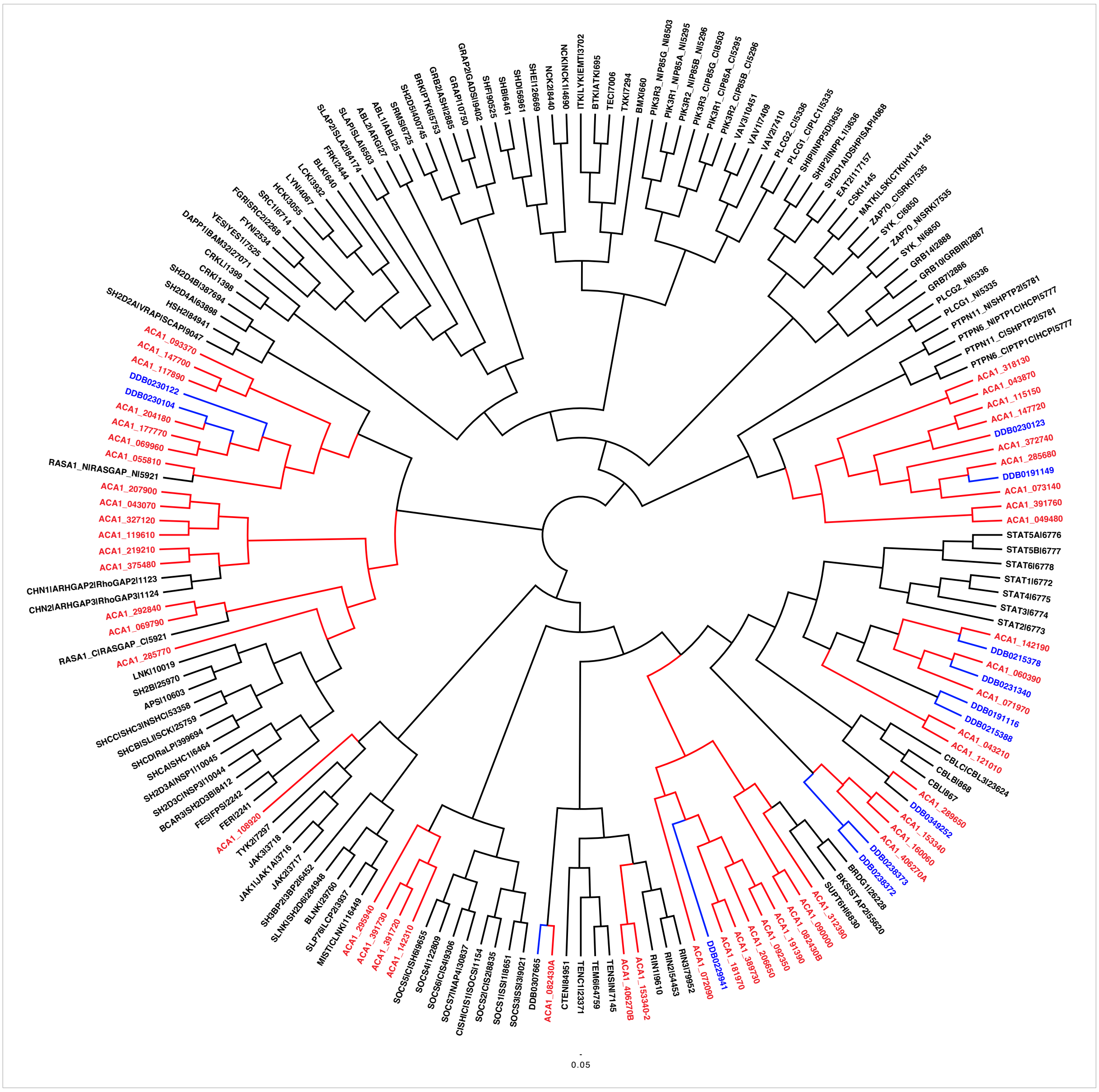

Figure S4.4.2: Phylogenetic neighbor-joining tree of *Ac*, *Dictyostelium* and human SH2 domains. Based on Clustal alignment of the SH2 domain, pairwise similarity, and conservation of key residues, the phylogenetic tree shows closely related SH2 domains between *Dd* (red lines/label), *Ac* (blue lines/label) and humans (black lines/label).

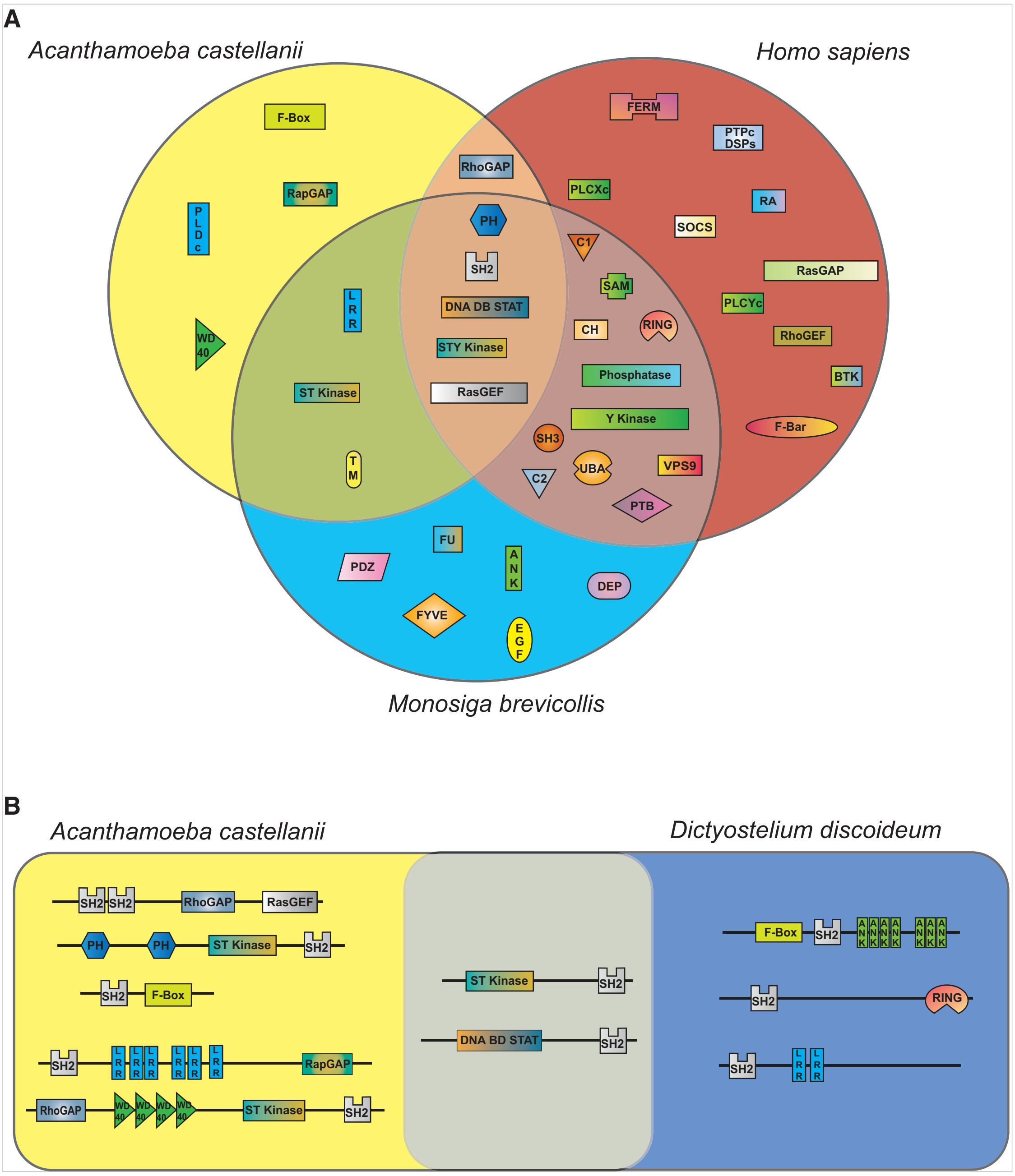

Figure S4.4.3: Evolution of domain architectures: conservation, expansion and divergence. (A) Domain organized in a Venn diagram, show overlapping and unique domains among SH2 domain containing proteins between humans *Homo sapiens*, slime mold *Dd* and the soil amoeba *Ac*. (B) Shared and distinct domain organizations of proteins containing SH2 domains are represented between *Dd* and *Ac***.** Abbreviated domains complete definitions see: http://pawsonlab.mshri.on.ca or http://www.sh2domain.org

4.5 Phosphotyrosine circuits in Ac

Tyrosine phosphorylation is an essential element of signal transduction in metazoa that is associated with the development of multicellularity and the expansion of organismal complexity. In metazoa and *Ac* tyrosine phosphorylation is a post-translation modification (PTM) that depends upon an essential triad of signalling molecules that are the PTKs that add a phosphate onto substrate tyrosine residues (the "writers"); the PTPs that remove or dephosphorylate substrates (the "erasers"); and the modular protein interaction domains that recognize the phosphorylated ligand (the "readers") and hence recruit the proteins containing

these domains to specific downstream signalling events [77]. The residues surrounding phosphorylated tyrosines are largely responsible for the specific recognition by modular pTyr recognition domains (e.g. SH2, PTB) and directing specificity for phosphorylation by tyrosine kinases. Earlier studies on pTyr sequence motifs determined the frequency of various amino acids surrounding the tyrosine residue. For example, the frequent occurrence of hydrophobic residues at positions +1 to +3 (where 0 is the position of the phosphorylated tyrosine residue and + represents residues C-terminal) has been previously described [78]. The specificity for many PTKs and a dominance of acidic, basic or hydrophobic residues adjacent to the pTyr is reported. Meanwhile, large-scale specific studies of SH2 domains reveal a large dominance of acidic and hydrophobic residues surrounding pTyr binding peptides [79-81]. To determine whether tyrosine phosphorylation in *Ac*, was utilized in a manner for SH2 domain recruitment we extracted generalized pTyr motif from the pTyr peptide dataset (Figure S4.5.1A), for example pY-x-x-I/L/V/P, pY-x-x-Q, pY-x-N-x are common pTyr motifs capable of being phosphorylated by select tyrosine kinases (Figure S4.5.1D) and recognized by a subset of SH2 domains (Figure S4.5.1C). Approximately 219 pTyr peptides contain a pY-x-x-I/L/V/P, which can be broken down in individual motifs at the +3 position for Ile, Leu, Pro, and Val indicating enrichment for pY-x-x-L and pY-x-x-P over pY-x-N motifs (Figure S4.5.1B). By comparing the PTK domains and SH2 domains from *Ac* (see sections below), these domains are closely related to metozoan domains with similar specificities. Using the extracted general motifs, we compared whether specific PTK and SH2 domains were able to recognize or phosphorylate these motifs (Figure S4.5.1C, D).

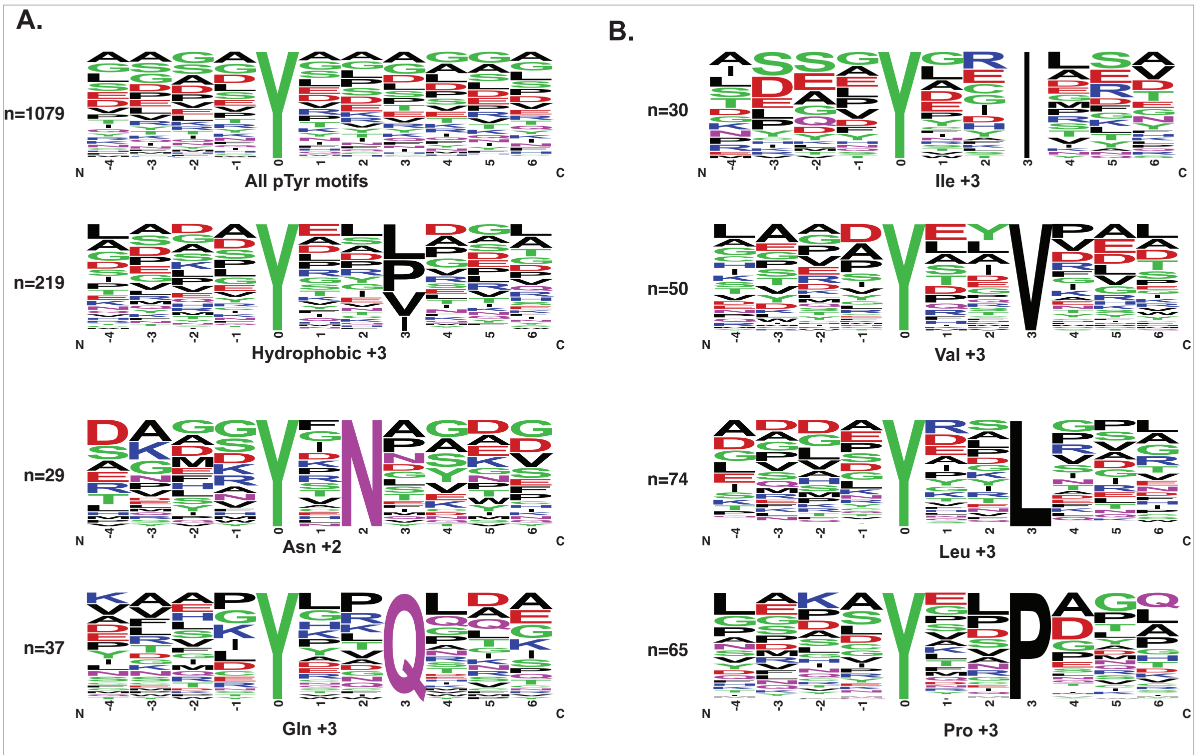

C.

Specificity	SH2 Domain
Y-x-x-P	ABL, BRK, CRK, RASA1, VAV
Y-x-N-x	GRB2, GRB7, TNS
Y-x-x-Q	STAT
Y-x-x-L	CRK, SHB, SLP76, SAP

D.

Specificity	Kinase
Y-x-x-P	ABL, SYK, TEC, EPHR
Y-x-N-x	FLT, MET
Y-x-x-Q	SRC, JAK, EGFR
Y-x-x-L	JAK, SRC

Figure S4.5.1: Phosphotyrosine motifs in *A. castellanii*. (A) Well generalized and characterized phosphotyrosine (pTyr) motifs represented as position weighted WebLogos (http://weblogo.berkeley.edu), for example pY-x-x-I/L/V/P, pY-x-x-Q, pY-x-N-x, were extracted from the 1075 pTyr peptide identified in *Ac*. (B) 219 pTyr peptides contain a pY-x-x-I/L/V/P, which can be broken down in individual motifs indicating enrichment for pY-x-x-L and pY-x-x-P over pY-x-N motifs. These common pTyr motifs can become phosphorylated by tyrosine kinases with known kinase specificity (C) and recognized by a subset of SH2 domains (D). (C-D) Specificity information for the indicated tyrosine kinases and SH2 domains were generated from mammalian studies. Highlighted in blue are SH2 or Tyrosine kinase orthologues present in *A. castellanii*.

5 Cell Adhesion

Adhesion is responsible for attachment to the substrate during movement, to particles during phagocytosis and for formation of intercellular contacts. Many genes that are important for cell-cell adhesion and kin recognition in *Dd* (e.g. csA, lagB&C) are not present in *Ac*. In comparison with *Dd* none of the Ca(2+)-dependent cell-cell adhesion molecules or the integrin-like Sib proteins used by *Dd* could be identified in *Ac*. Two homologs of the nonaspanins Phg1A, Phg1B but none of Phg1C could be identified. Two Talin homologs could be identified [82]. Three vinculin related proteins could be identified as well as a homolog of the PaxA but not Pax B. Important components of the integrin adhesion complex have not been found in *Ac* however an FG-GAP repeat protein with similarity to bacterial

integrins is present. Aardvark is part of adherens junction-like structures in the fruiting body tip of *Dd* and has additional signalling roles [83, 84]. At least four homologs of the beta-catenin-like protein Aardvark are present in *Ac* as well as a beta-catenin interacting ICAT protein homolog. *Ac* may have other cell adhesion strategies and encodes 2 transmembrane MAM domain-containing proteins. The MAM domain is thought to have an adhesive function, as it is widespread among various adhesive proteins implicated in cell-cell interactions. *Ac* also appears to utilize bacterial-like proteins with predicted roles in adhesion. Two proteins with homology to bacterial haemagluttinins and invasins with SignalP domains were identified.

Predicted funtion	Name	Domain	*A. castellanii*	*D. discoideum*
Cell-substrate adhesion; phagocytosis	Phg1A	EMP70, TM domains	ACA1_205560/ACA1_032430	DDB_G0267444
	Phg1B	EMP70, TM domains	ACA1_078790/ ACA1_272330	DDB_G0277273
	Phg1C			DDB_G0290159
Cell substrate sibA adhesion, phagocytosis	Sib SibA/B/C/D/E		Not present	
			ACA1_383350	
Cell substrate adhesion, motility	Vinculin- related, a-catenin	Vinculin domains	ACA1_073510	
	Paxillin	LIM domain LD domains		PaxB
	Paxillin	Lim domain	ACA1_324680	PaxA/Lim2
Cytohesin	Cytohesin family	Sec7, PH domains	ACA1_299340	DDB0191439
			ACA1_063240	DDB0233591
			ACA1_056690	DDB0233617
			ACA1_026220 (degenerate ILWEQ motif)	
Morphogenesis	TalB	Band 4.1 domain, ILWEQ motif, villin headpiece	Not present	DDB_G0287505
Cell cell adhesion, signaling	AarA; b-catenin	Armadillo repeats	ACA1_230590	DDB_G0288877
			ACA1_159620	
			ACA1_060370	
			ACA1_060380	
Cell Cell adhesion	CadA Cad 1/2/3		Not present	
	MAM-domain containing	MAM	ACA1_199030	
			ACA1_038820	
	bacterial haemagglutinins related	Hep_Hag domain	ACA1_117600	
	Bacterial adhesion molecule	Collagen-binding surface protein Cna, B-type domain	ACA1_379920	
	Bacterial adhesion molecule	Collagen-binding surface protein Cna, B-type domain	ACA1_138020	
	FG-GAP repeat proteins	Integrin alpha/FG-gap	ACA1_043800	
	Mannose binding protein like		ACA1_252970	
	Laminin binding protein (40S ribosomal protein)		ACA1_391390	DDB0230016

Table S5.1: Cell adhesion proteins

5.1 Immunoglobulin domains

Immunoglobulin domains are interesting because previously members of this important superfamily have been confined to metazoa [85] and the choanoflagellate *M. brevicollis* [86] with some sporadic occurrences in bacteria [87]. InterproScan searches revealed matches to immunoglobulin domains in the genome. Three proteins were identified: ACA1_210930, ACA1_290100 and ACA1_290230. Alignment of the putative Ig domains to known immunoglobulin superfamily sequences reveals that these domains are likely to belong to the I-set which has structural characteristics intermediate between the constant and variable domains of antibodies [88]. Ig domains within the I-set have been proposed to be the ancestral type [85]. The alignment of the amoebal Ig domains to the Pfam seed alignment for the I-set immunoglobulins is shown in (Figure S5.1.1).

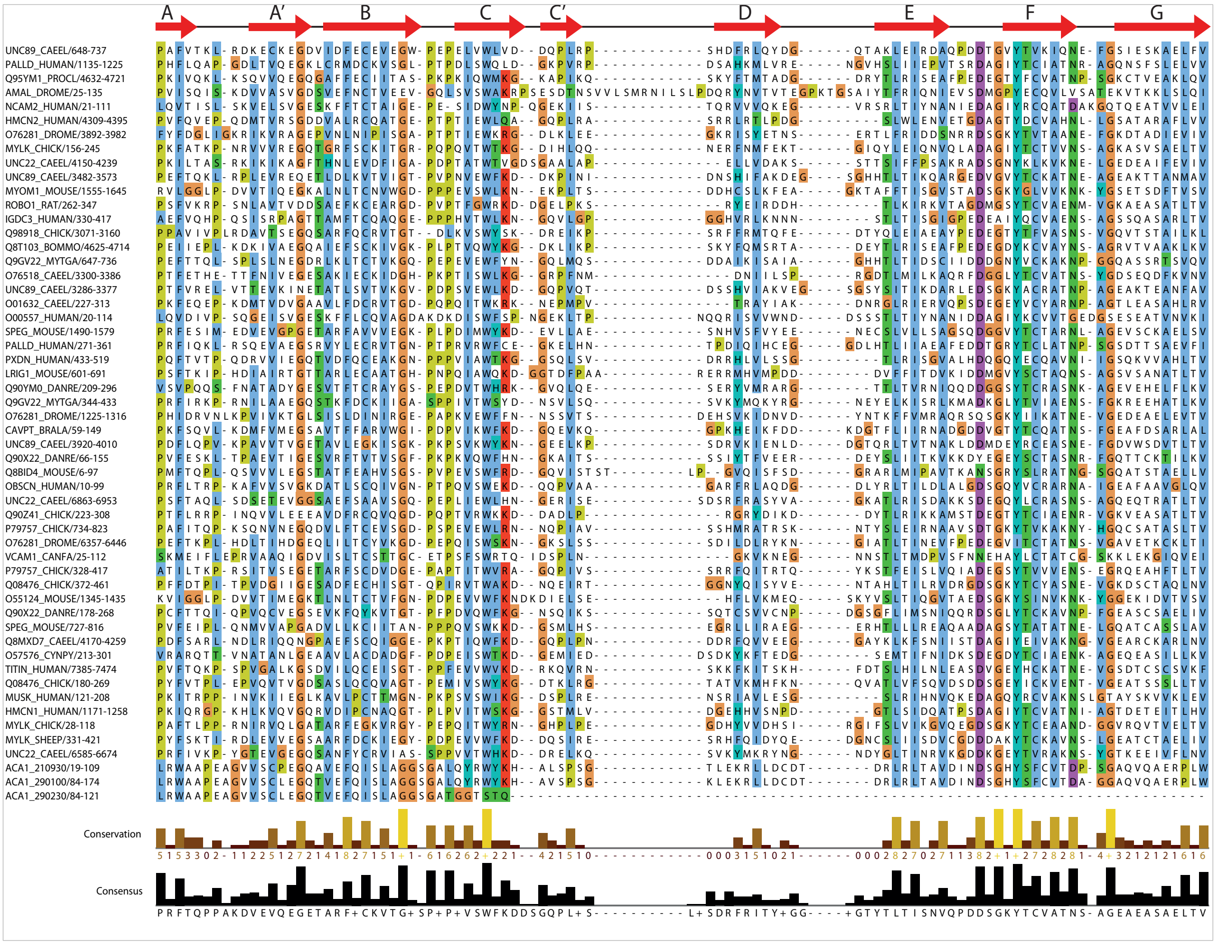

Figure S5.1.1: Multiple sequence alignment: I-set immunoglobulin domains and amoeba immunoglobulin domains. The alignment was created by manually aligning the three domains against the Pfam seed alignment for the I-set immunoglobulins using the Jalview software [89]. Colouring of the alignment is using the ClustalX colouring scheme implemented in Jalview. Secondary structure is shown using red arrows at the top of the alignment with the beta strand designations noted above.

Both ACA1_210930 and ACA1_290100 have regions that are weakly predicted to be transmembrane helices using the TMHMM software. TMHMM also predicts that the regions of the protein containing the Ig domains are likely to be extracellular. ACA1_290100 contains three potential transmembrane helices followed by a short predicted intracellular region. This intracellular tail contains a DnaJ domain with the diagnostic central HPD motif. DnaJ domains have been shown to bind to Hsp70 proteins and to be important in processes such as translation, folding, unfolding, translocation and protein degradation. This protein is the first example of an immunoglobulin domain associated with a DnaJ domain. Our findings are consistent with a deeper evolutionary origin of the immunoglobulin than previously thought. Our results suggest that the lack of immunoglobulin domains in fungi may be due to their lineage specific loss. It will be interesting to see what the molecular roles of the immunoglobulin domains are in *Ac*.

6 Microbial Recognition

Accession Number	Domain Description	IPR	Secondary IPR	Secondary Domain Description
ACA1_388520	LBP/BPI			
ACA1_388570	LBP/BPI			
ACA1_212480	LBP/BPI			
ACA1_238450	LBP/BPI			
ACA1_147410	LBP/BPI			
ACA1_374090	LBP/BPI			
ACA1_030890	D-galactoside/L-rhamnose binding SUEL lectin	IPR000922	IPR000742	Epidermal growth factor-like domain
ACA1_031020	D-galactoside/L-rhamnose binding SUEL lectin	IPR000922	IPR000742	Epidermal growth factor-like domain
ACA1_323710	D-galactoside/L-rhamnose binding SUEL lectin	IPR000922	IPR000742	Epidermal growth factor-like domain
ACA1_048940	D-galactoside/L-rhamnose binding SUEL lectin	IPR000922	IPR000742	Epidermal growth factor-like domain
ACA1_058690	D-galactoside/L-rhamnose binding SUEL lectin	IPR000922	IPR000742	Epidermal growth factor-like domain
ACA1_061070	D-galactoside/L-rhamnose binding SUEL lectin	IPR000922	IPR000742	Epidermal growth factor-like domain
ACA1_188250	D-galactoside/L-rhamnose binding SUEL lectin	IPR000922	IPR000408	Regulator of chromosome condensation, RCC1
ACA1_205380	D-galactoside/L-rhamnose binding SUEL lectin	IPR000922 (4x copies)		
ACA1_048270	D-galactoside/L-rhamnose binding SUEL lectin	IPR000922		
ACA1_030640	D-galactoside/L-rhamnose binding SUEL lectin	IPR000922		
ACA1_391500	C-type lectin			
ACA1_141890	Legume lectin	IPR001220	IPR002931	Transglutaminase-like
ACA1_383210	H-type lectin	IPR019019 (3x copies)	IPR001370	Baculoviral inhibition of apoptosis protein repeat
ACA1_383880	Thaumatin			
ACA1_344490	Capsid proteins			

Table S6.1: Predicted pattern-recognition receptors (PRRs) in *Ac* genome

6.1 Mannose binding protein

The published Mannose binding protein (MBP) was not present in the dataset but could be identified in other publicly available *Ac* genome data (http://www.hgsc.bcm.tmc.edu/microbial-detail.xsp?project_id=163).

6.2 Bactericidal/permeability-increasing protein (BPI)/lipopolysaccharide binding protein (LBP)

Upon infection by Gram-negative bacteria, animals regulate their immune response by using two closely related lipopolysaccharide (LPS)-interacting proteins: LPS-binding protein (LBP) and bactericidal/permeability-increasing protein (BPI) [90]. BPI comprises two tandem domains of the same fold, each possessing a tubular cavity for binding hydrophobic ligands; LBP is thought to bind LPS in a similar fashion [91]. Despite exhibiting the same fold, these tandem domains show no sequence similarity indicative of a common ancestry. In line with this, the N- and C-terminal domains have been termed tubular lipid-binding (TULIP) and TULIP-like domain, respectively [92, 93]. Close homologs of BPI were grouped into the BPI-like family. TULIP domains are found in two additional, remotely homologous groups of proteins, the Takeout- and SMP domain-like families. These three families constitute the TULIP domain superfamily [92, 93].

We used the homology detection tool HHpred [94, 95] to query the PDB70 database clustered at 70% sequence identity (PDB70; as available on 2012-04-05) on the MPI toolkit [96] using the full-length protein, the TULIP domain, and the TULIP-like domain of each of the six proteins (ACA1_147410, ACA1_212480, ACA1_238450, ACA1_374090, ACA1_388520, and ACA1_388570). The full-length proteins had end-to-end matches to human BPI (PDB 1ewf) and a close BPI homolog, cholesterol ester transfer protein (CETP, PDB 2obd), with high significance. The C-terminal halves (TULIP-like domain) of these proteins matched only the TULIP-like domains in BPI and CETP, which is a feature of the BPI-like family [92, 93]. These findings clearly identify the *Ac* proteins as homologues of the BPI-like family members. Five of the proteins contain an N-terminal signal peptide indicating their secretion (Figure

S6.2.1). The homology of the six proteins to the BPI-like family, which has several characterized hydrophobic ligand-binding members, suggests that they may bind hydrophobic ligands. In addition, most of these proteins seem to be secreted, which potentially means that they might function in recognizing or binding bacteria, e.g. when the amoeba encounters biofilms.

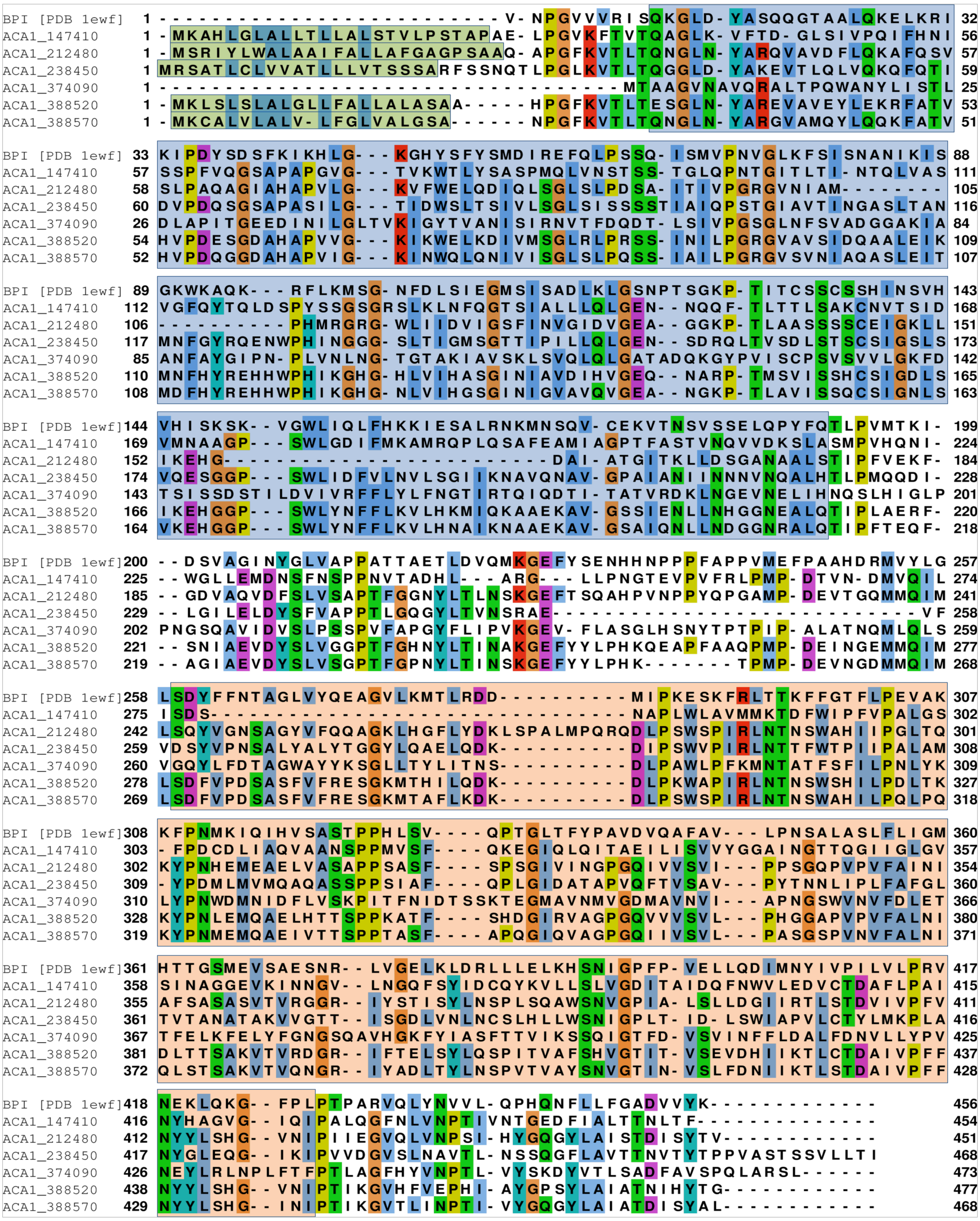

Figure S6.2.1: Multiple sequence alignment of BPI (PDB 1ewf) and the *Ac* homologues. Conserved residues are coloured in the ClustalX colour scheme of JalView. Dashes represent gaps. Background colours are green for signal peptides, blue for TULIP domains, and orange for TULIP-like domains. Uncoloured regions correspond to the collar domain that connects TULIP and TULIP-like domains in BPI. Sequence indices are given to the left and right of each line.

6.3 *D-galactoside/L-rhamnose binding SUEL lectin domain containing proteins*

We identified a number of D-galactoside/L-rhamnose binding SUEL lectins within the genome using interproscan (accession IPR000922).

7 Metabolism

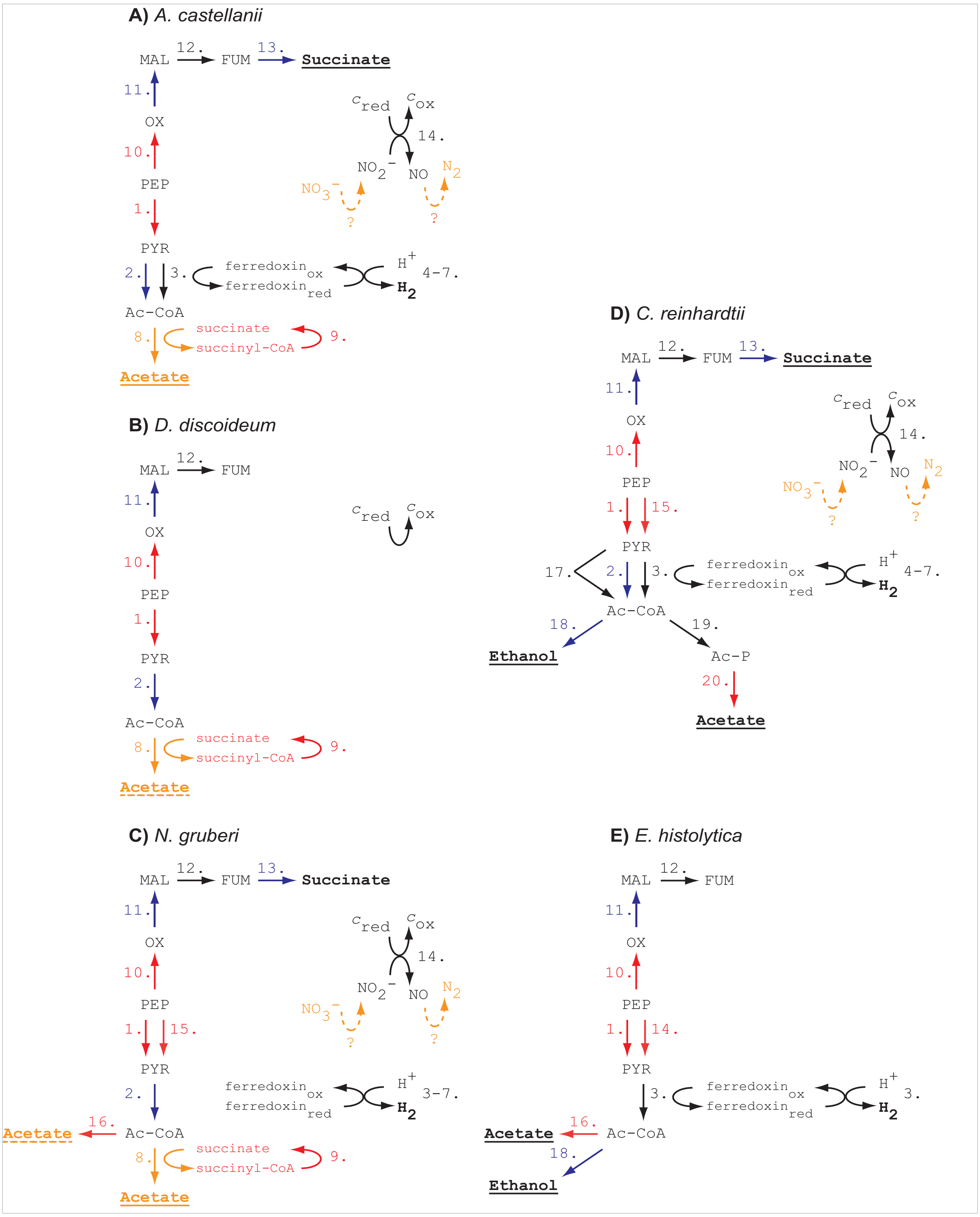

Figure S7.1: The presence or absence of classic biosynthetic pathways in *A. castellanii* and other select soil-dwelling protists. *A. castellanii* is viewed as an organism with an aerobic metabolism (*e.g.* [97]); encoded within

its nuclear genome are enzymes necessary to support the complete catabolism of carbohydrates and other substrates to CO_2 via mitochondrial TCA cycle and respiratory chain activity. Here, attention is focused on the prediction of another aspect to energy metabolism in *Ac*, energy metabolism under anaerobic conditions, which is likely to be important in soil where transient hypoxia can be common. Comparative analysis of metabolic networks for anaerobic/microaerophilic ATP production in *Acanthamoeba* (A), other soil-dwelling protists (B-D), and the parasitic amoebozoan *Entamoeba histolytica* (E). Networks in A-C are predicted based on genome data; for *Chlamydomonas reinhardtii* and EH genome-led predictions are supported by direct experimental analysis. Reactions and enzymes highlighted in red generate ATP/GTP by substrate level phosphorylation. Blue denotes redox reactions; redox-sensitive enzymes shown generally catalyse reversible reactions although monomeric fumarate reductase [enzyme 13.] will operate in the direction shown if fumarate is used as an alternative electron sink to O_2, most probably oxidising NADH directly although use of (rhodo)quinone as the electron donor cannot be ruled out without direct experimentation. Yellow denotes more tentative predictions for metabolic reactions where either the substrate specificity of candidate enzymes cannot be predicted solely from their amino acid sequence (*e.g.* candidate acetate:succinate CoA transferases [enzyme 8.]), or in the case of putative anaerobic respiration it is not known whether the protists shown are capable of NO_3^- respiration or denitrification (here, the molecular identity of the necessary enzymes in eukaryotes are not known). Metabolites underlined denote known (D-E) or likely (A-C) secreted end products of metabolism. Absence of an anaerobic ATP-generating network in *Dd* is consistent with its ecological niche; multiple routes for anaerobic ATP generation are available in other soil-dwelling protists for which genome sequences are available although each possesses its own distinctive network. Anaerobic ATP production in *Ac* is not particularly similar to EH. Key to abbreviations: Ac-CoA, acetyl-CoA; Ac-P, acetyl-phosphate; *c*, cytochrome *c*; FUM, fumarate; MAL, malate; OX, oxaloacetate; PEP, phosphor-enol pyruvate; PYR, pyruvate. Key to enzymes: 1., pyruvate kinase; 2., pyruvate dehydrogenase; 3., pyruvate:ferredoxin oxidoreductase; 4., FeFe-hydrogenase; 5., HydE; 6., HydF; 7., HydG; 8., acetate:succinate CoA transferase; 9., succinyl-CoA synthetase; 10., phospho-enol pyruvate carboxykinase; 11., malate dehydrogenase; 12, fumarase; 13, fumarate reductase; 14., NirK nitrite reductase; 15., pyruvate phosphate dikinase; 16., acetyl-CoA synthetase (ADP-forming); 17., pyruvate:formate lyase; 18., alcohol dehydrogenase E; 19., phospho-acetyl transferase; 20., acetate kinase.

	Ac	*Dd*	*Eh*	*Ng*	*Cr*
Purine biosynthesis	+	+	−	−	+
Pyrimidine biosynthesis	+	+	−	+	+
Gluconeogenesis	+	+	−	−[a]	+
Glycogen metabolism	+	+	?	−	−
Glyoxylate cycle	+	+	−	−	+
Fatty acid biosynthesis	+	−[b]	−	−[b]	+
Mitochondrial type II fatty acid biosynthesis	+	+	−	+	+
Sterol biosynthesis	+	+	−	+	+
Polyketide biosynthesis	+	+++	+	+	+
Heme biosynthesis	+	+	−	−[c]	+
Shikimate pathway	+	−	−	−	+

Table S7.1: The presence or absence of classic biosynthetic pathways was determined in *A. castellanii* (*Ac*), other, select soil-dwelling protists for which complete genome sequences were available (*Dictyostelium discoideum* (*Dd*), *Naegleria gruberi* (*Ng*) *Chlamydomonas reinhardtii* (*Cr*)) and the parasitic amoebozoan *Entamoeba histolytica* (*Eh*). [a]No homologue from any of the four known classes of fructose-1,6- bisphosphatase is found in *Naegleria*, consistent with glucose autotrophy in axenic culture. [b]Yet requires no lipid for axenic culture suggesting the presence of an alternative pathway for bulk fatty acid synthesis in *Dictyostelium* and *Naegleria*. [c]*Naegleria* nonetheless contains ferrochelatase and O_2-independent coproporhyrinogen oxidase homologs; the function of these proteins in the absence of a complete heme biosynthetic pathway is not known.

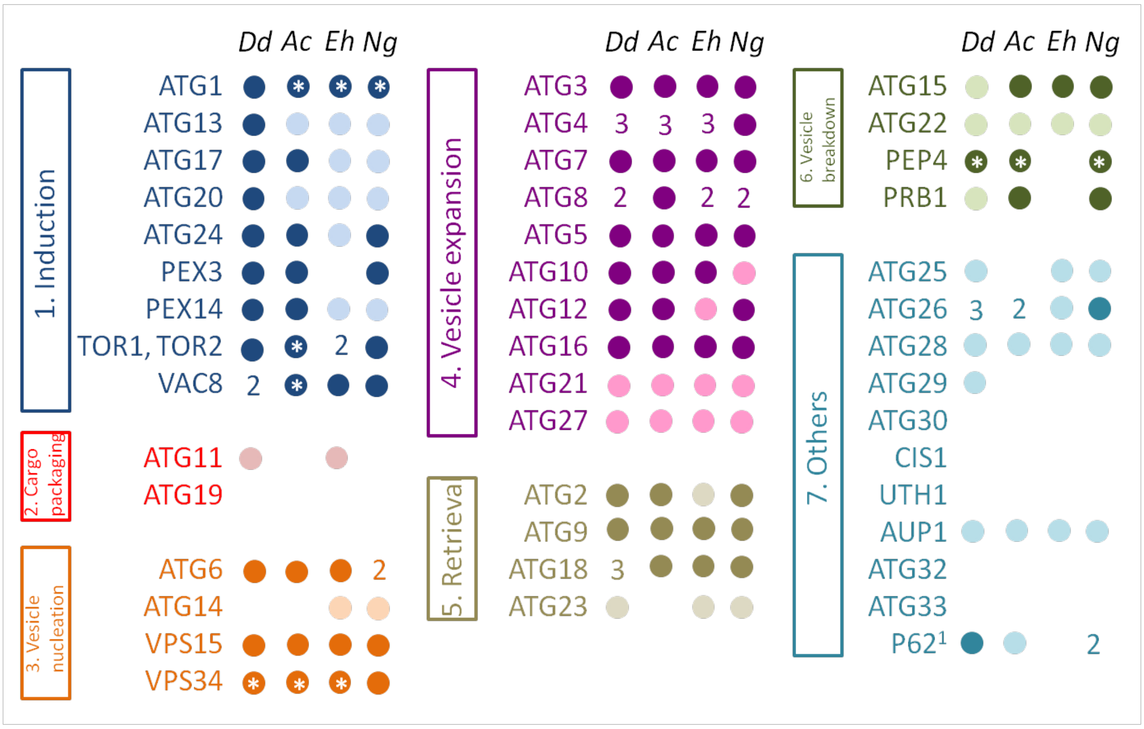

Figure S7.2: Conservation of the macro-autophagy pathway in *Ac*. Candidate orthologues for known autophagy gene products was identified in *Ac* using an identical reciprocal BLAST approach to that used in previous similar analyses [98, 99]. For the analysis of *Ac* candidate autophagy-related genes identified in *Naegleria gruberi: Ng* were also included in the search queries. Dark shaded circles indicate the presence of putative orthologues; lighter shaded circles indicate the identification of sequences homologous to autophagy-related genes, but without evidence for orthology. Numbers of predicted paralogous gene sequences identified in *Ac* are indicated where appropriate and asterisks indicate the presence of homologous sequences that could not reliably be predicted as paralogous or not. [1]P62 is not present in *S. cerevisiae*; all other proteins listed here have been characterised in *S. cerevisiae*, a canonical model for autophagy studies. *Ac*, *Acanthamoeba castellanii Dd*, *Dictyostelium discoideum*; *Eh*, *Entamoeba histolytica*; *Ng*, *Naegleria gruberi*.

8 Transcription Factors

IPR	Secondary IPR	Description	*Ac*	*Dd*
IPR011598		Helix-loop-helix DNA-binding	4	2
IPR012890		GC-rich sequence DNA-binding factor-like	1	1
IPR008967		p53-like transcription factor, DNA-binding	11	10
IPR004181		Zinc finger, MIZ-type	1	5
IPR000967		Znf_NFX1	3	2
IPR000679		Zn-finger, GATA type	36	23
IPR009349		Zinc finger, C2HC5-type	2	1
IPR009057		Homeodomain-like	56	52
	IPR012287	Homeodomain-related		
	IPR001356	Homeobox		
	IPR017970	Homeobox_CS		
IPR001005		SANT domain		32
	IPR014778	Myb, DNA-binding	24	28
IPR003347		Transcription factor jumonji, jmjC	24	13
IPR004827		Basic-leucine zipper (bZIP),transcription factor	6	17
	IPR011616	bZIP transcription factor, bZIP_1	6	15
	IPR003958	Transcription factor CBF/NF- Y/archaeal histone	4	8
IPR001289		CCAAT-binding transcription factor, subunit B	3	1
IPR002100		Transcription factor, MADS-box	3	4
IPR003657		DNA-binding WRKY	3	1
PR001138		Fungal transcriptional regulatory protein, N-terminal	23	3
IPR000232		Heat shock factor	1	1
IPR005559		CG-1 DNA-binding domain	1	0
IPR003150		DNA-binding RFX	60	

Table S8.1: Summary of transcription factors in both *Ac* and *Dd*

8.1 RFX transcription factors — identification and analysis

RFX transcription factors (TF) have been found to be ancient gene family in species ranging from unicellular species to humans. In all unicellular species aside from the choanoflagellate *M. brevicollis*, only a single RFX TF gene has been found in the genome [100]. As query sequences we used protein sequences corresponding to known RFX DNA-binding domains extracted from 8 human genes, one fruit fly gene, one *C. elegans* gene, and one budding yeast gene. We retained all hits that showed ≥40% sequence identity and ≥70% query coverage, a condition similar to that applied previously [100]. Using this procedure, we identified 22 putative RFX genes in the *Ac* genome. Alignment of DBDs from these 22 *Ac* sequences with RFX DBDs from the other species (Figure S8.1.1) reveals that all putative *Ac* RFX DBD sequences share well conserved columns with DNA-binding domains of known RFX genes, suggesting that these *Ac* genes are indeed members of the RFX gene family. In particular, in the putative RFX DBD of ACA1_270030, all 9 conserved RFX DBD sites that have direct contact with DNA are conserved [101]

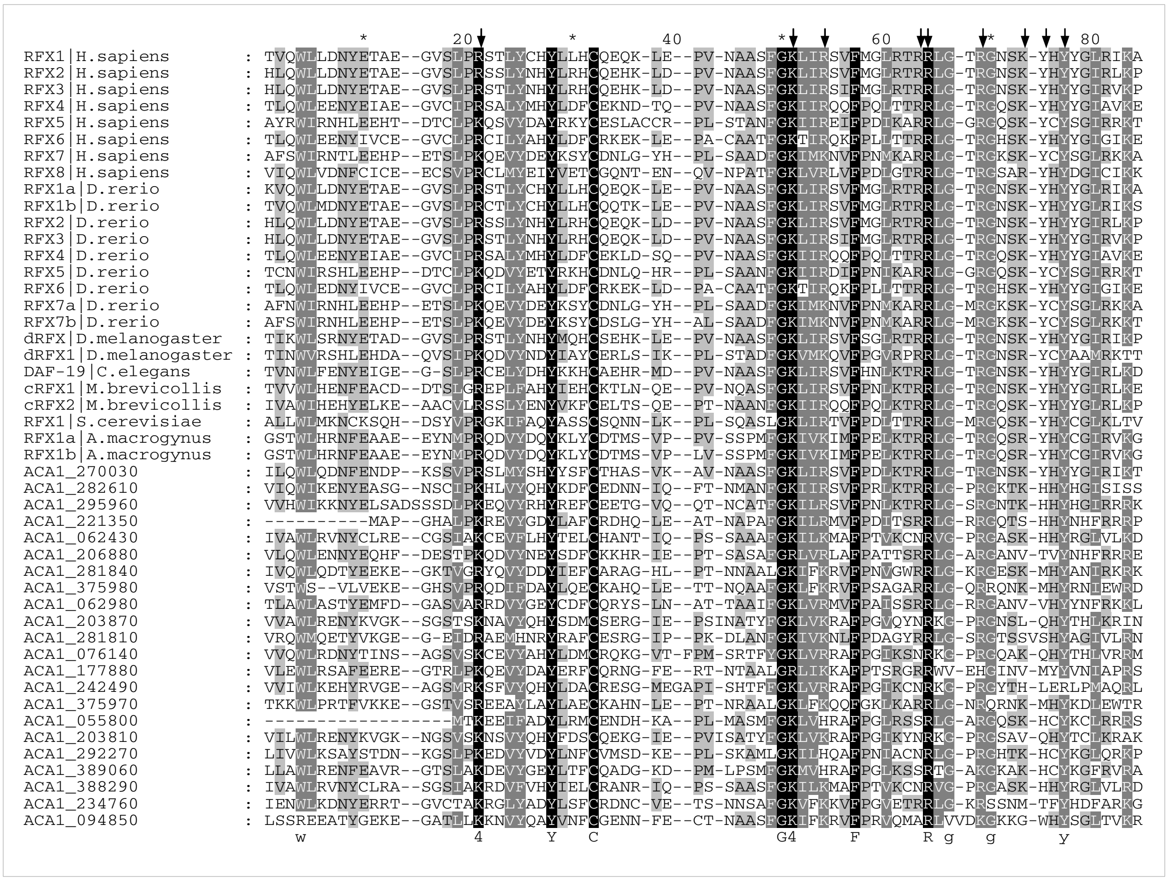

Figure S8.1.1: Putative *Ac* RFX genes. Shown is a multiple sequence alignment of the complete RFX DNA-binding domains of both known and 22 putative new *Ac* RFX genes. Shades of gray indicate the degree of conservation of aligned residues (dark = high conservation, white = no conservation). Arrows indicate the nine residues that according to [101] are in direct contact with DNA. Conserved residues include four of the nine residues making direct contact with DNA as well as a perfectly conserved tyrosine (Y), cytosine (C), glycine (G), and phenylalanine (F). Multiple but imperfectly conserved residues suggest that *Ac* sequences shown here likely belong to true RFX genes, but probably have different binding specificities.

To resolve the evolutionary relationship of putative *Ac* RFX genes among each other and to known RFX genes, we performed a phylogenetic analysis of these sequences. Two *Ac* sequences from our alignment (ACA1_221350 and ACA1_055800) were excluded from phylogenetic analysis due to N-terminal truncations. The resulting phylogenetic tree (Figure S8.2) reveals that three *Ac* genes group with known RFX genes (ACA1_270030, ACA1_295960, and ACA1_282610), one of which (ACA1_270030) groups with human RFX8 and yeast RFX. Remaining RFX DBDs of putative *Ac* genes form a diverse group, suggesting that a potentially ancient paralogous diversification of RFX transcription factors in *Ac*. We believe that the 22 analyzed genes may represent an underestimate as an HMM constructed from the multiple sequence alignment described above revealed a total of 56 hits (E<0.01) in the *Ac* genome using Hmmer3 [102].

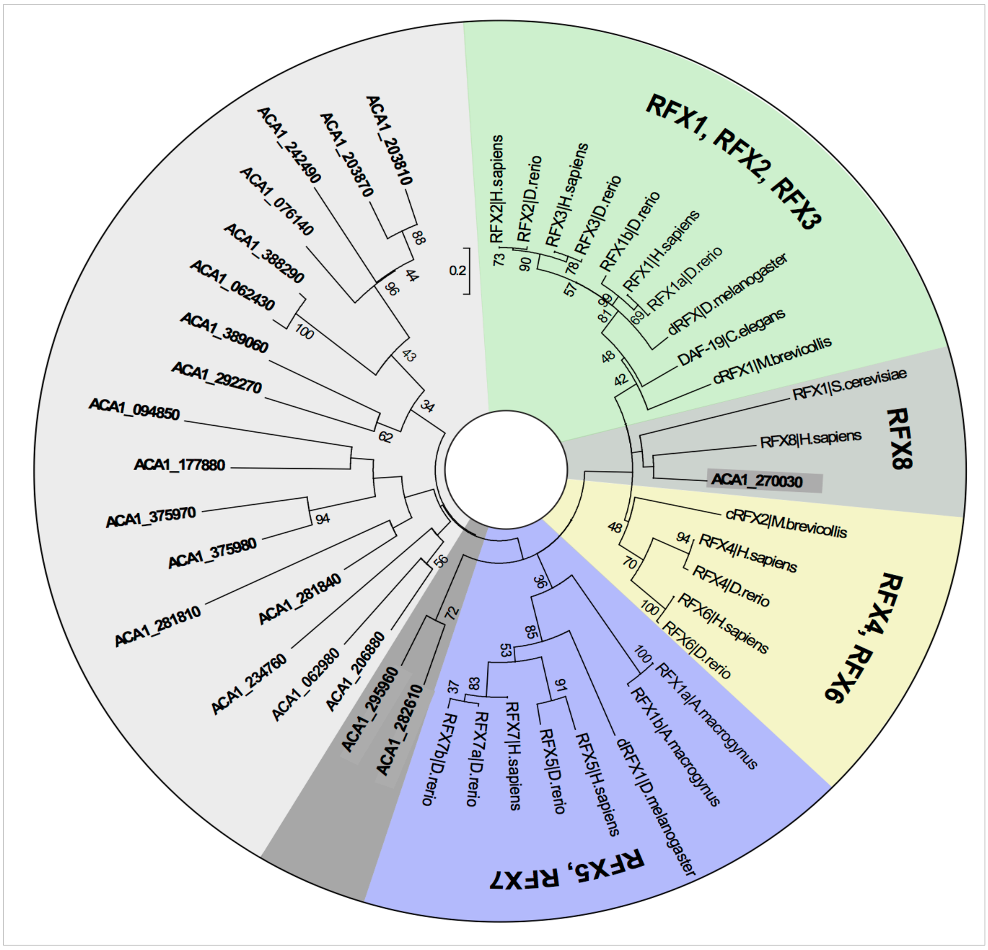

Figure S8.1.2: Most putative *Ac* RFX genes form a diverse out-group to known RFX genes. The phylogenetic tree was inferred from aligned RFX DNA-binding domains (Figure S8.1.1) using the maximum-likelihood method implemented in MEGA5 [54]. Shown is the consensus tree after 100 bootstrap iterations. Numbers at branch points represent bootstrap values, and the scale bar represents number of amino acid substitutions per site. Bootstrap values below 30 not shown. *Ac* genes shown in bold. ACA1_270030 groups with RFX8 and could be orthologous to known RFX genes in the other species.

8.2 *Zinc cluster proteins*

Zinc cluster proteins (or binuclear cluster) form a sub-family of zinc finger proteins with the vast majority of them acting as transcriptional regulators [103, 104]. Zinc cluster proteins possess the well-conserved motif CysX2CysX6CysX5-12CysX2CysX6-8Cys and are found in fungi such as *Saccharomyces cerevisiae* and *Schizosaccharomyces pombe* as well as in human fungal pathogens such as *Candida* species, *Aspergillus* species. However, zinc cluster proteins are absent in prokaryotes or in higher eukaryotes. Using various zinc cluster motifs as queries, we performed a BLAST search of amoebal genomes. Strikingly, a number of ORFs in amoeba fit the consensus sequence of the fungal zinc cluster motif (Figure S8.2.1). The genomes of *Dd, Df* and *Pp* each encode three putative zinc cluster proteins however the *Ac* genome potentially encodes at least 22 zinc cluster proteins (Figure S8.2.1). Zinc cluster proteins are also characterized by the presence of a highly conserved Lys residue between the second and third cysteines - sometimes Arg, His or Gln replaces this conserved amino acid [105]. In *Ac*, all zinc cluster protein have a His residue at that position (with one exception having Lys). In contrast, Lys residues are more prevalent in other amoeba even though His, Met and Asn residues are observed at that position. Two alpha helices are found in the cysteine-rich region [105]. A conserved Pro residue located between the two alpha helices

provides flexibility to the two subdomains [106]. Strikingly, all amoeba zinc cluster proteins possess a Pro residue adjacent to the fourth cysteine. Spacing between the third and fourth cysteines is 5 amino acids for all putative zinc cluster proteins in amoeba. In contrast, in *S. cerevisiae*, the spacing is more variable among zinc cluster proteins. We predict that it is highly likely that *Ac* encodes *bona fide* zinc cluster proteins and it is tempting to speculate that zinc cluster proteins in amoeba are the ancestors of their fungal orthologues.

RACFSCRKAHARCDEERPCRRC----IRTGCES	ACA1_076950
KACTLCRQAHVGCDGGHPCRRCTASGQGDQCRF	ACA1_126420
RACGPCRSAHTACDSQRPCKRCVAAGRADLCVD	ACA1_126440
KACGPCSVAHSACDRVRPCKRCVSQGKAHQCVD	ACA1_126460
RSCNLCRQSHTACETTRPCKRCVRIGKAQLCAD	ACA1_126940
RACLPCRLSHLACDSSRPCKRCTSSGKAAQCVD	ACA1_380040
KACNYCRNSHVACDGERPCGRCVKRGVADHCCD	ACA1_078740
GPCMTCRLRHVLCDRQRPCGRCARLGETDHCVD	ACA1_188240
QACVLCRIAHTACTGTKPCRRCVTEGTPDKCQF	ACA1_159610
KACNFCRISHTACGPERPCRRCIKIGKAHLCSD	ACA1_330830
KACTCCREAHSACDSERPCRRCVRLGRAHLCSD	ACA1_053690
RACNFCRAAHISCETARPCQRCVRLGRGEWCCD	ACA1_053860
TACGTCRLAHASCDRAQPCGRCVRLGKTETCQP	ACA1_401370
KACATCRLAHVSCDREQPCARCVRVGKAESCQP	ACA1_401470
KACLPCRLVHATCNGERPCKRCNALGRPDHCVN	ACA1_168440
RACNLCRLSHTACESTRPCRRCIAIGKAHLCAD	ACA1_075770
LACFACREAHTACSKERPCKRC--ASLGKACYD	ACA1_075800
SACLNCRKRKTGCDAFRPCTKCVRNGLESTCMD	ACA1_064140
KACDNCRTRHSRCDGKEPCAPC--SKKGFQCGY	ACA1_396880
KACATCRLAHVSCDRNQPCARCMRLGKTDSCQP	ACA1_147200
RACAECKESHTACDTERPCARCVSLGLGDSCRD	ACA1_377880
AACSNCRSSHVACSHEIPCKRCVEHGLADSCQY	ACA1_096240
RACLNCRSSKVACDHQRPCVRCTKNGTEDTCRD	*D. discoideum*
KSCFLCQKDMVECDEKTPCSRCIIKGVPQMCYS	*D. discoideum*
RACLNCRNSKVACDQQRPCTRCVKQEIGQTCYD	*D. discoideum*
RACVNCRQSKVACDQQRPCTRCTKHGIEDKCHD	*D. fasciculatum*
RACLNCRNSKVACDPSRPCTRCVKLDMGHSCYD	*D. fasciculatum*
RSCFLCQKEHLECDEQTPCGNCALKGTPQMCFY	*D. fasciculatum*
RACLNCRNSKVACDQQRPCTRCVKQEIGQTCYD	*D. purpureum*
KACFLCQKDMVECDERTPCSRCIIKGVPHMCFS	*D. purpureum*
RACLNCRNSKVACDQQRPCTRCVKQEIGHTCYD	*P. pallidum*
KACFMCQLDNGLCDENHPCSRCVQKGQPQMCYF	*P. pallidum*
KACLNCRSSKVACDHNRPCLRCTKHGIEDSCLD	*P. pallidum*

Figure S8.2.1: Alignment of the zinc finger in putative zinc cluster proteins in amoeba and related species. Genomes data are from *Dd* [68] *Pp* [28] *Df* [28] *Dp* [69] and this study. Conserved cysteines are highlighted in yellow while conserved His and Lys residues are shown in turquoise and green, respectively; conserved Pro residues are shown in grey. In fungal species, the consensus of zinc cluster motif: $CX_2CX_6CX_{5-12}CX_2CX_{6-8}C$.

8.3 Homeodomain proteins

Homeodomain proteins, play a prominent role in pattern formation in animals as well as plants [35, 107], but are found in almost all eukaryotes. While it has been estimated that basal single cell eukaryotes have in the order of 2 to 5 homeobox genes [108-111] this small number has substantially expanded in animals and plants; *C. elegans*, *Drosophila melanogaster* and *Arabidopsis thaliana* have each approximately 100 homeobox genes [35, 107]. Sequencing of the genome of *Dd* (AX2) revealed 13 homeobox genes (www.uniprot.org) [68]. However *Ac* appears to encode substantially more homeodomain proteins than expected for a unicellular organism — 25 which is almost double the number identified in *Dd* [107]. Two are of the MEIS and PBC classes respectively, while most represent an expansion in a homologue of Wariai, a regulator of anterior-posterior patterning in *Dictyostelium* [112] (Figures 8.3.1-4). To analyze them, we compiled representative metazoan homeodomain protein sequences in addition to recovering sequences from other

Dictyostelids. A multiple sequence alignment (MSA) of the extracted homeodomain sequences was generated (Figure S8.3.1). In a number of instances two homeodomains occurred in the same protein, they are postfixed with HD1 and HD2. The alignment was used for phylogenetic analyses using Neighbor Joining in ClustalX (Figure S8.3.2) and PhyML (data not shown) as previously described [33-35].

Like most other eukaryotes, *Ac* encodes two fundamentally distinct types of homeodomains, i.e. those with a typical number of 60 residues and those with a 63 amino TALE (three amino acid loop extension) homeodomain. TALE homeodomain proteins are highly conserved in evolution, with two classes in plants, and 5 in [33, 35, 107, 109, 111]. *Ac* has 3 TALE homeobox genes. The phylogenetic analysis shows that two of them, ACA1_001190 and ACA1_119990, are significantly similar to the MEIS and PBC classes of TALE homeodomain proteins, respectively. This similarity is corroborated by MSAs of the full-length protein sequences, which shows that these two proteins contain MEIS-B and PBC-B domains, respectively (Figure S8.3.3; Figure S8.3.4). The third *Ac* TALE sequence (ACA1_026560) appears to be a derived from a MEIS homeobox gene, although it has an unusual threonine (T) residue at position 50 of the homeodomain, which tends to be characteristic for particular types of homeodomains. *Dd* has four TALE homeodomain sequences, two of which (DdHBX-9 and DdHBX-4) are probably MEIS proteins. While DdHBX-4 has an isoleucine (I) at position 50 like MEIS proteins, DdHBX-9 has a threonine like ACA1_026560, suggesting that the latter two may form a distinct family of TALE homeodomain proteins in the amoebae/slime mold clade. DdHBX-3 is probably a PBC class member, since it has a glycine at position 50 of the homeodomain, and the related Dictyostelid homeodomain proteins such as Dfa_DFA_02219 share even weak sequence similarity in the PBC-B domain (Figure S8.3.4). DdHBX-12 is probably a divergent PBC type gene.

One *Ac* homeodomain sequence ACA1_065970 is highly similar to *Dictyostelium* Wariai (69% identical over 58 residues), although it lacks the ankyrin repeats of Wariai (warA). Most of the remaining *Ac* homeobox genes appear to be related to the WAR genes based on the homeodomain (Figure S8.3.1). It appears that a substantial expansion and diversification of this type of homeodomain has taken place in the *Acanthamoeba* lineage.

Overall, the findings present striking evidence of two highly conserved TALE homeobox genes much further back in evolutionary time than hitherto expected. In plants, the MEIS related KNOX genes [108, 109] have been shown to be involved in shoot meristem development [113], PBC/PBX and MEIS genes in animals interact with Hox genes to play crucial roles in anterior posterior patterning [114]. Even in yeast a TALE homeodomain protein interacts with a typical homeodomain protein to specify mating types [115]. The high evolutionary conservation of these TALE genes, in particular also the conservation of the residue at position 50 of the homeodomain, which is critical for DNA-binding [116], hints that some underlying regulatory networks may also have been conserved.

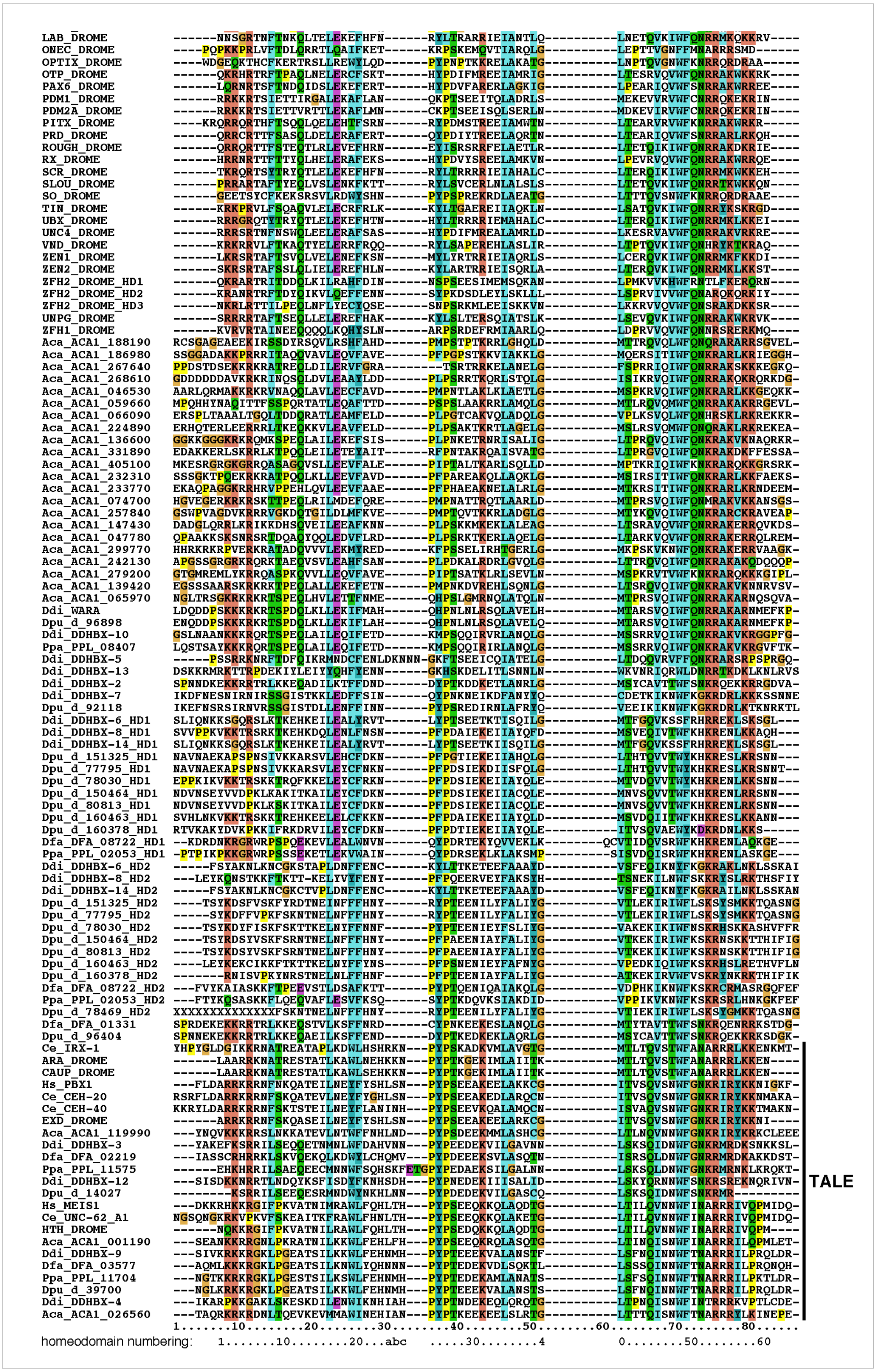

Figure S8.3.1: Multiple sequence alignment of selected homeodomain sequences. "abc" refers to the three extra positions in the TALE homeodomains.

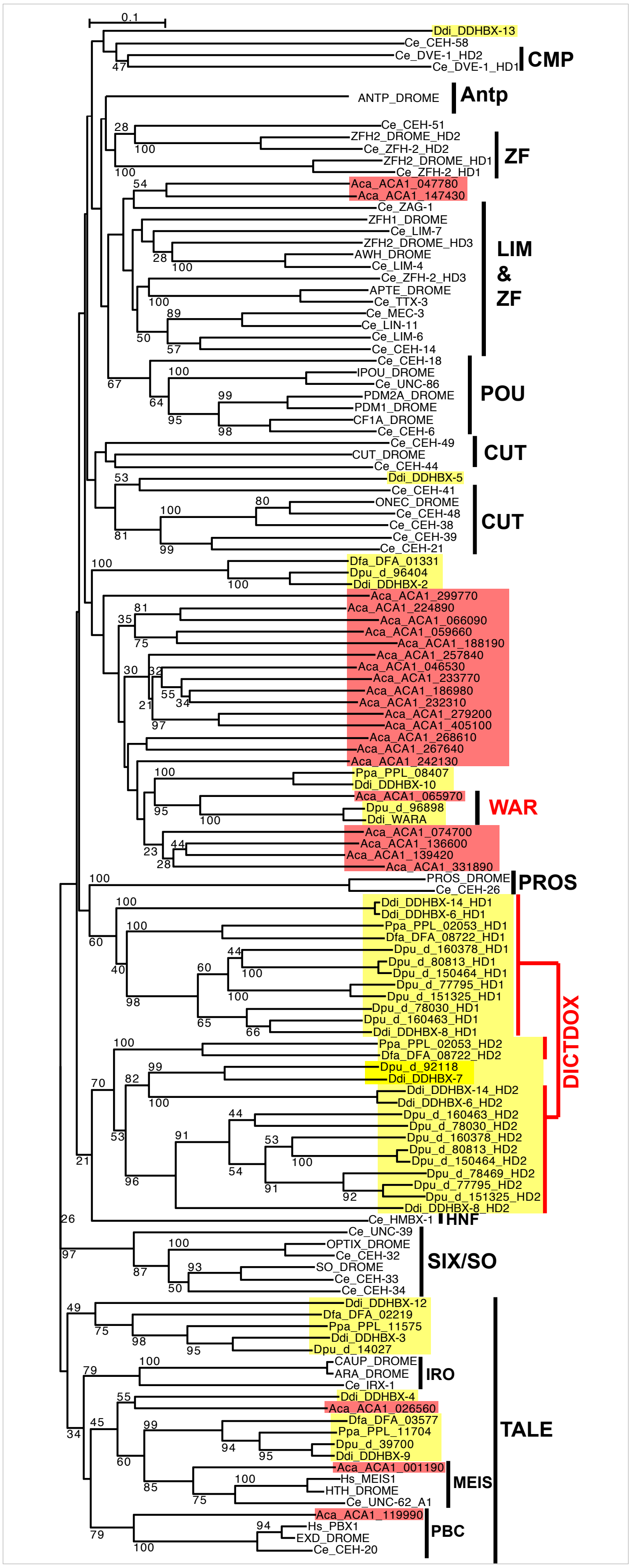

Figure S8.3.2: Neighbor joining phylogenetic tree of homeodomain sequences. Bootstrap values above 20% are shown. The Antennapedia (ANTP) clade has been compressed. Animal homeodomain classes are indicated on

the right. *Ac* sequences are highlighted in red, Dictyostelid sequences in yellow. The Dictyostelium Wariai genes are marked (WAR), and the Dictyostelid double homeobox genes are marked with DictDox (*Dictyostelium* related double homeobox).

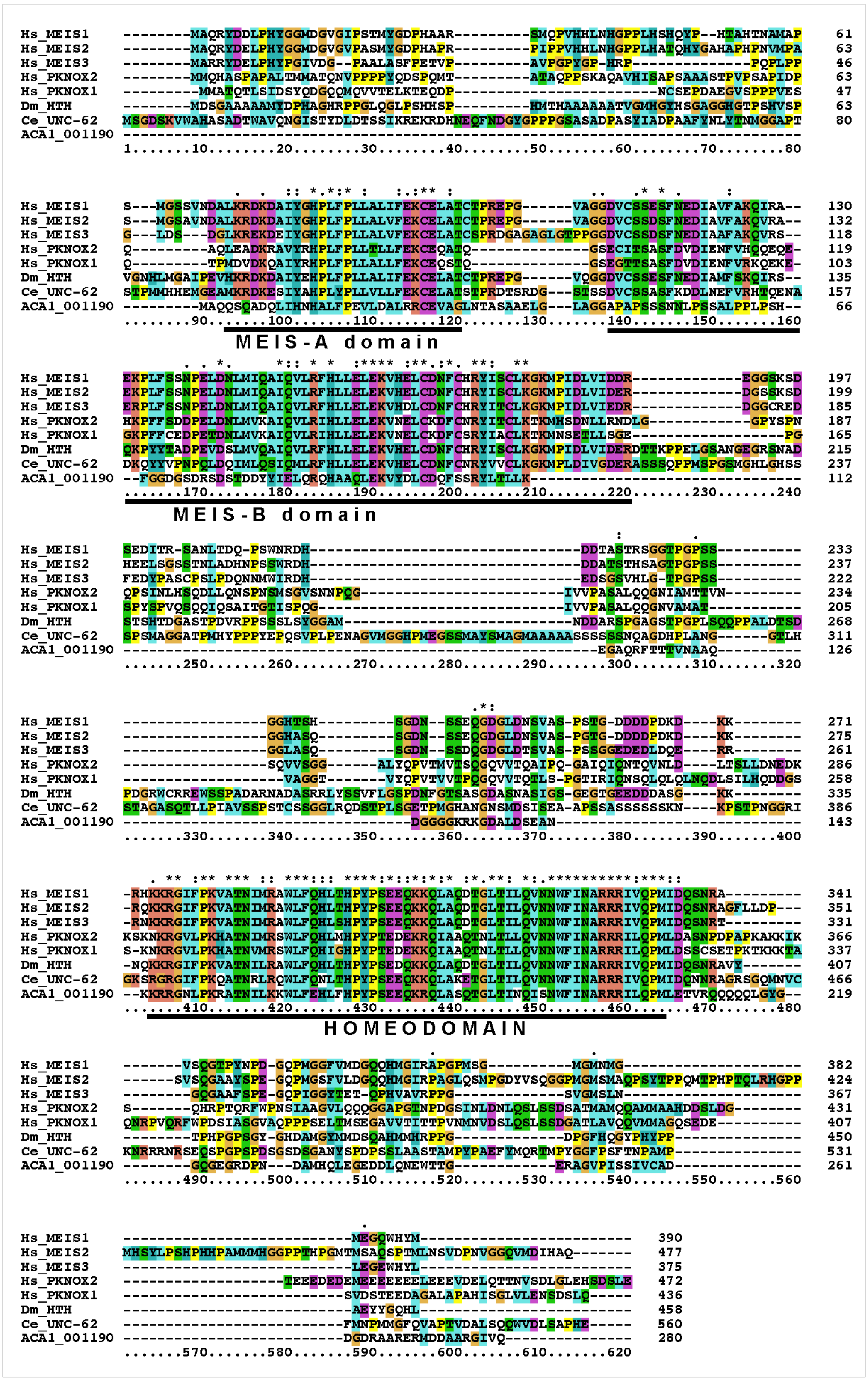

Figure S8.3.3: Multiple sequences alignment of selected MEIS class homeodomain proteins. Note the presence of the MEIS-B domain in ACA1_001190.

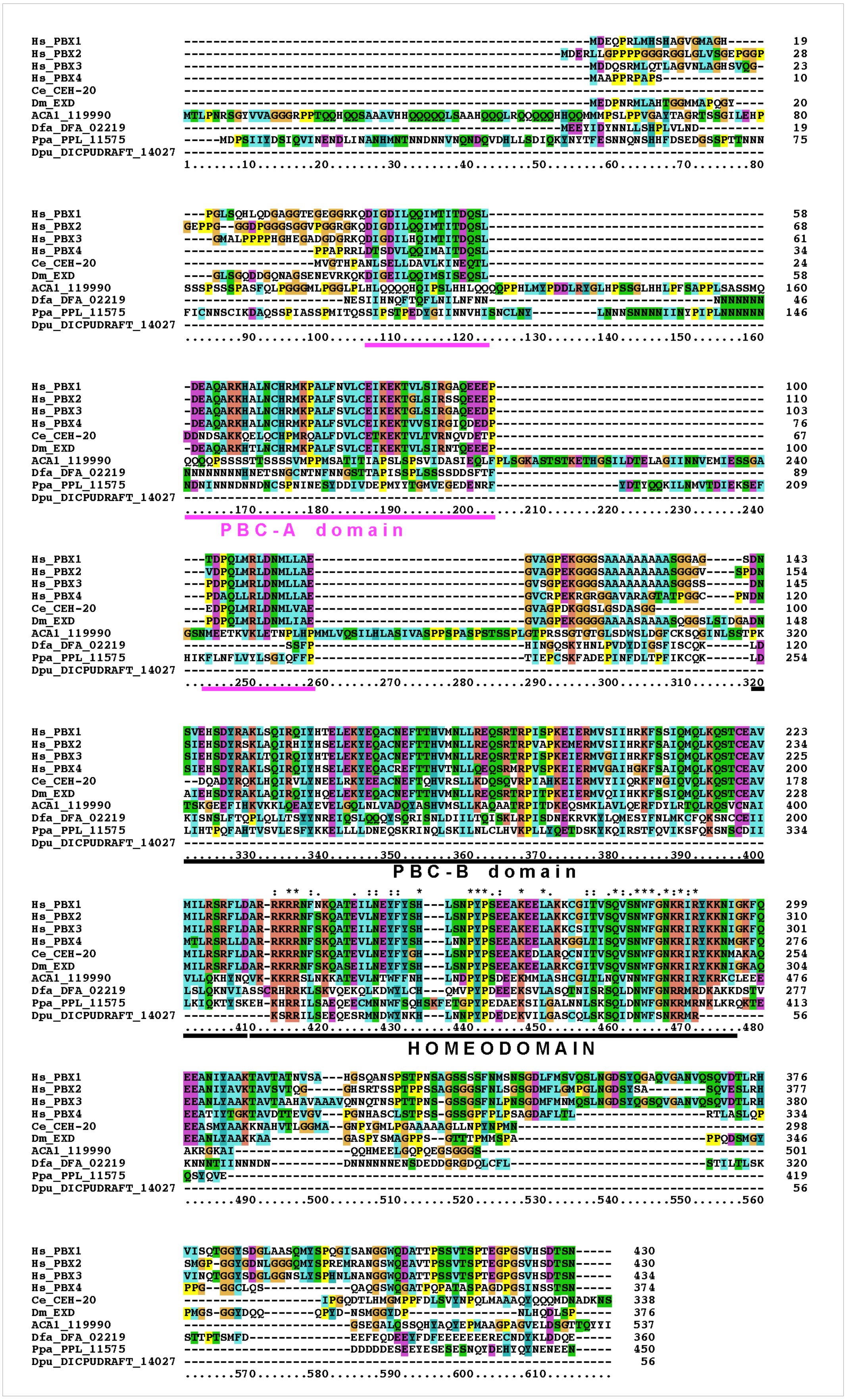

Figure S8.3.4: Multiple sequence alignment of selected PBC class homeodomain proteins. Note that ACA1_119990 aligns well in the PBC-B domain with the animal sequences. Also some of the Dictyostelid

sequences show weak similarity to the PBC-B domain. Further note the conserved glycine (G) residue at position 50 of the homeodomain.

8.4 *Calmodulin-binding transcription activator (CAMTA)*

CAMTAs are calmodulin-binding transcription activators first described in multicellular organisms [117, 118] and subsequently in certain unicellular organisms [118]. In addition to their calmodulin-binding properties, all CAMTAs possess a characteristic DNA-binding domain designated CG1 [118], whose DNA binding specificity has been investigated in *Drosophila* and plants [118]. CAMTAs from multi- and unicellular organisms may also contain TIG, IQ and ANK motifs (explained in [118]). Figure S8.4.1 shows the organisation of the recognized domains in CAMTAs from various multi- and single-cell organisms. The regions defining the important domains in each protein are specified in Figure S8.4.1.

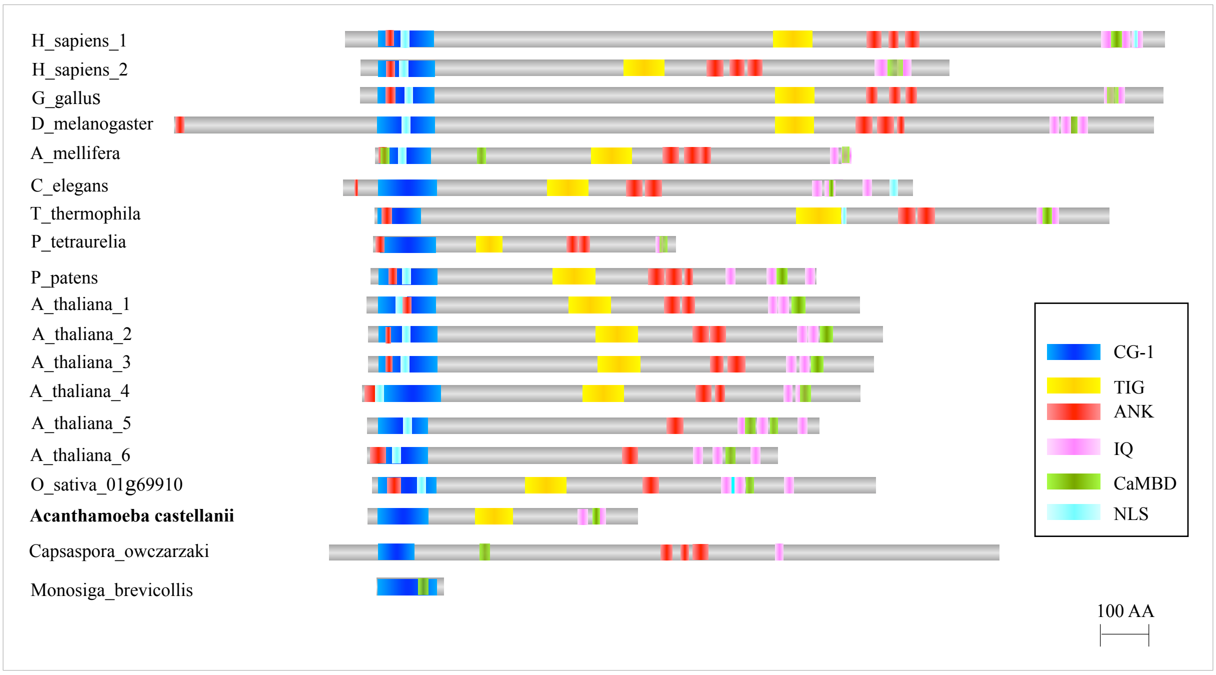

Figure S8.4.1: Domain organisation of *Ac* CAMTA relative to that found in other organisms as per [118].

9 Auxin Related Genes

We examined auxin-related genes in *Ac*. The identification of Indole-3-pyruvate decarboxylase (InterPro: IPR012110) indicates the possibility of presence of the indole-3-pyruvate (IPyA) pathway, which occurs in a broad range of bacteria, including both pathogenic and beneficial bacteria [119]. The presence of nitrilase homologues also indicates possibility of IAA biosynthesis via indole-3acetonitrile (IAN). The discovery of several auxin biosynthesis enzymes in the *Ac* genome indicates the possibility that *Ac* is capable of synthesizing auxin.

In addition to genes involved in auxin biosynthesis we identified the homologues of GH3 (ACA1_300140), which is involved in IAA de-activation via formation of IAA conjugates [120]. Potential functions may include, conversion of free IAA to a storage form, so that the total concentration of free IAA is reduced — this function may benefit the plant if free IAA levels have accumulated to toxic range due to activity of soil bacteria. However, it is also

possible that IAA-conjugates could be exported from *Ac* and the IAA-conjugate absorbed by plant roots, IAA could then released through hydrolyses of the IAA-conjugates. Identification of auxin efflux homologue, ACA1_096600, is strongly suggestive that *Ac* is capable to export free IAA from amoeba cells to influence the growth and development of the plants.

Accession number	InterPro	InterPro description
**Auxin biosynthesis**		
ACA1_099670	IPR012110	Pyruvate decarboxylase/indolepyruvate decarboxylase
ACA1_194150	IPR003010	Nitrilase/cyanide hydratase
ACA1_348680	IPR003010	Nitrilase/cyanide hydratase
**Auxin conjugation**		
ACA1_300140	IPR004993	GH3 auxin-responsive promoter
**Auxin efflux**		
ACA1_096600	IPR004776	Auxin efflux carrier

Table S9.1: Predicted auxin related genes in *Ac*

10 Additional Methods

10.1 A. castellanii growth

A. castellanii strain Neff (ATCC 30010) (kindly provided by M.W Gray) was grown at 30°C with moderate shaking to an O.D_{550} of ~1.0 see [121] for details. Total nucleic acid preparations were depleted of mitochondrial DNA contamination via differential centrifugation of cell extracts; see [121]. High molecular weight DNA was extracted from nuclear pellets either on cesium-Hoechst 33258 dye gradients as per [122] or utilizing the Qiagen Genomic-tip 20/G kit (Qiagen, Hilden, Germany).

10.2 Library preparation and sequencing

All genomic DNA libraries were generated according to the protocol Genomic DNA Sample Prep Guide - Oligo Only Kit (1003492 A) – sonication was substituted for the recommended nebulization as the method for DNA fragmentation utilising a Biorupter™ (Diagenode, Liége, Belgium). The library preparation methodology of end repair to create blunt ended fragments, addition of 3′- A overhang for efficient adapter ligation, ligation of the adapters, size selection of adapter ligated material was carried out utilising enzymes indicated in the protocol. Adapters and amplification primers were purchased from Illumina (Illumina, San Diego, CA, USA) both Single Read Adapters (FC-102-1003) and Paired End Adapters catalogue number PE-102-1003 were used in library construction. All enzymes for library generation were purchased from New England Biolabs (Ipswitch, MA, USA). A limited 14-cycle amplification of size-selected libraries was carried out. To eliminate adapter -dimers libraries were further sized selected 2.5% TAE agarose gels. Purified libraries were quantified using a Qubit™ fluorometer (Invitrogen, Carlsbad, CA, USA) and a Quant-iT™ double-stranded DNA High-Sensitivity Assay Kit (Invitrogen, Carlsbad, CA, USA). Clustering and sequencing of the material was carried out as per manufacturers instructions on the Illumina GAII platform in the UCD Conway Institute (UCD, Dublin Ireland).

11 References

1. Anderson IJ, Watkins RF, Samuelson J, Spencer DF, Majoros WH, Gray MW, Loftus BJ: **Gene discovery in the Acanthamoeba castellanii genome.** *Protist* 2005, **156:**203-214.
2. Phillippy AM, Schatz MC, Pop M: **Genome assembly forensics: finding the elusive mis-assembly.** *Genome Biology* 2008, **9:**R55.
3. Denoeud F, Aury JM, Da Silva C, Noel B, Rogier O, Delledonne M, Morgante M, Valle G, Wincker P, Scarpelli C, Jaillon O, Artiguenave F: **Annotating genomes with massive-scale RNA sequencing.** *Genome Biology* 2008, **9:**R175.
4. Cantarel BL, Korf I, Robb SM, Parra G, Ross E, Moore B, Holt C, Sanchez Alvarado A, Yandell M: **MAKER: an easy-to-use annotation pipeline designed for emerging model organism genomes.** *Genome Research* 2008, **18:**188-196.
5. Lewis SE, Searle SM, Harris N, Gibson M, Lyer V, Richter J, Wiel C, Bayraktaroglir L, Birney E, Crosby MA, Kaminker JS, Matthews BB, Prochnik SE, Smithy CD, Tupy JL, Rubin GM, Misra S, Mungall CJ, Clamp ME: **Apollo: a sequence annotation editor.** *Genome Biology* 2002, **3:**RESEARCH0082.
6. Parkhomchuk D, Borodina T, Amstislavskiy V, Banaru M, Hallen L, Krobitsch S, Lehrach H, Soldatov A: **Transcriptome analysis by strand-specific sequencing of complementary DNA.** *Nucleic Acids Research* 2009, **37:**e123.
7. Weissenmayer BA, Prendergast JG, Lohan AJ, Loftus BJ: **Sequencing illustrates the transcriptional response of Legionella pneumophila during infection and identifies seventy novel small non-coding RNAs.** *PloS One* 2011, **6:**e17570.
8. Lorenzi HA, Puiu D, Miller JR, Brinkac LM, Amedeo P, Hall N, Caler EV: **New assembly, reannotation and analysis of the Entamoeba histolytica genome reveal new genomic features and protein content information.** *PLoS Neglected Tropical Diseases* 2010, **4:**e716.
9. Camon E, Magrane M, Barrell D, Lee V, Dimmer E, Maslen J, Binns D, Harte N, Lopez R, Apweiler R: **The Gene Ontology Annotation (GOA) Database: sharing knowledge in Uniprot with Gene Ontology.** *Nucleic Acids Research* 2004, **32:**D262-266.
10. Abrahamsen MS, Templeton TJ, Enomoto S, Abrahante JE, Zhu G, Lancto CA, Deng M, Liu C, Widmer G, Tzipori S, Buck GA, Xu P, Bankier AT, Dear PH, Konfortov BA, Spriggs HF, Iyer L, Anantharaman V, Aravind L, Kapur V: **Complete genome sequence of the apicomplexan, Cryptosporidium parvum.** *Science* 2004, **304:**441-445.
11. Gardner MJ, Hall N, Fung E, White O, Berriman M, Hyman RW, Carlton JM, Pain A, Nelson KE, Bowman S, Paulsen IT, James K, Eisen JA, Rutherford K, Salzberg SL, Craig A, Kyes S, Chan M-S, Nene V, Shallom SJ, Suh B, Peterson J, Angiuoli S, Pertea M, Allen J, Selengut J, Haft D, Mather MW, Vaidya AB, Martin DMA, et al: **Genome sequence of the human malaria parasite Plasmodium falciparum.** *Nature* 2002, **419:**498-511.
12. Huang J, Mullapudi N, Lancto CA, Scott M, Abrahamsen MS, Kissinger JC: **Phylogenomic evidence supports past endosymbiosis, intracellular and horizontal gene transfer in Cryptosporidium parvum.** *Genome Biology* 2004, **5:**R88.
13. Rattei T, Tischler P, Gotz S, Jehl M-A, Hoser J, Arnold R, Conesa A, Mewes H-W: **SIMAP--a comprehensive database of pre-calculated protein sequence similarities, domains, annotations and clusters.** *Nucleic Acids Research* 2010, **38:**D223-226.
14. Frickey T, Lupas AN: **PhyloGenie: automated phylome generation and analysis.** *Nucleic Acids Research* 2004, **32:**5231-5238.

15. Bray JR, Curtis JT: **An Ordination of the Upland Forest Communities of Southern Wisconsin.** *Ecological Monographs* 1957, **27:**326-349.
16. Joachimiak MP, Weisman JL, May BCH: **JColorGrid: software for the visualization of biological measurement.** *BMC Bioinformatics* 2006, **7**:225
17. Tatusov RL, Koonin EV, Lipman DJ: **A genomic perspective on protein families.** *Science* 1997, **278:**631-637.
18. Powell S, Szklarczyk D, Trachana K, Roth A, Kuhn M, Muller J, Arnold R, Rattei T, Letunic I, Doerks T, Jensen LJ, von Mering C, Bork P: **eggNOG v3.0: orthologous groups covering 1133 organisms at 41 different taxonomic ranges.** *Nucleic Acids Research* 2012, **40:**D284-289.
19. Denoeud F, Henriet S, Mungpakdee S, Aury JM, Da Silva C, Brinkmann H, Mikhaleva J, Olsen LC, Jubin C, Canestro C, Bouquet JM, Danks G, Poulain J, Campsteijn C, Adamski M, Cross I, Yadetie F, Muffato M, Louis A, Butcher S, Tsagkogeorga G, Konrad A, Singh S, Jensen MF, Cong EH, Eikeseth-Otteraa H, Noel B, Anthouard V, Porcel BM, Kachouri-Lafond R et al: **Plasticity of animal genome architecture unmasked by rapid evolution of a pelagic tunicate.** *Science* 2010, **330:**1381-1385.
20. Crick F: **Split genes and RNA splicing.** *Science* 1979, **204:**264-271.
21. Rogers JH: **How were introns inserted into nuclear genes?** *Trends in Genetics* 1989, **5:**213-216.
22. Cavalier-Smith T: **Selfish DNA and the origin of introns.** *Nature* 1985, **315:**283-284.
23. Curtis BA, Archibald JM: **A spliceosomal intron of mitochondrial DNA origin.** *Current Biology* 2010, **20:**R919-920.
24. Irimia M, Rukov JL, Penny D, Vinther J, Garcia-Fernandez J, Roy SW: **Origin of introns by 'intronization' of exonic sequences.** *Trends in Genetics* 2008, **24:**378-381.
25. Catania F, Lynch M: **Where do introns come from?** *PLoS Biology* 2008, **6:**e283.
26. Bockaert J, Pin JP: **Molecular tinkering of G protein-coupled receptors: an evolutionary success.** *The EMBO Journal* 1999, **18:**1723-1729.
27. Fredriksson R, Schiöth HB: **The repertoire of G-protein-coupled receptors in fully sequenced genomes.** *Molecular Pharmacology* 2005, **67:**1414-1425.
28. Heidel AJ, Lawal HM, Felder M, Schilde C, Helps NR, Tunggal B, Rivero F, John U, Schleicher M, Eichinger L, Platzer M, Noegel AA, Schaap P, Glockner G et al: **Phylogeny-wide analysis of social amoeba genomes highlights ancient origins for complex intercellular communication.** *Genome Research* 2011, **21:**1882-1891.
29. Bateman A, Birney E, Durbin R, Eddy SR, Howe KL, Sonnhammer ELL: **The Pfam protein families database.** *Nucleic Acids Research* 2000, **28:**263-266.
30. Chenna R, Sugawara H, Koike T, Lopez R, Gibson TJ, Higgins DG, Thompson JD: **Multiple sequence alignment with the Clustal series of programs.** *Nucleic Acids Research* 2003, **31:**3497-3500.
31. Ronquist F, Huelsenbeck JP: **MrBayes 3: Bayesian phylogenetic inference under mixed models.** *Bioinformatics* 2003, **19:**1572-1574.
32. Schultz J, Milpetz F, Bork P, Ponting CP: **SMART, a simple modular architecture research tool: identification of signaling domains.** *Proceedings of the National Academy of Sciences of the United States of America* 1998, **95:**5857-5864.
33. Mukherjee K, Bürglin TR: **Comprehensive analysis of animal TALE homeobox genes: new conserved motifs and cases of accelerated evolution.** *Journal of Molecular Evolution* 2007, **65:**137-153.
34. Bürglin TR: **Evolution of hedgehog and hedgehog-related genes, their origin from Hog proteins in ancestral eukaryotes and discovery of a novel Hint motif.** *BMC Genomics* 2008, **9:**127.

35. Mukherjee K, Brocchieri L, Bürglin TR: **A comprehensive classification and evolutionary analysis of plant homeobox genes.** *Molecular Biology & Evolution* 2009, **26:**2775-2794.
36. Edgar RC: **MUSCLE: multiple sequence alignment with high accuracy and high throughput.** *Nucleic Acids Research* 2004, **32:**1792-1797.
37. Thompson JD, Gibson TJ, Plewniak F, Jeanmougin F, Higgins DG: **The CLUSTAL_X windows interface: flexible strategies for multiple sequence alignment aided by quality analysis tools.** *Nucleic Acids Research* 1997, **25:**4876-4882.
38. Wolanin PM, Thomason PA, Stock JB: **Histidine protein kinases: key signal transducers outside the animal kingdom.** *Genome Biology* 2002, **3**.
39. Schaller GE, Shiu SH, Armitage JP: **Two-Component Systems and Their Co-Option for Eukaryotic Signal Transduction.** *Current Biology* 2011, **21:**R320-R330.
40. Fritz-Laylin LK, Prochnik SE, Ginger ML, Dacks JB, Carpenter ML, Field MC, Kuo A, Paredez A, Chapman J, Pham J, Shu SQ, Neupane R, Cipriano M, Mancuso J, Tu H, Salamov A, Lindquist E, Shapiro H, Lucas S, Grigoriev IV, Cande WZ, Fulton C, Rokhsar DS, Dawson SC: **The Genome of Naegleria gruberi Illuminates Early Eukaryotic Versatility.** *Cell* 2010, **140:**631-642.
41. Kateriya S, Nagel G, Barnberg E, Hegemann P: **"Vision" in single-celled algae.** *News in Physiological Sciences* 2004, **19:**133-137.
42. Linder JU, Schultz JE: **The class III adenylyl cyclases: multi-purpose signalling modules.** *Cellular Signalling* 2003, **15:**1081-1089.
43. Schaap P: **Guanylyl cyclases across the tree of life.** *Frontiers in Bioscience-Landmark* 2005, **10:**1485-U1485.
44. Söderbom F, Anjard C, Iranfar N, Fuller D, Loomis WF: **An adenylyl cyclase that functions during late development of Dictyostelium.** *Development* 1999, **126:**5463-5471.
45. Saran S, Meima ME, Alvarez-Curto E, Weening KE, Rozen DE, Schaap P: **CAMP signaling in Dictyostelium - Complexity of cAMP synthesis, degradation and detection.** *Journal of Muscle Research & Cell Motility* 2002, **23:**793-802.
46. Gloerich M, Bos JL: **Epac: Defining a New Mechanism for cAMP Action.** *Annual Review of Pharmacology & Toxicology* 2010, **50:**355-375.
47. Meima ME, Biondi RM, Schaap P: **Identification of a novel type of cGMP phosphodiesterase that is defective in the chemotactic stmF mutants.** *Molecular Biology of the Cell* 2002, **13:**3870-3877.
48. Bosgraaf L, Russcher H, Smith JL, Wessels D, Soll DR, Van Haastert PJM: **A novel cGMP signalling pathway mediating myosin phosphorylation and chemotaxis in Dictyostelium.** The *EMBO Journal* 2002, **21:**4560-4570.
49. Conti M, Beavo J: **Biochemistry and physiology of cyclic nucleotide Phosphocliesterases: Essential components in cyclic nucleotide signaling.** *Annual Review of Biochemistry* 2007, **76:**481-511.
50. Spudich JL, Yang CS, Jung KH, Spudich EN: **Retinylidene proteins: structures and functions from archaea to humans.** *Annual Review of Cell & Developmental biology* 2000, **16:**365-392.
51. Sharma AK, Spudich JL, Doolittle WF: **Microbial rhodopsins: functional versatility and genetic mobility.** *Trends in Microbiology* 2006, **14:**463-469.
52. Thompson JD, Higgins DG, Gibson TJ: **CLUSTAL W: improving the sensitivity of progressive multiple sequence alignment through sequence weighting, position-specific gap penalties and weight matrix choice.** *Nucleic Acids Research* 1994, **22:**4673-4680.

53. Hall TA: **BioEdit: a user-friendly biological sequence alignment editor and analysis program for Windows 95/98/NT.** *Nucleic Acids Symposium Series* 1999, **41:**95-98.
54. Tamura K, Peterson D, Peterson N, Stecher G, Nei M, Kumar S: **MEGA5: molecular evolutionary genetics analysis using maximum likelihood, evolutionary distance, and maximum parsimony methods.** *Molecular Biology & Evolution* 2011, **28:**2731-2739.
55. Hwang I, Sheen J: **Two-component circuitry in Arabidopsis cytokinin signal transduction.** *Nature* 2001, **413:**383-389.
56. Hegemann P: **Algal sensory photoreceptors.** *Annual Review of Plant Biology* 2008, **59:**167-189.
57. Miranda-Saavedra D, Barton GJ: **Classification and functional annotation of eukaryotic protein kinases.** *Proteins* 2007, **68:**893-914.
58. Daub H, Specht K, Ullrich A: **Strategies to overcome resistance to targeted protein kinase inhibitors.** *Nature Reviews Drug Discovery* 2004, **3:**1001-1010.
59. Gorre ME, Mohammed M, Ellwood K, Hsu N, Paquette R, Rao PN, Sawyers CL: **Clinical resistance to STI-571 cancer therapy caused by BCR-ABL gene mutation or amplification.** *Science* 2001, **293:**876-880.
60. Azam M, Seeliger MA, Gray NS, Kuriyan J, Daley GQ: **Activation of tyrosine kinases by mutation of the gatekeeper threonine.** *Nature Structural & Molecular Biology* 2008, **15:**1109-1118.
61. Alonso A, Sasin J, Bottini N, Friedberg I, Osterman A, Godzik A, Hunter T, Dixon J, Mustelin T: **Protein tyrosine phosphatases in the human genome.** *Cell* 2004, **117:**699-711.
62. Manning G, Young SL, Miller WT, Zhai Y: **The protist, Monosiga brevicollis, has a tyrosine kinase signaling network more elaborate and diverse than found in any known metazoan.** *Proceedings of the National Academy of Sciences of the United States of America* 2008, **105:**9674-9679.
63. Andersen JN, Jansen PG, Echwald SM, Mortensen OH, Fukada T, Del Vecchio R, Tonks NK, Moller NP: **A genomic perspective on protein tyrosine phosphatases: gene structure, pseudogenes, and genetic disease linkage.** *FASEB Journal* 2004, **18:**8-30.
64. Tan CS, Pasculescu A, Lim WA, Pawson T, Bader GD, Linding R: **Positive selection of tyrosine loss in metazoan evolution.** *Science* 2009, **325:**1686-1688.
65. Remm M, Storm CE, Sonnhammer EL: **Automatic clustering of orthologs and in-paralogs from pairwise species comparisons.** *Journal of Molecular Biology* 2001, **314:**1041-1052.
66. Chisholm RL, Gaudet P, Just EM, Pilcher KE, Fey P, Merchant SN, Kibbe WA: **dictyBase, the model organism database for Dictyostelium discoideum.** *Nucleic Acids Research* 2006, **34:**D423-427.
67. Tan CSH, Schoof EM, Creixell P, Pasculescu A, Lim WA, Pawson T, Bader GD, Linding R: **Response to Comment on "Positive Selection of Tyrosine Loss in Metazoan Evolution".** *Science* 2011, **332:**917.
68. Eichinger L, Pachebat JA, Glockner G, Rajandream MA, Sucgang R, Berriman M, Song J, Olsen R, Szafranski K, Xu Q, Tunggal B, Kummerfeld S, Madera M, Konfortov BA, Rivero F, Bankier AT, Lehmann R, Hamlin N, Davies R, Gaudet P, Fey P, Pilcher K, Chen G, Saunders D, Sodergren E, Davis P, Kerhornou A, Nie X, Hall N, Anjard C, et al: **The genome of the social amoeba Dictyostelium discoideum.** *Nature* 2005, **435:**43-57.

69. Sucgang R, Kuo A, Tian X, Salerno W, Parikh A, Feasley CL, Dalin E, Tu H, Huang E, Barry K, Lindquist E, Shapiro H, Bruce D, Schmutz J, Salamov A, Fey P, Gaudet P, Anjard C, Babu MM, Basu S, Bushmanova Y, van der Wel H, Katoh Kurasawa M, Dinh C, Coutinho PM, Saito T, Elias M, Schaap P, Kay RR, Henrissat B, et al: **Comparative genomics of the social amoebae Dictyostelium discoideum and Dictyostelium purpureum.** *Genome Biology* 2011, **12:**R20.
70. Liu BA, Jablonowski K, Raina M, Arcé M, Pawson T, Nash PD: **The human and mouse complement of SH2 domain proteins-establishing the boundaries of phosphotyrosine signaling.** *Molecular Cell* 2006, **22:**851-868.
71. Pawson T: **Specificity in signal transduction: from phosphotyrosine-SH2 domain interactions to complex cellular systems.** *Cell* 2004, **116:**191-203.
72. Smith MJ, Hardy WR, Murphy JM, Jones N, Pawson T: **Screening for PTB domain binding partners and ligand specificity using proteome-derived NPXY peptide arrays.** *Molecular & Cellular Biology* 2006, **26:**8461-8474.
73. Benes CH, Wu N, Elia AE, Dharia T, Cantley LC, Soltoff SP: **The C2 domain of PKCdelta is a phosphotyrosine binding domain.** *Cell* 2005, **121:**271-280.
74. Mukherjee M, Chow SY, Yusoff P, Seetharaman J, Ng C, Sinniah S, Koh XW, Asgar NF, Li D, Yim D, Qian J, Iyu A, Lim YP, Zhou X, Sze SK, Guy GR, Sivaraman J: **Structure of a novel phosphotyrosine-binding domain in Hakai that targets E-cadherin.** *The EMBO Journal* 2012, **31:**1308-1319.
75. Liu BA, Shah E, Jablonowski K, Stergachis A, Engelmann B, Nash PD: **The SH2 domain-containing proteins in 21 species establish the provenance and scope of phosphotyrosine signaling in eukaryotes.** *Science Signaling* 2011, **4:**ra83.
76. Moniakis J, Funamoto S, Fukuzawa M, Meisenhelder J, Araki T, Abe T, Meili R, Hunter T, Williams J, Firtel RA: **An SH2-domain-containing kinase negatively regulates the phosphatidylinositol-3 kinase pathway.** *Genes & Development* 2001, **15:**687-698.
77. Lim WA, Pawson T: **Phosphotyrosine signaling: evolving a new cellular communication system.** *Cell* 2010, **142:**661-667.
78. Blom N, Sicheritz-Ponten T, Gupta R, Gammeltoft S, Brunak S: **Prediction of post-translational glycosylation and phosphorylation of proteins from the amino acid sequence.** *Proteomics* 2004, **4:**1633-1649.
79. Huang H, Li L, Wu C, Schibli D, Colwill K, Ma S, Li C, Roy P, Ho K, Songyang Z, Pawson T, Gao Y, Li, SS: **Defining the specificity space of the human SRC homology 2 domain.** *Molecular & Cellular Proteomics* 2008, **7:**768-784.
80. Liu BA, Jablonowski K, Shah EE, Engelmann BW, Jones RB, Nash PD: **SH2 domains recognize contextual peptide sequence information to determine selectivity.** *Molecular & Cellular Proteomics* 2010, **9:**2391-2404.
81. Songyang Z, Shoelson SE, Chaudhuri M, Gish G, Pawson T, Haser WG, King F, Roberts T, Ratnofsky S, Lechleider RJ, Neel BG, Birge RB, Fajardo JE, Chou MM, Hanafusa H, Schaffhausen B, Cantley LC: **SH2 domains recognize specific phosphopeptide sequences.** *Cell* 1993, **72:**767-778.
82. Tsujioka M, Yoshida K, Nagasaki A, Yonemura S, Müller-Taubenberger A, Uyeda TQ: **Overlapping functions of the two talin homologues in Dictyostelium.** *Eukaryotic Cell* 2008, **7:**906-916.
83. Dickinson DJ, Nelson WJ, Weis WI: **A polarized epithelium organized by beta- and alpha-catenin predates cadherin and metazoan origins.** *Science* 2011, **331:**1336-1339.

84. Grimson MJ, Coates JC, Reynolds JP, Shipman M, Blanton RL, Harwood AJ: **Adherens junctions and beta-catenin-mediated cell signalling in a non-metazoan organism.** *Nature* 2000, **408:**727-731.
85. Buljan M, Bateman A: **The evolution of protein domain families.** *Biochemical Society Transactions* 2009, **37:**751-755.
86. King N, Westbrook MJ, Young SL, Kuo A, Abedin M, Chapman J, Fairclough S, Hellsten U, Isogai Y, Letunic I, Marr M, Pincus D, Putnam N, Rokas A, Wright KJ, Zuzow R, Dirks W, Good M, Goodstein D, Lemons D, Li W, Lyons JB, Morris A, Nichols S, Richter DJ, Salamov A. Sequencing JGI, Bork P, Lim WA, Manning G et al: **The genome of the choanoflagellate Monosiga brevicollis and the origin of metazoans.** *Nature* 2008, **451:**783-788.
87. Bateman A, Eddy SR, Chothia C: **Members of the immunoglobulin superfamily in bacteria.** *Protein Science* 1996, **5:**1939-1941.
88. Harpaz Y, Chothia C: **Many of the immunoglobulin superfamily domains in cell adhesion molecules and surface receptors belong to a new structural set which is close to that containing variable domains.** *Journal of Molecular Biology* 1994, **238:**528-539.
89. Waterhouse AM, Procter JB, Martin DMA, Clamp M, Barton GJ: **Jalview Version 2--a multiple sequence alignment editor and analysis workbench.** *Bioinformatics* 2009, **25:**1189-1191.
90. Bingle CD, Craven CJ: **Meet the relatives: a family of BPI- and LBP-related proteins.** *Trends in Immunology* 2004, **25:**53-55.
91. Beamer LJ, Carroll SF, Eisenberg D: **Crystal structure of human BPI and two bound phospholipids at 2.4 angstrom resolution.** *Science* 1997, **276:**1861-1864.
92. Kopec KO, Alva V, Lupas AN: **Homology of SMP domains to the TULIP superfamily of lipid-binding proteins provides a structural basis for lipid exchange between ER and mitochondria.** *Bioinformatics* 2010, **26:**1927-1931.
93. Kopec KO, Alva V, Lupas AN: **Bioinformatics of the TULIP domain superfamily.** *Biochemical Society Transactions* 2011, **39:**1033-1038.
94. Söding J: **Protein homology detection by HMM-HMM comparison.** *Bioinformatics* 2005, **21:**951-960.
95. Söding J, Biegert A, Lupas AN: **The HHpred interactive server for protein homology detection and structure prediction.** *Nucleic Acids Research* 2005, **33:**W244-W248.
96. Biegert A, Mayer C, Remmert M, Soding J, Lupas AN: **The MPI Bioinformatics Toolkit for protein sequence analysis.** *Nucleic Acids Research* 2006, **34:**W335-339.
97. Jarmuszkiewicz W, Sluse-Goffart CM, Hryniewiecka L, Michejda J, Sluse FE: **Electron partitioning between the two branching quinol-oxidizing pathways in Acanthamoeba castellanii mitochondria during steady-state state 3 respiration.** *The Journal of Biological Chemistry* 1998, **273:**10174-10180.
98. Brennand A, Gualdron-Lopez M, Coppens I, Rigden DJ, Ginger ML, Michels PA: **Autophagy in parasitic protists: unique features and drug targets.** *Molecular & Biochemical Parasitology* 2011, **177:**83-99.
99. Rigden DJ, Michels PA, Ginger ML: **Autophagy in protists: Examples of secondary loss, lineage-specific innovations, and the conundrum of remodeling a single mitochondrion.** *Autophagy* 2009, **5:**784-794.
100. Chu JSC, Baillie DL, Chen N: **Convergent evolution of RFX transcription factors and ciliary genes predated the origin of metazoans.** *BMC Evolutionary Biology* 2010, **10:**130.

101. Gajiwala KS, Chen H, Cornille F, Roques BP, Reith W, Mach B, Burley SK: **Structure of the winged-helix protein hRFX1 reveals a new mode of DNA binding.** *Nature* 2000, **403:**916-921.
102. Eddy SR: **A new generation of homology search tools based on probabilistic inference.** *Genome Informatics. International Conference on Genome Informatics* 2009, **23:**205-211.
103. MacPherson S, Larochelle M, Turcotte B: **A fungal family of transcriptional regulators: the zinc cluster proteins.** *Microbiology & Molecular Biology Reviews* 2006, **70:**583-604.
104. Todd RB, Andrianopoulos A: **Evolution of a fungal regulatory gene family: the Zn(II)2Cys6 binuclear cluster DNA binding motif.** *Fungal Genetics & Biology* 1997, **21:**388-405.
105. Schjerling P, Holmberg S: **Comparative amino acid sequence analysis of the C6 zinc cluster family of transcriptional regulators.** *Nucleic Acids Research* 1996, **24:**4599-4607.
106. Marmorstein R, Harrison SC: **Crystal structure of a PPR1-DNA complex: DNA recognition by proteins containing a Zn2Cys6 binuclear cluster.** *Genes & Development* 1994, **8:**2504-2512.
107. Bürglin TR: **Homeodomain subtypes and functional diversity.** *Sub-cellular Biochemistry* 2011, **52:**95-122.
108. Bürglin TR: **Analysis of TALE superclass homeobox genes (MEIS, PBC, KNOX, Iroquois, TGIF) reveals a novel domain conserved between plants and animals.** *Nucleic Acids Research* 1997, **25:**4173-4180.
109. Bürglin TR: **The PBC domain contains a MEINOX domain: coevolution of Hox and TALE homeobox genes?** *Development Genes & Evolution* 1998, **208:**113-116.
110. Bürglin TR: **The homeobox genes of Encephalitozoon cuniculi (Microsporidia) reveal a putative mating-type locus.** *Development Genes & Evolution* 2003, **213:**50-52.
111. Derelle R, Lopez P, Le Guyader H, Manuel M: **Homeodomain proteins belong to the ancestral molecular toolkit of eukaryotes.** *Evolution & Development* 2007, **9:**212-219.
112. Han Z, Firtel RA: **The homeobox-containing gene Wariai regulates anterior-posterior patterning and cell-type homeostasis in Dictyostelium.** *Development* 1998, **125:**313-325.
113. Hay A, Tsiantis M: **KNOX genes: versatile regulators of plant development and diversity.** *Development* 2010, **137:**3153-3165.
114. Mann RS, Affolter M: **Hox proteins meet more partners.** *Current Opinion in Genetics & Development* 1998, **8:**423-429.
115. Ho CY, Adamson JG, Hodges RS, Smith M: **Heterodimerization of the yeast MATa1 and MAT alpha 2 proteins is mediated by two leucine zipper-like coiled-coil motifs.** *The EMBO Journal* 1994, **13:**1403-1413.
116. Gehring WJ, Affolter M, Bürglin T: **Homeodomain proteins.** *Annual Review of Biochemistry* 1994, **63:**487-526.
117. Bouché N, Scharlat A, Snedden W, Bouchez D, Fromm H: **A novel family of calmodulin-binding transcription activators in multicellular organisms.** *The Journal of Biological Chemistry* 2002, **277:**21851-21861.
118. Finkler A, Ashery-Padan R, Fromm H: **CAMTAs: calmodulin-binding transcription activators from plants to human.** *FEBS Letters* 2007, **581:**3893-3898.
119. Spaepen S, Vanderleyden J: **Auxin and Plant-Microbe Interactions.** *Cold Spring Harbor Perspectives in Biology* 2011, **3**.

120. Woodward AW, Bartel B: **Auxin: Regulation, action, and interaction.** *Annals of Botany* 2005, **95:**707-735.
121. Lohan AJ, Gray MW: **Analysis of 5'- or 3'-terminal tRNA editing: mitochondrial 5' tRNA editing in Acanthamoeba castellanii as the exemplar.** *Methods in Enzymology* 2007, **424:**223-242.
122. Spencer DF, Schnare MN, Gray MW: **Isolation of wheat mitochondrial DNA and RNA.** In *Modern Methods of Plant Analysis New Series. Volume* 14. Edited by Linskens HF, Jackson JF: Springer-Verlag, Berlin; 1992: 347-360: *Modern Methods of Plant Analysis*.
